# Design of a Tool Capable of Assessing Environmental Sociocultural Physical Factors Influencing Women’s Decisions on When and Where to Toilet Within Real-World Settings: Protocol for the Build and Usability Testing of a Mobile App for Use by Community-Dwelling Women

**DOI:** 10.2196/54046

**Published:** 2024-09-18

**Authors:** Janis M Miller, Jean F Wyman, Lawrence An, Haitao Chu, Cynthia S Fok, Missy Lavender, Cora Elizabeth Lewis, Alayne D Markland, Leslie M Rickey, Ying Sheng, Siobhan Sutcliffe, Lisa Kane Low, Elizabeth R Mueller, The Prevention of Lower Urinary Tract Symptoms (PLUS) Research Consortium

**Affiliations:** 1 Department of Health Behavior and Biological Sciences School of Nursing University of Michigan Ann Arbor, MI United States; 2 Department of Obstetrics and Gynecology School of Medicine University of Michigan Ann Arbor, MI United States; 3 School of Nursing University of Minnesota Minneapolis, MN United States; 4 Department of Internal Medicine University of Michigan Ann Arbor, MI United States; 5 Center for Health Communications Research University of Michigan Ann Arbor, MI United States; 6 Division of Biostatistics School of Public Health University of Minnesota Minneapolis, MN United States; 7 Department of Urology University of Minnesota Minneapolis, MN United States; 8 Renalis Health Cleveland, OH United States; 9 Department of Epidemiology School of Public Health University of Alabama at Birmingham Birmingham, AL United States; 10 Division of Gerontology, Geriatrics, and Palliative Care Department of Medicine, Heersink School of Medicine University of Alabama at Birmingham Birmingham, AL United States; 11 Birmingham/Atlanta Veteran’s Affairs Geriatric Research Educational and Clinical Center Birmingham, AL United States; 12 Department of Urology Yale University New Haven, CT United States; 13 Department of Obstetrics, Gynecology and Reproductive Sciences Yale University New Haven, CT United States; 14 School of Nursing Vanderbilt University Nashville, TN United States; 15 Public Health Sciences Division Department of Surgery Washington University School of Medicine in St. Louis St. Louis, MO United States; 16 Department of Surgery Washington University School of Medicine St. Louis, MO United States; 17 Department of Obstetrics and Gynecology Washington University School of Medicine St. Louis, MO United States; 18 Division of Female Pelvic Medicine and Reconstructive Surgery Department of Obstetrics and Gynecology Loyola University Chicago Chicago, IL United States; 19 Female Urology/Pelvic Medicine & Reconstructive Surgery Loyola University Medical Center Maywood, IL United States; 20 See Acknowledgments

**Keywords:** mobile app, urinary bladder, woman’s health, toileting, ecological momentary assessment, time factors, population studies, real-world environment, mobile phone

## Abstract

**Background:**

Although surveys and apps are available for women to report urination and bladder symptoms, they do not include their decisions regarding toileting. Real-world factors can interfere with toileting decisions, which may then influence bladder health. This premise lacks data per want of a robust data collection tool.

**Objective:**

The Prevention of Lower Urinary Tract Symptoms (PLUS) research consortium engaged a transdisciplinary team to build and test WhereIGo, a mobile data collection app for Android and iOS. The design goal was a comprehensive reporting system for capturing environmental, sociocultural, and physical factors that influence women’s decisions for toileting. Aims include having (1) an innovative feature for reporting physiologic urge sensation when “thinking about my bladder” and shortly before “I just peed,” (2) real-time reporting along with short look-back opportunities, and (3) ease of use anywhere.

**Methods:**

The development team included a plain language specialist, a usability specialist, creative designers, programming experts, and PLUS scientific content experts. Both real-time and ecological momentary assessments were used to comprehensively capture influences on toileting decisions including perceived access to toileting, degree of busyness or stress or focus, beverage intake amount, urge degree, or a leakage event. The restriction on the maximal number of taps for any screen was six. PLUS consortium investigators did pilot-testing. Formal usability testing relied on the recruitment of community-dwelling women at four PLUS research sites. Women used the app for 2 consecutive days. Outcome measures were the system usability scale (SUS; 0-100 range) and the functional Mobile Application Rating Scale (1-5 range). These scales were embedded at the end of the app. The estimated a priori sample size needed, considering the SUS cut point score set at ≥74, was 40 women completing the study.

**Results:**

Funding was provided by the National Institute of Diabetes and Digestive and Kidney Diseases since July 2015. The integrity of the build process was documented through multiple 5-minute videos presented to PLUS Consortium and through WhereIGo screenshots of the final product. Participants included 44 women, with 41 (93%) completing data collection. Participants ranged in age from 21 to 85 years, were predominantly non-Hispanic White (n=25, 57%), college-educated (n=25, 57%), and with incomes below US $75,000 (n=27, 62%). The SUS score was 78.0 (SE 1.7), which was higher than 75% of the 500 products tested by the SUS developers. The mean functional Mobile Application Rating Scale score was 4.4 (SE 0.08). The build and informal acceptability testing were completed in 2019, enrollment for formal usability testing completed by June 2020, and analysis was completed in 2022.

**Conclusions:**

WhereIGo is a novel app with good usability for women to report toileting decisions, urination, and fluid intake. Future research using the app could test the influence of real-time factors on bladder health.

**International Registered Report Identifier (IRRID):**

RR1-10.2196/54046

## Introduction

Lower urinary tract symptoms (LUTS) are increasingly recognized as a public health concern. In community-based studies, 73% of women report at least one storage or voiding symptom, with LUTS expected to affect more than 43 million women in the United States over the next 30 years [1,2]. Despite this high prevalence, little is known about the prevention of LUTS in women.

The Prevention of Lower Urinary Tract Symptoms (PLUS) research consortium aims to identify influences ranging from individual biological to broad ecological factors (eg, environmental, sociocultural, and physical factors) that promote bladder health and prevent the onset of LUTS [3]. The PLUS definition of bladder health is “a complete state of physical, mental, and social well-being related to bladder function and not merely the absence of LUTS. Healthy bladder function permits daily activities, adapts to short-term physical or environmental stressors, and allows optimal well-being (eg, travel, exercise, social, occupational, or other activities” (page 978) [4].

Daily repeated decisions about toileting may affect the risk of developing LUTS, therefore, the PLUS Consortium has prioritized the study of toileting behaviors and their potential impact on bladder health. We aimed to minimize recall bias, favoring real-time reporting to the extent possible. A mobile phone app offered an ideal mode for this type of data collection.

The PLUS team hypothesized that a woman’s location greatly influences toileting decisions and may also limit voiding in a specific environment. Thus, we named the proposed mobile app product “Where I Go” (abbreviated in the body text as WhereIGo). The intent was a user-friendly experience with an app that is self-explanatory, has visual appeal, and makes appropriate use of humor, while also providing ease in recording toileting decisions with no more than 6 taps at any reporting interaction done either in real-time or within a 3-4 hour recall period of the toileting event. We report here details of the conceptual development, construction, and usability testing of WhereIGo.

## Methods

### Overview

Multimedia Appendix 1 offers a road map to WhereIGo via a screen-based slide deck. This pictorial deck shows each screen of the app post–item development and post–complete build, just prior to the formal usability testing reported in this paper.

### Developing Items for WhereIGo

In the early phase of development for the app, the PLUS research consortium brainstormed candidate items for inclusion in the app, followed by a survey to prioritize and then reduce items. Forefront in priority was the location of toileting events (where) and its situational influence on decisions women must make about when to toilet. This priority is consistent with the socioecological conceptual model used by the consortium [5].

The candidate items resulting from this process were organized in a large Excel (Microsoft Corporation) spreadsheet with rows that provided category headings in the cell above candidate items for the category beneath it. Example categories (in italics) and candidate items are *Onboarding activities* (eg, baseline information, pinning “home” location, and sleep time), *Captures* (time of pee, location of pee), *How strong was urge* (slider indicator), *Why went* (eg, no time to go later), *Barriers or delay reasons* (eg, work restrictions), *Bathroom quality* (eg, long wait), and *Ecological momentary assessment* (eg, beverage intake and urine leak tracker over last 3-4 hours). Additional columns showed the movement of items falling out over time as original candidate items conceptualized were reduced from 105 to 73 using an iterative process within the build team.

### Constructing WhereIGo

Information demonstrating the item reduction process, transdisciplinary team build processes, and technical approaches to constructing the app are provided in Multimedia Appendices 2-6. Multimedia Appendix 2 is a 5-minute video that illustrates item candidate inclusion or exclusion tables and Multimedia Appendices 3-6 reveal the build team’s processes.

In brief, WhereIGo was built at the University of Michigan’s Center for Health Communications Research (UM invention registration OTT reference 2019-292) using Cordova (Apache) dual-platform software (simultaneous build for both iOS and Android). A guiding principle in the build was that any interaction asked for by the app would require no more than 6 clicks by the user.

**Figure 1:**
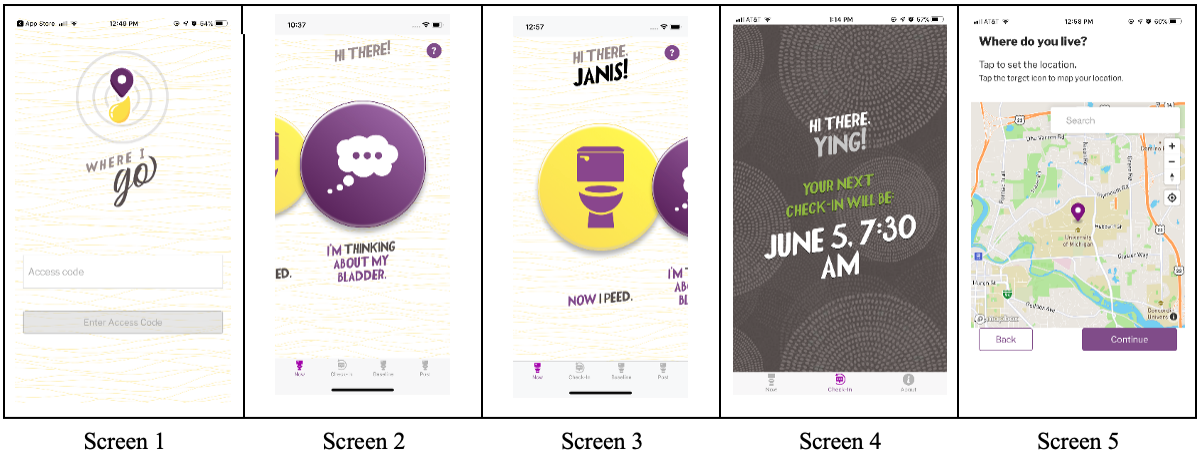
Visual representation of selected key screenshots of WhereIGo, an app designed to collect data on toileting decisions made by community-dwelling women in real time wherever they are. Each of the 5 screens shown here leads to additional data collection screens under these lead messages: screen 1 logo, screens 2 and 3 real-time tap me components (“I’m thinking about my bladder” or “Now I peed”), screen 4 notification as heads-up for an upcoming look-back check-in (reflecting on the past 3-4 hours), screen 5 pin drop locator of common locations (home location pin required and opportunity to pin and name other locations as clarity of meaning for geocoding). Short look-back periods called “check-ins” were also offered with 3-5 push notifications per day that take the user to the menu. These prompts were to ascertain difficulty on the part of the participant in self-initiating reports on the main variable of interest—a toileting event. Screen 4 shows when the user’s next check-in time will be as a form of anticipatory guidance using the push notification. Screen 5 shows the “pin-drop” feature used to identify a user’s “home” location and optionally label other common locations such as “work.” Screenshots of the entire WhereIGo app (its 72 “screen-pages” and an additional 15 exit survey items) are available in Multimedia Appendix 1.

The main variable of interest is a toileting event. Figure 1 portrays a brief overview of the screen visuals that guide women through WhereIGo. Each panel shows a key component within the app. Screen 1 highlights the logo and other design features. Screen 2 “thinking about my bladder” represents a real-time reporting option, which when tapped brings up a short survey on decisions made around that toileting event. Screen 3 “I just peed” similarly can be tapped in real time and offers reporting details.

WhereIGo does not constantly track location. Rather, it has a built-in feature of automatically capturing latitude and longitude coordinates only when triggered by a user interaction with the app. These coordinates allow for estimates of whether the user’s location at the time of interaction was home or elsewhere.

WhereIGo captures toileting decisions under two real-time modes: “I’m thinking about my bladder” and “Now I peed.” Tapping on either one of these real-time prompts triggers follow-up survey questions. When “I’m thinking about my bladder” is tapped, a slider scale for reporting the degree of urge appears. This urge-o-meter slider labeled “How strong is your urge to pee?” is anchored at either end as “No Urge” and “Strong,” with a facial expression emoticon that changes to more concern as the marker slides toward the “Strong” end. Once the urge-o-meter has been slid, a screen follows: “When are you planning to pee?” with response categories “now” or “later.” If “later” is chosen, a survey question appears: “Why aren’t you going to pee now?” along with a previously determined list of responses or a chance to “Type your own reason.” The entirety of this interaction requires only 4 taps but addresses the biological urge sensation variable and the socioecological factors for delaying voiding (eg, “No place to pee,” “Long line for toilet”). The interaction is time and date stamped and geocoded by latitude and longitude.

When “I just peed” is tapped, a screen follows: “When did you pee?” with response categories “Now,” “10,” “20,” or “30+ minutes ago.” Then these response options appear, “Where did you pee?” response options including many preset options such as “Home,” “Workplace,” “Restaurant,” “School,” or a “New location” option. The urge-o-meter screen appears again to measure the urge at the time of toileting. Then the screen switches to “Why did you pee?” and “Check all that apply” with 5 preset response options and an opportunity to “Type your own reason.” Finally, “If you had to wait before you peed, why did you wait?” with similar choices including an option to type your own reason. The entirety of this interaction requires only 6 taps (unless multiple options are selected).

In addition to the real-time modes, the app provides an opportunity to record missed events through “check-in” opportunities instigated by push notifications approximately every 3-4 waking hours. Times of check-ins are coded into the app and are individualized to the person’s reported usual wake-up time and reported usual bedtime, divided evenly for push notifications across the awake time interval, but no more than 4 times per day and no less than 3. At the first morning check-in, the participant is asked to reflect on total sleep hours and to add any additional voids not recorded in real-time during the night.

Additionally, users are asked at check-in to report on items such as mindset (slider anchored by “Calm” and “Stressed” as the end points), beverage intake, “What strategies did you use in the last few hours to manage when you go pee?” and any urine leakage. At the end of each check-in, a cartoon joke appears as a fun reward.

### Usability Testing Protocol

The research protocol for usability testing was fully approved by the external expert panel for the PLUS Consortium. Multimedia Appendix 7 provides the full protocol for usability testing. We purposely excluded the University of Michigan PLUS Research Center in formal usability study data collection to remove potential bias since this team did the build and iterative informal process of debugging checks. Refer to Multimedia Appendices 3-6 for additional details of work performed at the University of Michigan.

### Ethical Considerations

The University of Michigan served as the oversight PLUS research center for building and testing WhereIGo. Institutional review board (IRB) approval (HUM00162517) was given on October 8, 2019. The additional 4 PLUS research centers, designated for recruitment and data collection activities, also gained approval per sites and IRB# as follows: Washington University in St. Louis (201912088), Loyola University Medical Center (LU212615), University of Pennsylvania (833718), and University of Alabama at Birmingham (300003879). Participants were asked to consent to 2 in-person study visits. The first study visit was described in the informed consent document as involving 40-60 minutes for filling out paper surveys including questions about the make and model of the participant’s smartphone. Participants were primed about receiving a guide at visit 1 showing how to connect to the internet and download WhereIGo. Each participant was informed that visit 1 would involve opening the app, picking a nickname to use in it, responding to the app demographic questions survey within it, and dropping a map location pin for “home”. Optionally, participants could drop up to 4 pins for places frequented routinely outside the home (eg, work, school, and gym). The general description for the consent of the 48-hour use of WhereIGo was described as being asked to (1) download a mobile phone app onto a personal smartphone, (2) self-initiate interactions with the app starting the next morning and for 48 hours following, (3) leave home at least once during the 48 hours’ use, and (4) receive prompts by notifications 5 times a day (for responding to look-back questions about the prior 3-4 hours). The look-back questions consent descriptions included the opportunity to log missed pee events, any urine leakage, degree of felt stress or busyness, and volume or type of beverage intake. Participants were informed of additional prompted interaction with WhereIGo at the end of 48 hours of use to answer a survey that included 17 multiple-choice questions and 1 text response question about personal experience using the app. Consenting to the second study visit involved an agreement to open WhereIGo during the visit to make sure the final survey was completed, and answer 15-20 questions about having used the app and how it could be improved. Participants consented to (optionally) receive assistance in removing the app. Inconveniences of taking part in the study were made known including the potential for embarrassment and the risk for potential loss of confidentiality. The consent form provided information about the assignment of a unique identification number, storage in a password-protected file or locked research office, and contact information for research staff was provided for future questions. Participants were informed that only a single document linking the names and study code IDs would be maintained and would be password-protected or locked in an office accessible only to study members. Geolocation data would be kept private, specifically that the geocode data would be kept on a secure driver, again only accessible by key members of the research team. At no time would other parties, such as Apple or Google, have access to data on this secure app. Participants consented to compensation of US $25 for completion of the first in-person visit, US $75 for using WhereIGo over the next 2 days, and US $50 for the second in-person visit, totaling US $150 for completing the study.

### Study Design

A cross-sectional study design was used with sampling from regions surrounding the 4 PLUS research centers listed above.

### Sample and Recruitment

Recruitment strategies included the distribution of flyers, advertisements in local community centers, contacting participants from previous studies, social media, and word-of-mouth. The recruitment aim focused on adult women who were living in community settings.

For eligibility screening, inclusion criteria were (1) women 18 years and older; (2) fluent in written and spoken English; (3) able to stand and walk independently without human assistance (use of cane or walker allowed); (4) own a smartphone (Android or iOS) on which calls can be made; (5) have downloaded at least one app in the past 6 months; (6) willing to respond to WhereIGo prompts or texts and input data about toileting behaviors for a consecutive 48 hours, during which they will leave their home at least once, but not change time zones; and (7) agree to 2 in-person visits at the study site. Exclusion criteria were (1) having a physical or mental condition that would prevent completing written questionnaires and interactions with the app, (2) institutional living arrangements (ie, skilled nursing, long-term care, or rehabilitation center), (3) currently pregnant, (4) diagnosed with a neurogenic or congenital bladder condition, and (5) unable to use the toilet independently.

### Procedures

Eligible women were sent study information and an IRB-approved consent form and were scheduled for an in-person visit. At this first visit, women were asked to download and install WhereIGo onto their smartphones. Women were requested to begin use of the app anytime they wished after download, and the formal data collection would start with a check-in notification they would receive the next morning, shortly after the “wake time” indicated individually at download. Starting with this “wake time,” 48 consecutive hours of app use was expected. Participants received a final check-in notification that included a request to fill in usability surveys that were embedded within the app (approximately 20 questions).

### Outcome Measures

The primary outcome measure was the system usability scale (SUS) [4]. SUS evaluates users’ satisfaction based on ease of use. It is a validated self-administered questionnaire widely used in the evaluation of technology products [5], with a Cronbach α coefficient of 0.91. It consists of 10 items that are rated on a 5-point Likert scale ranging from strongly disagree (0) to strongly agree (4). Responses to each item are summed and multiplied by 2.5 to convert scores to a scale of 0 to 100 and then normalized to a percentile. SUS scores above 68 are considered above average [6]. A secondary outcome, the Mobile App Rating Scale, is a simple, objective, and reliable instrument with 5 subscales for classifying and assessing the quality of mobile health apps [7]. We used the function subscale (fMARS), which consists of 4 items assessing an app’s performance, ease of use, navigation, and gestural design. Each item is rated using a 5-point Likert scale (1=inadequate, 2=poor, 3=acceptable, 4= good, and 5=excellent). An overall function score was calculated based on the mean score across items. This subscale has excellent internal consistency (Cronbach α=0.80) and moderate interrater reliability (interclass correlation coefficient=0.50) [7].

### Statistical Analysis

We hypothesized cut point indicators for acceptability usability to include a SUS score above 74 [6] and a mean fMARS score of >3.5. Allowing for a 5% drop out, we estimated a minimal sample size of 40 participants to achieve sufficient precision for estimating the SUS score (ie, within one-third of the SE from the mean to the confidence limits). Per SUS developers, a SUS score of 74 has higher perceived usability than 70% of all products tested [6].

## Results

Funding for the PLUS consortium was provided by the National Institute of Diabetes and Digestive and Kidney Diseases of the US National Institutes of Health. Funding began in July 2015 and continues through July 2025. Recruitment for this project began in September 2019 with completion of enrollment in June 2020.

A total of 44 community-dwelling women were enrolled. Participants were aged on average 44 (SD 18.2) years, and 39% (n=16) of participants identified as a racial minority, predominantly Black (Table 1). Within this sample, iOS phones were used by 75% (n=33) of participants, Android by 23% (n=10) of participants, and one phone type was missing.

A total of 41 (93%) women completed the SUS and fMARS usability surveys. The average score for the SUS was 78.0 (SE 1.7), statistically significantly higher than the minimal cut point of 74 established a priori as acceptable usability. The score for the fMARS was 4.4 (SE 0.08), also statistically significantly higher than the a priori established cut point of 3.5.

During the second visit, the overall feedback was very positive with no qualitative findings leading to additional changes in the app build. A single exception was a clear desire for feedback to be provided to the user about their bladder health habits, with many participants asking for this feature to be added in the future.

With these acceptable usability findings, the research team established timelines for the next steps. These are (1) 2023-2024 analyzing and publishing additional findings from these same women’s data, which was collected across the full 48 hours of WhereIGo implementation, and (2) 2024-2025 launching, analyzing, and publishing the first population-based study to use WhereIGo per the PLUS consortium’s ongoing National Institutes of Health-funded parent study called Rise for Health. Rise for Health is a study of women’s bladder health conducted by research teams from 10 universities across the United States. The WhereIGo protocol published here will be an addition to that ongoing research, with WhereIGo data collection beginning midyear 2024.

**Table 1:** Demographic information as collected within the WhereIGo app.

Characteristic		Community-dwelling women (N=44)
**Age (years)**		
	Mean (SD)	44.3 (18.2)
	Range	21-85
	18-25, n (%)	8 (18)
	26-45, n (%)	16 (36)
	46-65, n (%)	15 (34)
	≥65, n (%)	5 (11)
**Race or ethnicity (check all that apply), n (%)**		
	American Indian or Alaska Native	1 (2)
	Asian	4 (9)
	Black or African American	12 (27)
	Hispanic or Latino	8 (18)
	Middle Eastern or North African	1 (2)
	Native Hawaiian or other Pacific Islander	0 (0)
	White	25 (57)
	Other race or ethnicity or origin	1 (2)
	Participant did not answer	3 (7)
**Income (US $), n (%)**		
	<$25,000	9 (20)
	$25,000-$49,999	9 (20)
	$50,000-$99,999	15 (34)
	>$100,000	9 (20)
	Missing	2 (4)
**Education, n (%)**		
	High school diploma, GED^a^, or less	0 (0)
	Some college	12 (27)
	Associate or bachelor’s degree	18 (41)
	Master’s degree	11 (25)
	Professional or doctorate degree	3 (7)
**Employment (check all that apply), n (%)**		
	Homemaker	5 (11)
	Student	8 (18)
	Not working or unable to work	8 (18)
	Working one or more jobs	30 (68)
**Language (check all that apply), n (%)**		
	English	43 (98)
	Spanish	3 (7)
	Other	2 (4)
**Sought care for bladder from health care provider, n (%)**		
	Sought care	9 (20)
	Did not seek care	34 (77)
	Missing	1 (2)
**Number of toilets in home, n (%)**		
	1	12 (27)
	2	23 (52)
	3	9 (20)
**Phone type, n (%)**		
	Apple	33 (75)
	Android	10 (23)
	Missing	1 (2)

^a^GED: General Educational Development.

## Discussion

### Principal Findings

In this report, we describe the development and usability results of a novel mobile app called WhereIGo, which collects real-time information on women’s toileting decisions and behaviors. We tested the app in a variety of locations across the United States, showing well above-average user satisfaction across different geographic locations.

With this description of WhereIGo usability testing, we now embark on crucial validity testing. We are addressing certain minor limitations (“bugs”) that arose from older phone models and from the Cordova dual-platform software, both of which are largely historical problems. The Cordova dual-platform software (novel at the time in incorporating Android and iOS in a single build) has ceded to newer dual-platform software with enhanced functionalities.

### Limitations

Despite the participant’s desire for feedback, currently, WhereIGo does not yet provide feedback data to the user but it remains a goal to incorporate in future versions. Of note, this was a highly educated sample of only English speakers. Future usability testing should include those with lower literacy. It is also acknowledged that all participants had resources to secure a cell phone for the app use, affecting the generalizability of results in future studies.

### Conclusions

WhereIGo is a novel mobile app designed to collect data on women’s choices of when and where to toilet. Women report above-average usability, good functionality, and high appeal. Our future goals are to further refine and enhance WhereIGo as informed by validity testing, participant feedback, and newer cutting-edge dual-platform software. Ultimately, the plan is to use WhereIGo in studies of lower urinary tract function to provide women with real-time reporting and to capture behaviors and influences on toileting decisions that may ultimately represent modifiable risk and protective factors for bladder health.

## Appendix

Multimedia Appendix 1 Screenshots of the completed WhereIGo mobile app.

Multimedia Appendix 2 WhereIGo App candidate variables by author JMM. This 5-minute video features a spreadsheet compilation of candidate items for potential inclusion in a new data collection tool for influences on women’s toileting decisions. The list, evolved out of a brainstorming session conducted by the National Institutes of Health Preventing Lower Urinary tract Symptoms (PLUS) research consortium, was foundational to the WhereIGo build process. The video includes initial candidate items that did or did not make it into the final version of the application.

Multimedia Appendix 3 Part 1: creative design process of WhereIGo by artist and designer Ian Moore. This 5-minute video reveals the artist’s process behind the many subtle and beautiful features in WhereIGo, including how careful imagery creation results in an inviting app.

Multimedia Appendix 4 Part 2: user interaction design of WhereIGo by Ian Moore. This 5-minute video reveals more about how a designer builds code for the app to support choices on factors such as colors and movement (eg, “a sense of wiggling”) for selected screen visuals.

Multimedia Appendix 5 Role of behavioral science in building WhereIGo by plain language specialist Caroline Carter. This 5-minute video demonstrates how to achieve clear, concise, organized, and appropriate in-app text to maximize a satisfying experience for WhereIGo users.

Multimedia Appendix 6 Behind the scenes tech team by Mike Nowak and Shelly Chang: This 5-minute video is presented as a conversation between two coding experts, who work behind the scenes, to actualize choice of platform for the build and the mechanics of coding that make the entire app viable. The two contributors role-play the use of WhereIGo, so viewers can see the app in action.

Multimedia Appendix 7 Protocol for WhereIGo formal usability testing, as used by the four PLUS consortium research sites who recruited community women to use WhereIGo for 2 days and provide usability feedback.

Multimedia Appendix 8 Peer-reviewer report from the National Institute of Diabetes and Digestive and Kidney Diseases Special Emphasis Panel - National Institute of Diabetes and Digestive and Kidney Diseases (NIDDK) - Women's Bladder Health applications PLUS (National Institutes of Health, USA).

## Abbreviations

fMARS: functional Mobile Application Rating Scale
IRB: institutional review board
LUTS: lower urinary tract symptoms
PLUS: Prevention of Lower Urinary Tract Symptoms
SUS: system usability scale

## Data Availability

The data sets generated or analyzed during this study are not publicly available until the final archiving of Prevention of Lower Urinary Tract Symptoms Consortium data in a National Institutes of Health repository at the end of the grant. However, on reasonable request, the data can be made available from the corresponding author before that time.
