# Supplementary material for: Design of a Tool Capable of Assessing Environmental Sociocultural Physical Factors Influencing Women’s Decisions on When and Where to Toilet Within Real-World Settings: Protocol for the Build and Usability Testing of a Mobile App for Use by Community-Dwelling Women
Source: JMIR Res Protoc. 2024 Sep 18;13:e54046. doi: 10.2196/54046 (PMC11447419; doi:10.2196/54046)

## Slide 1
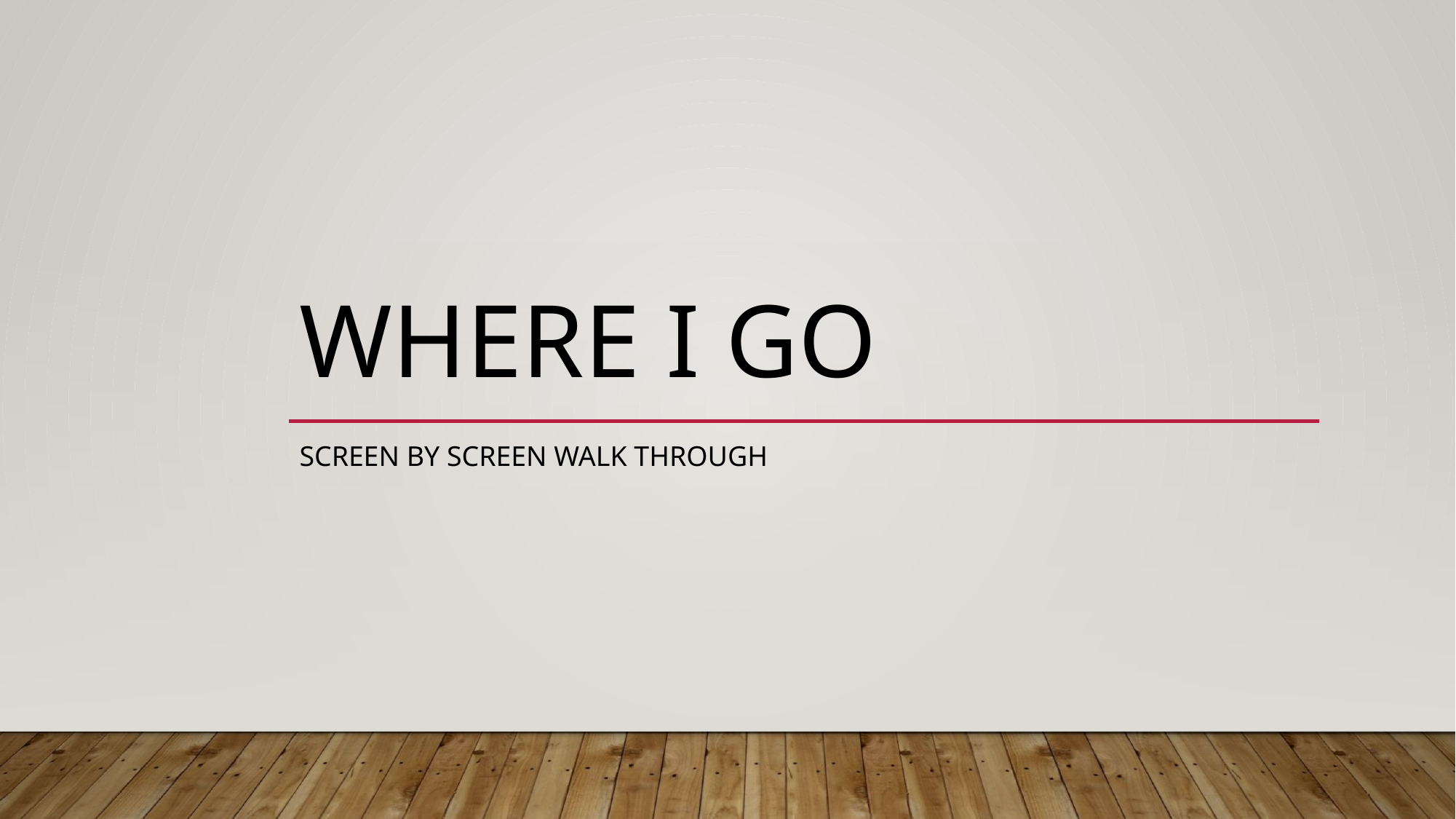

# Where I Go
Screen by Screen walk through

## Slide 2
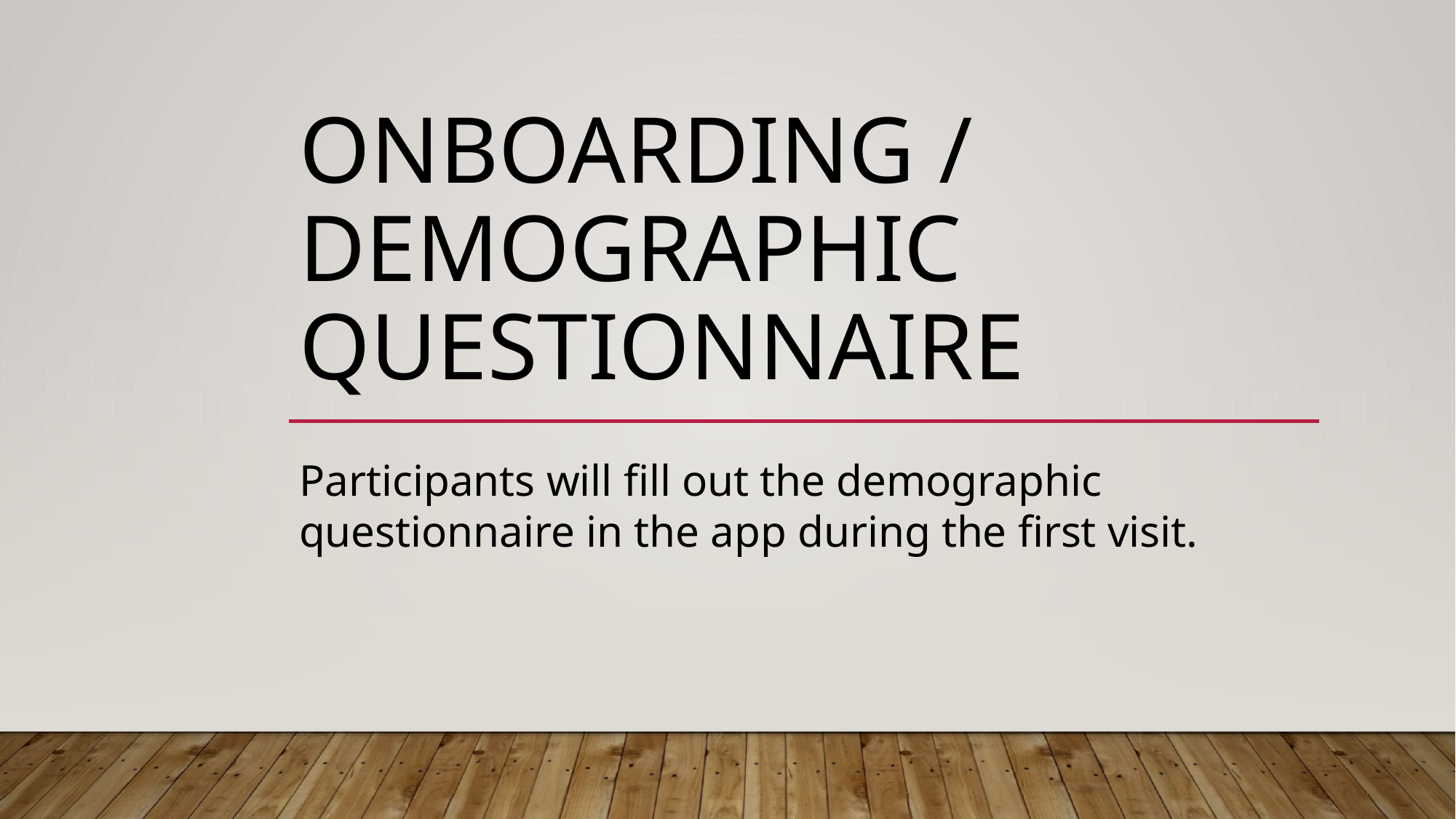

# Onboarding / DEMOGRAPHIC QUESTIONNAIRE
Participants will fill out the demographic questionnaire in the app during the first visit.

## Slide 3
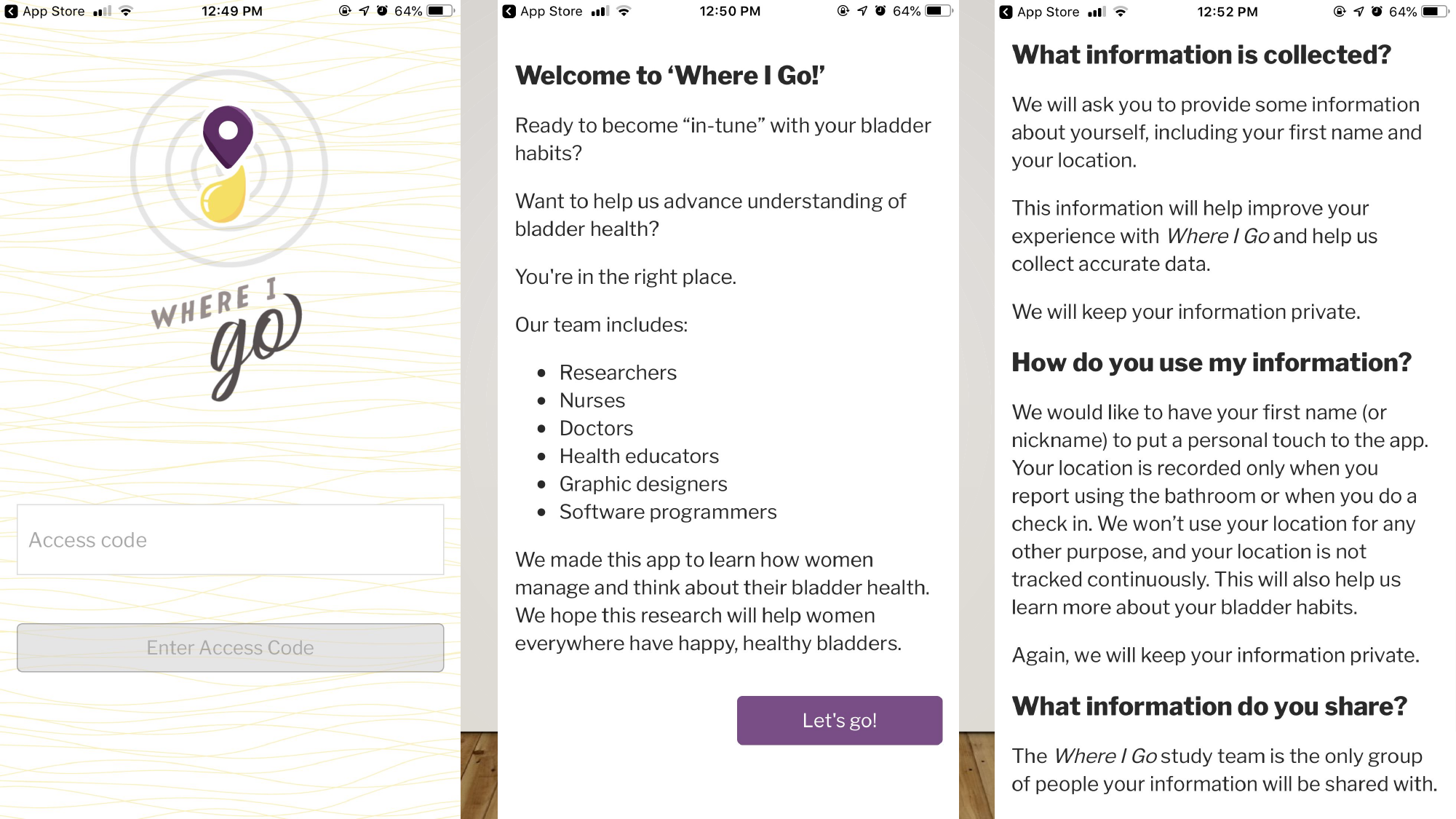

## Slide 4
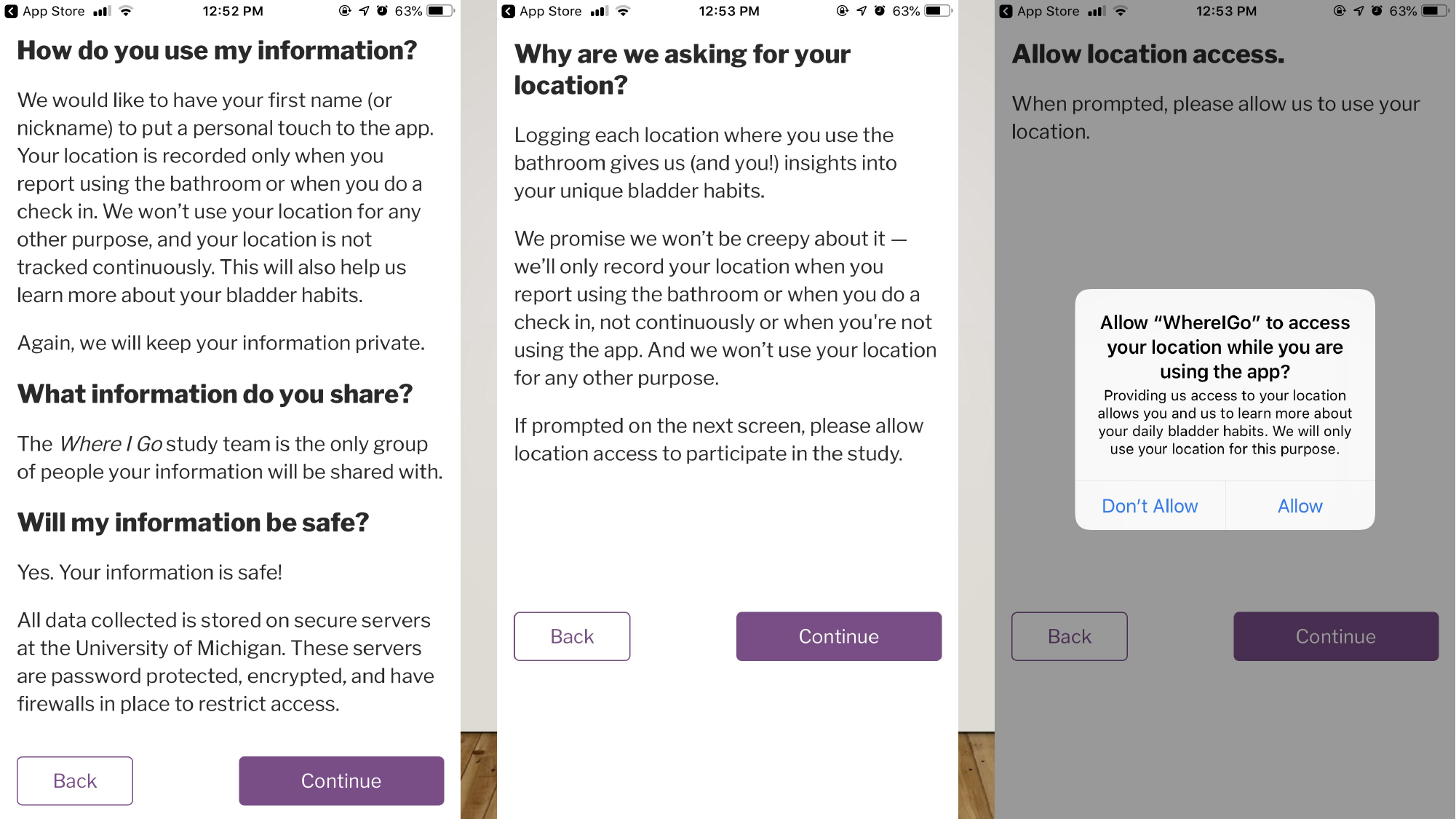

## Slide 5
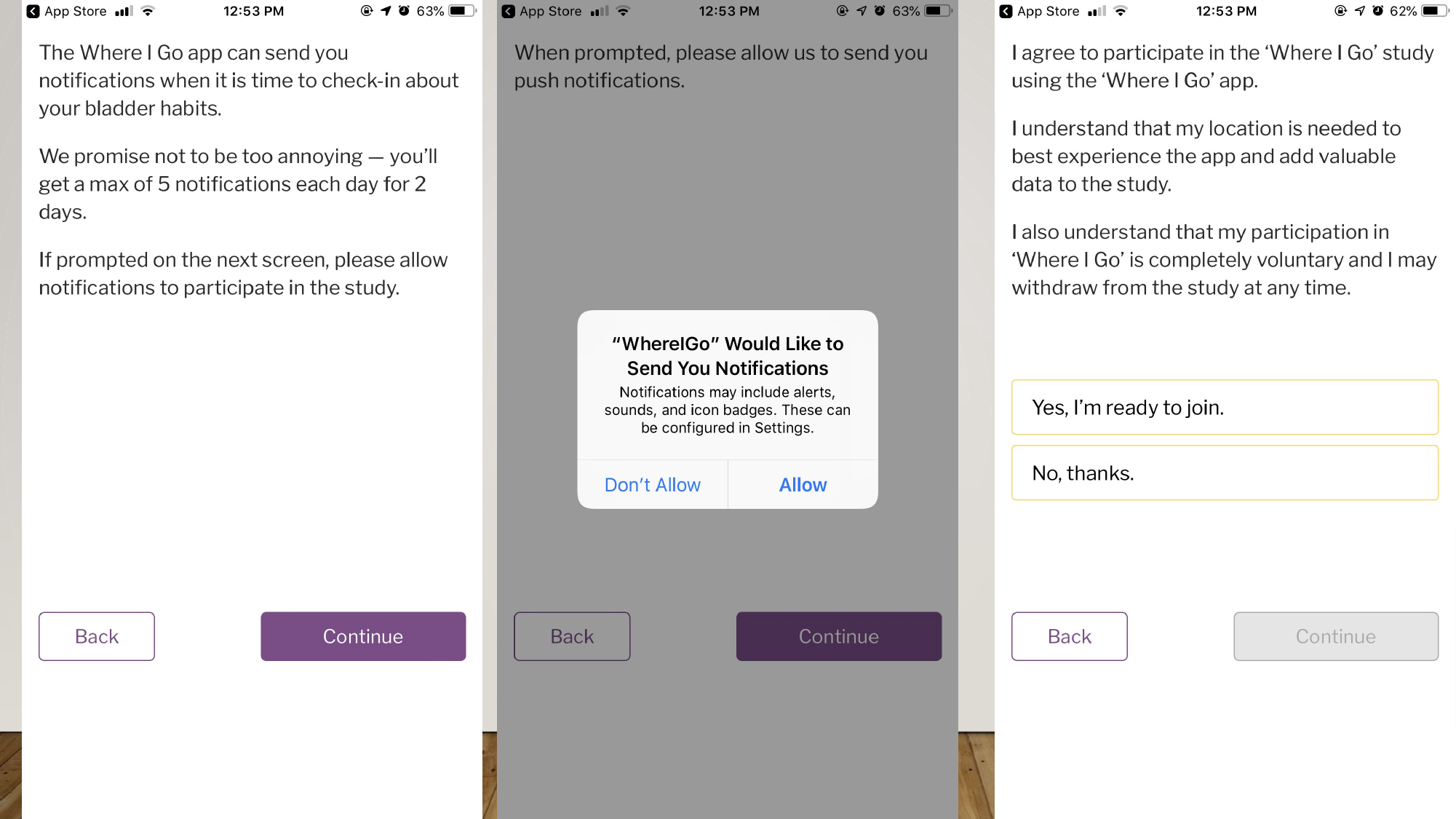

## Slide 6
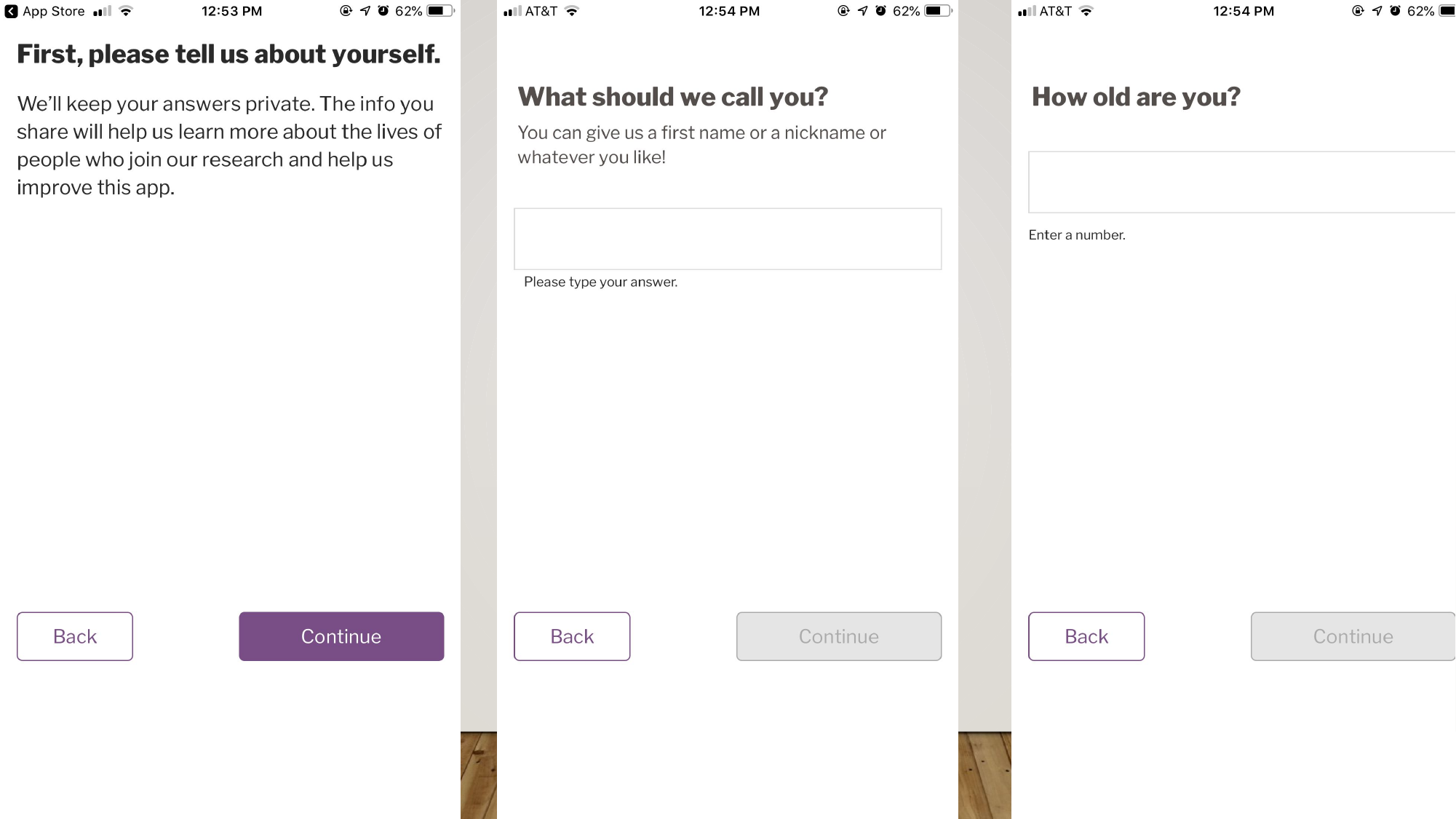

## Slide 7
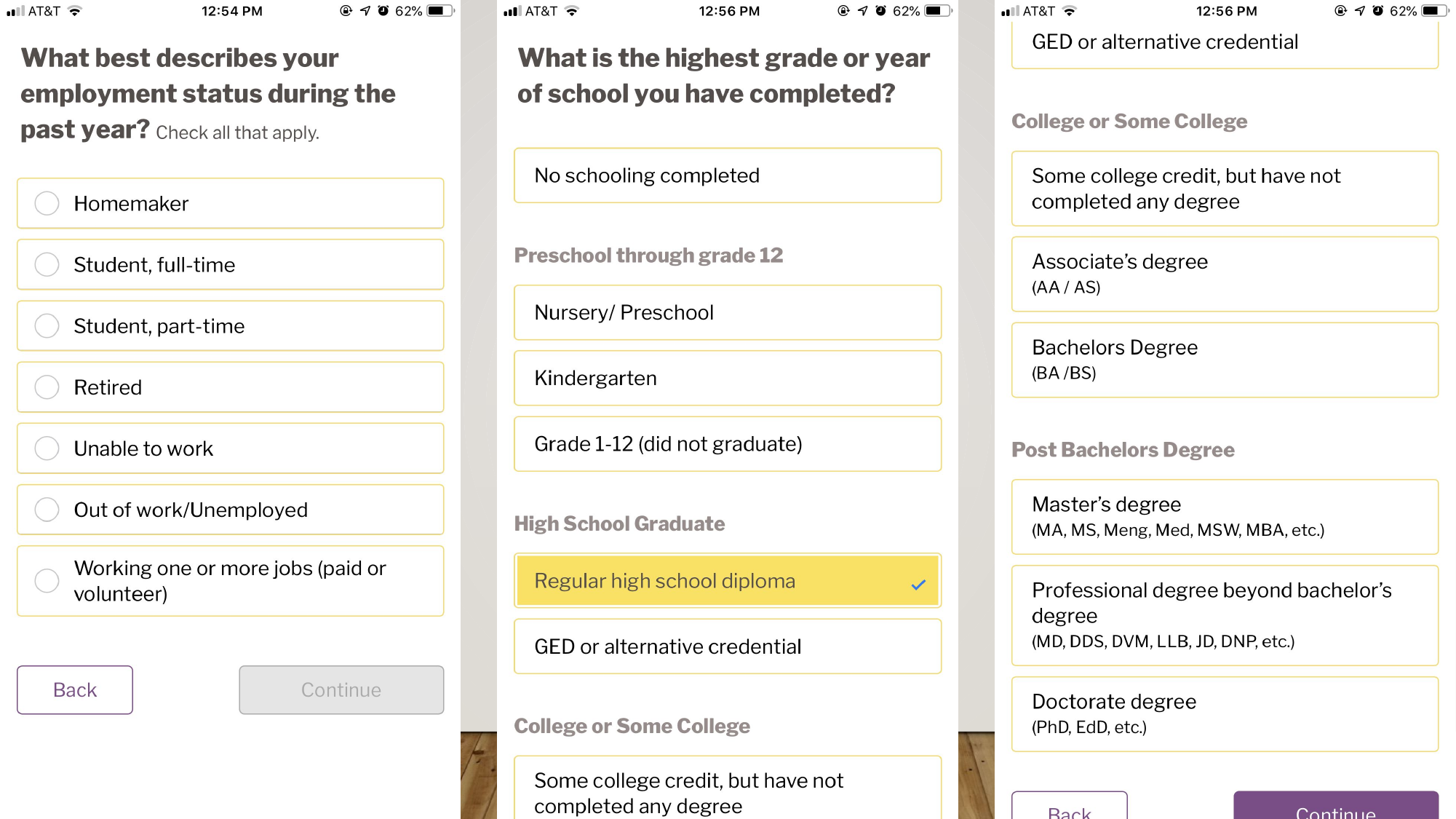

## Slide 8
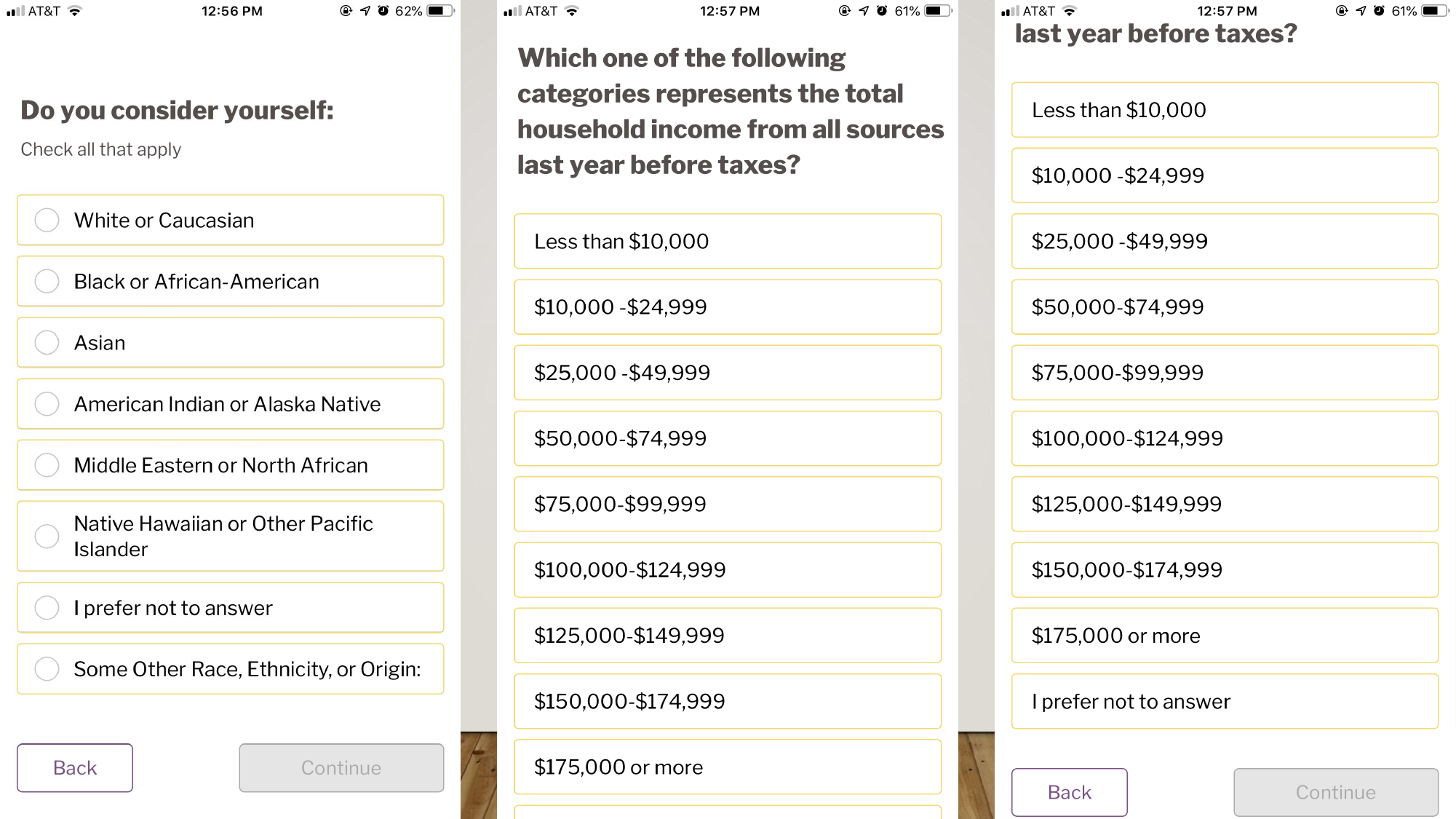

## Slide 9
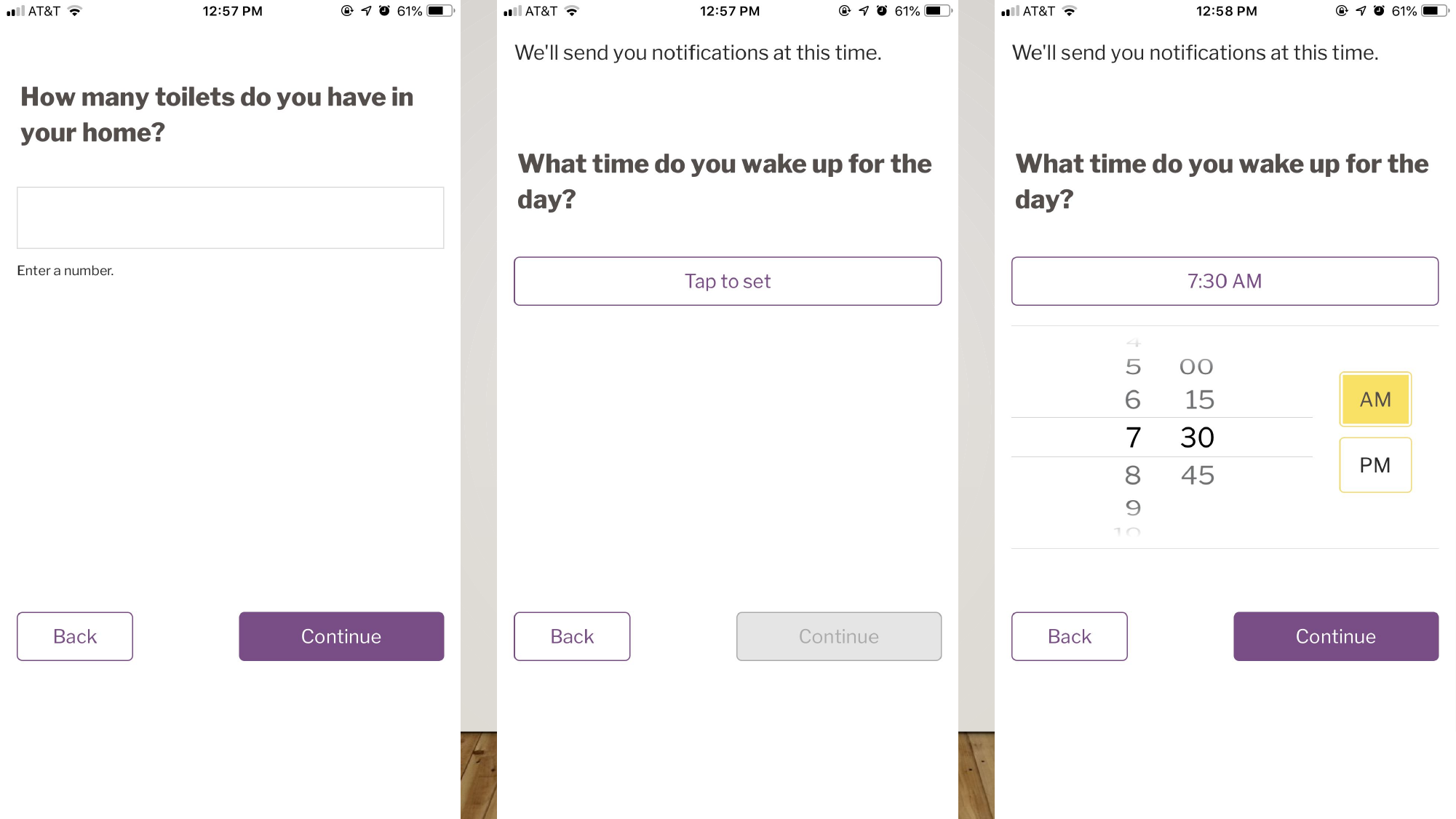

## Slide 10
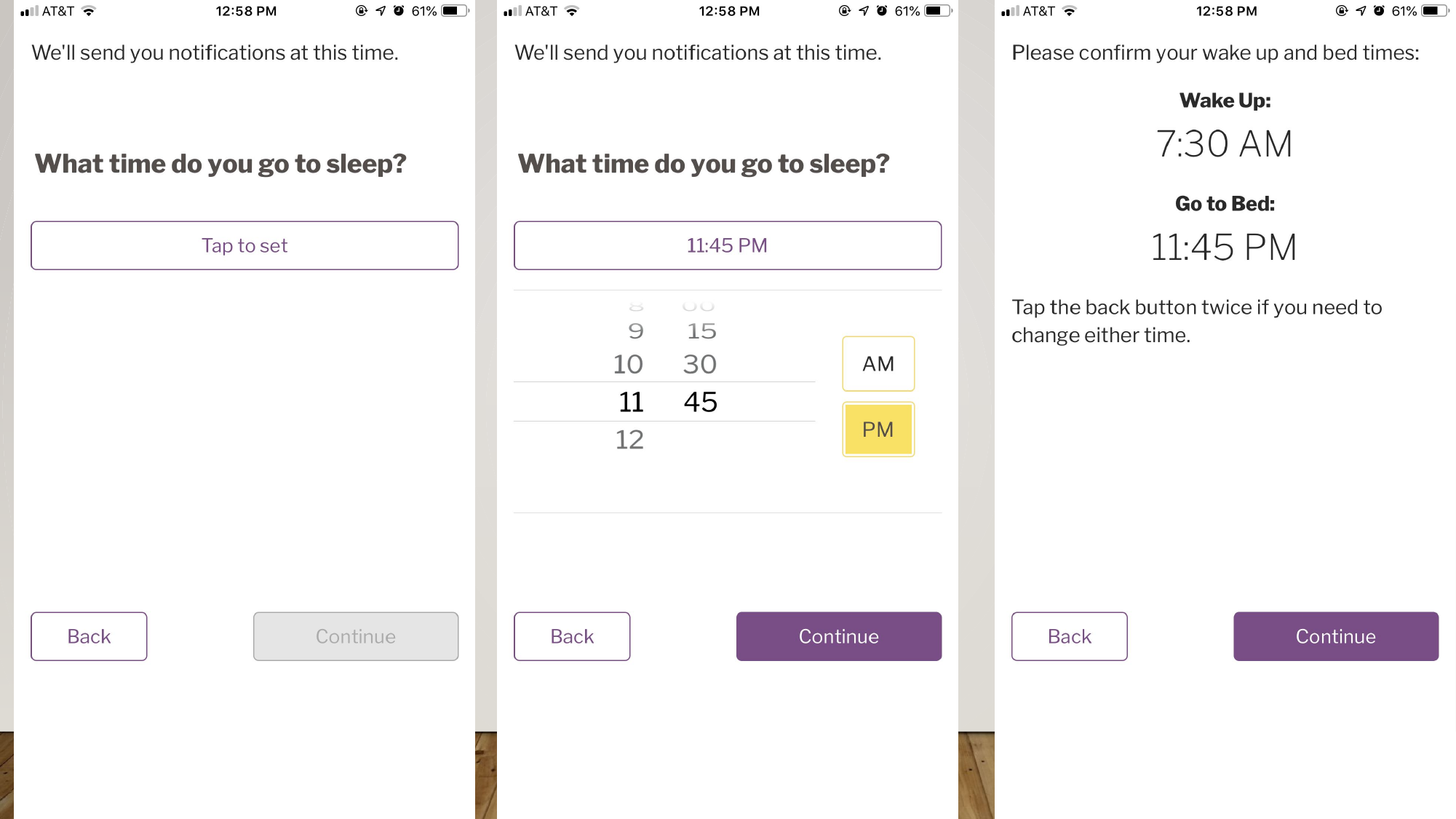

## Slide 11
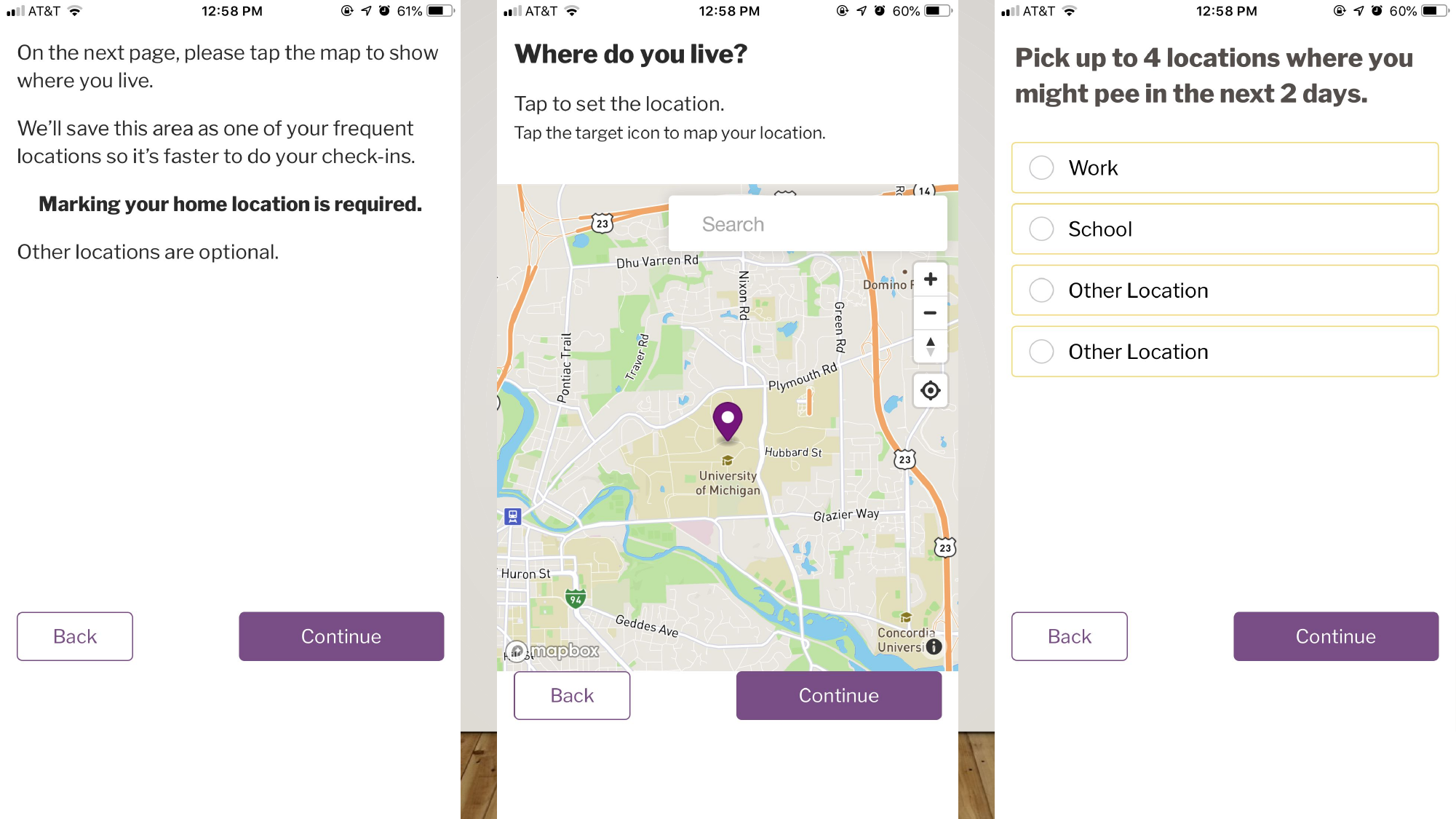

## Slide 12
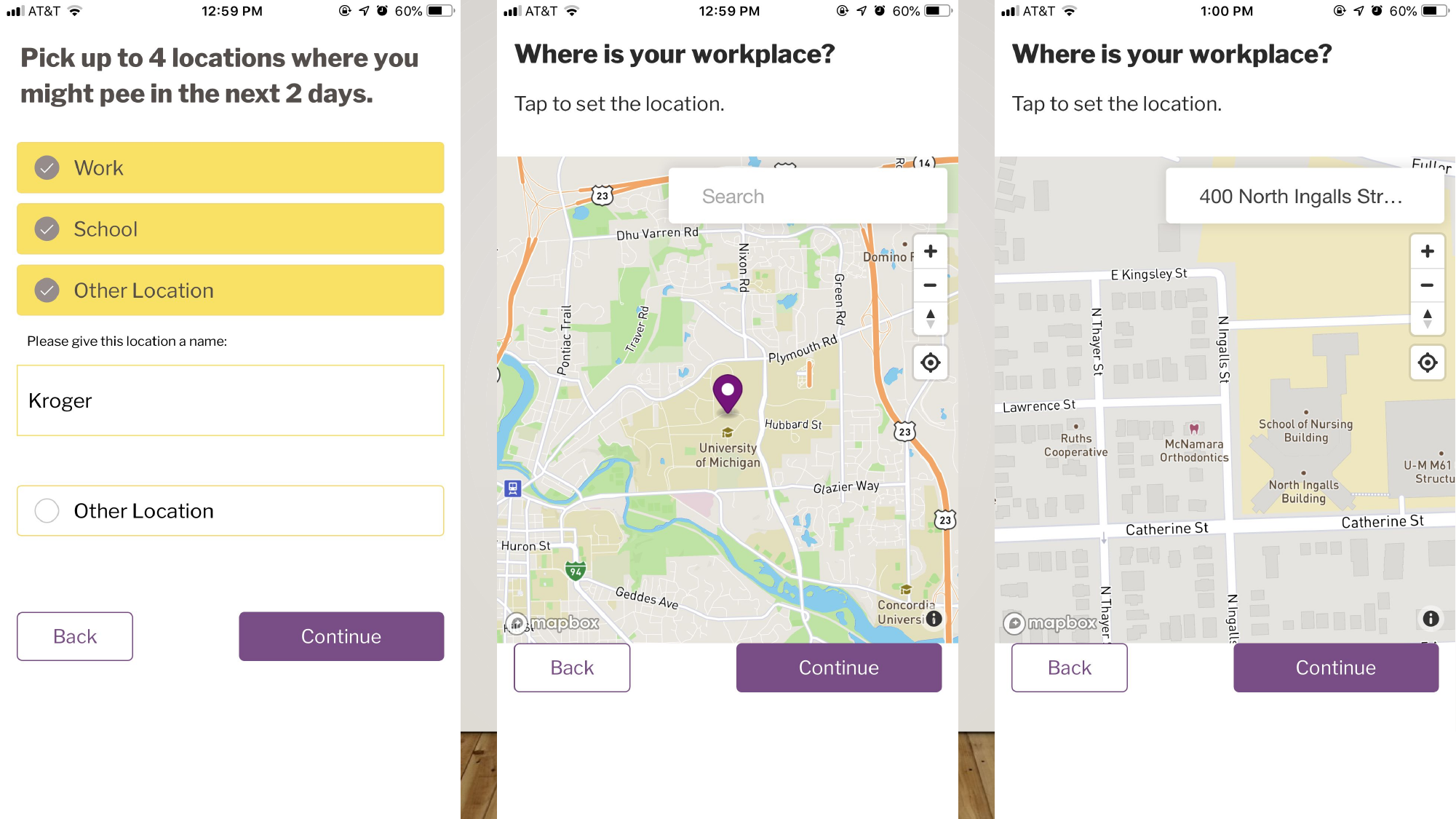

## Slide 13
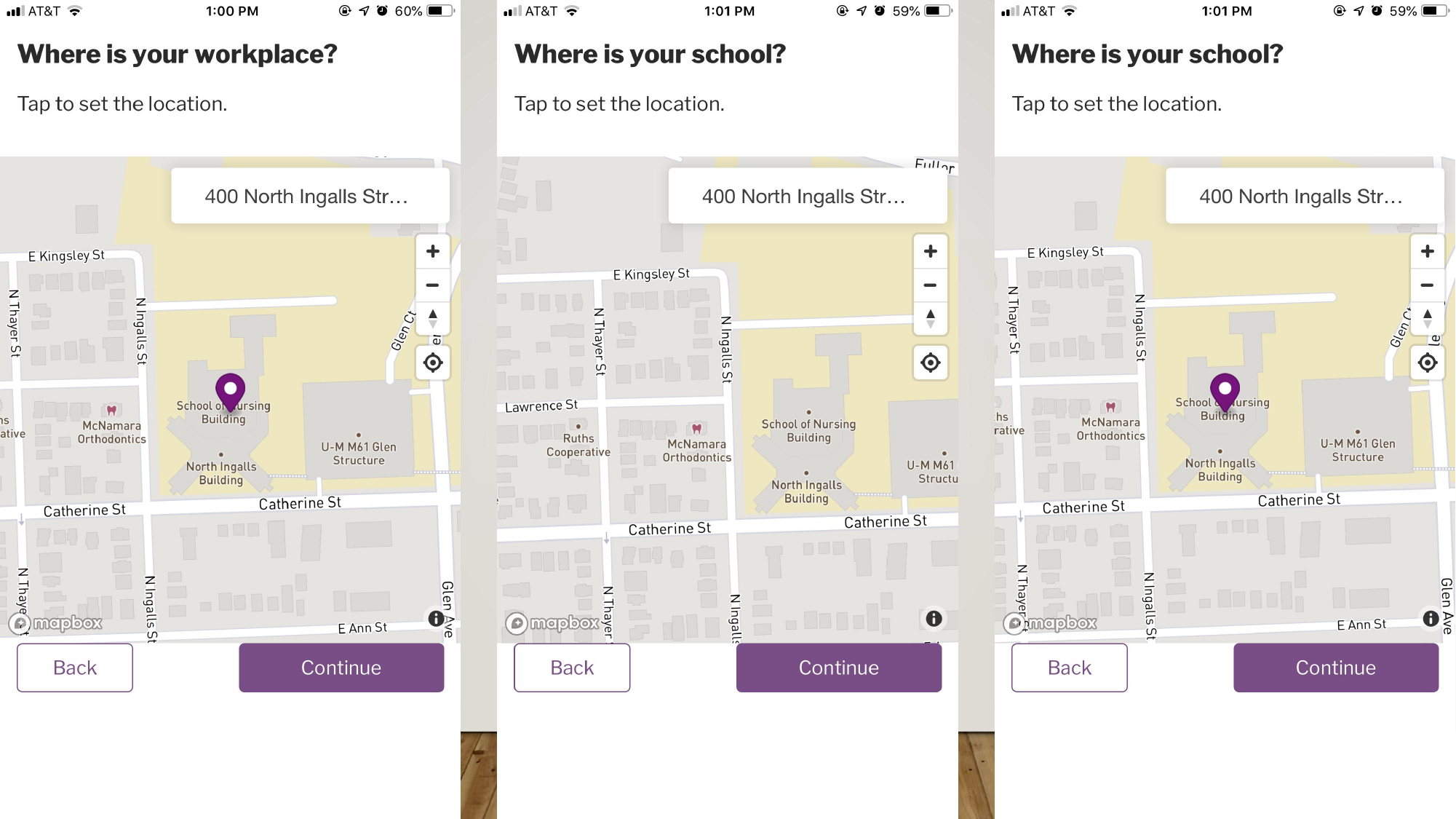

## Slide 14
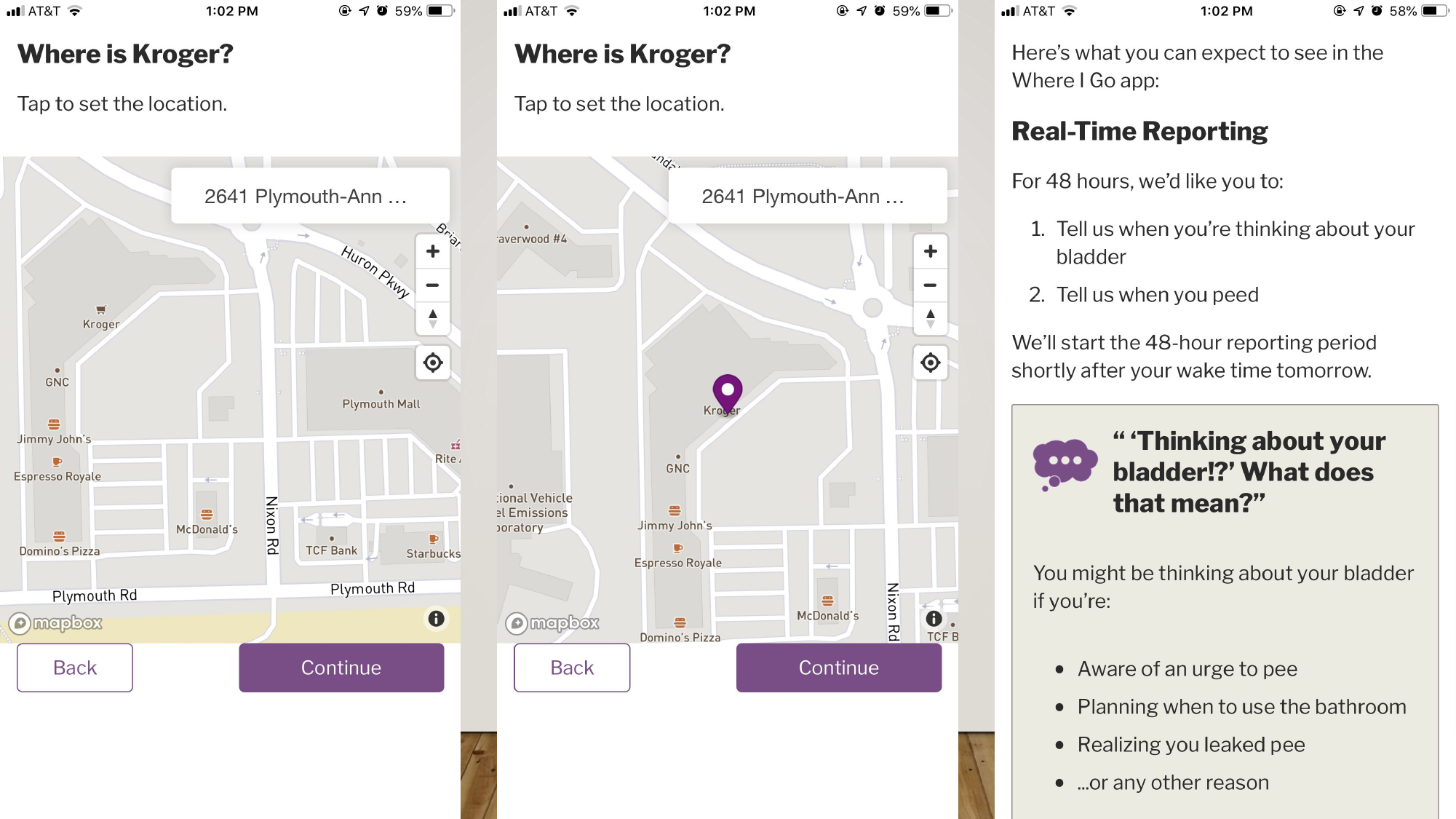

## Slide 15
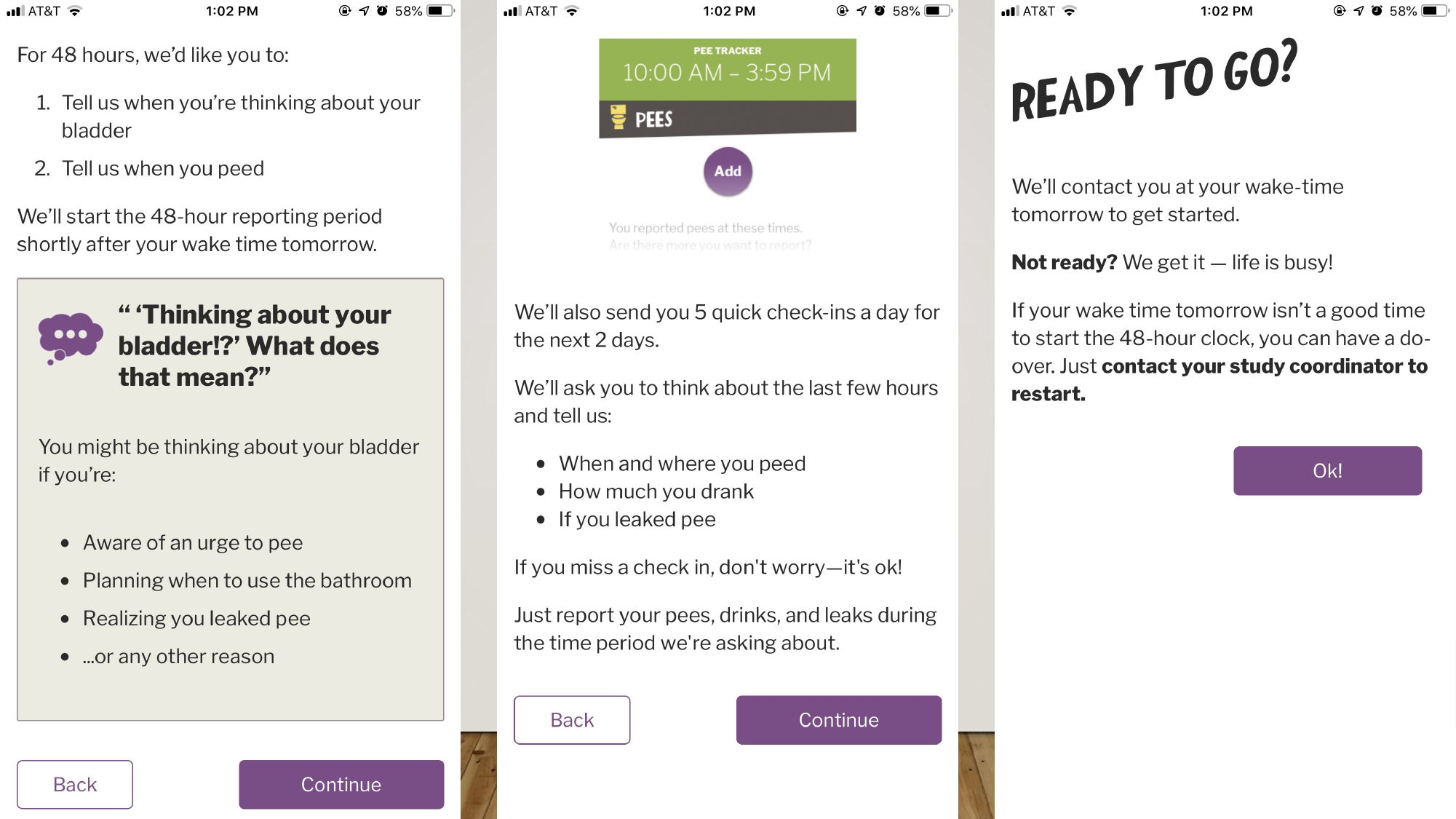

## Slide 16
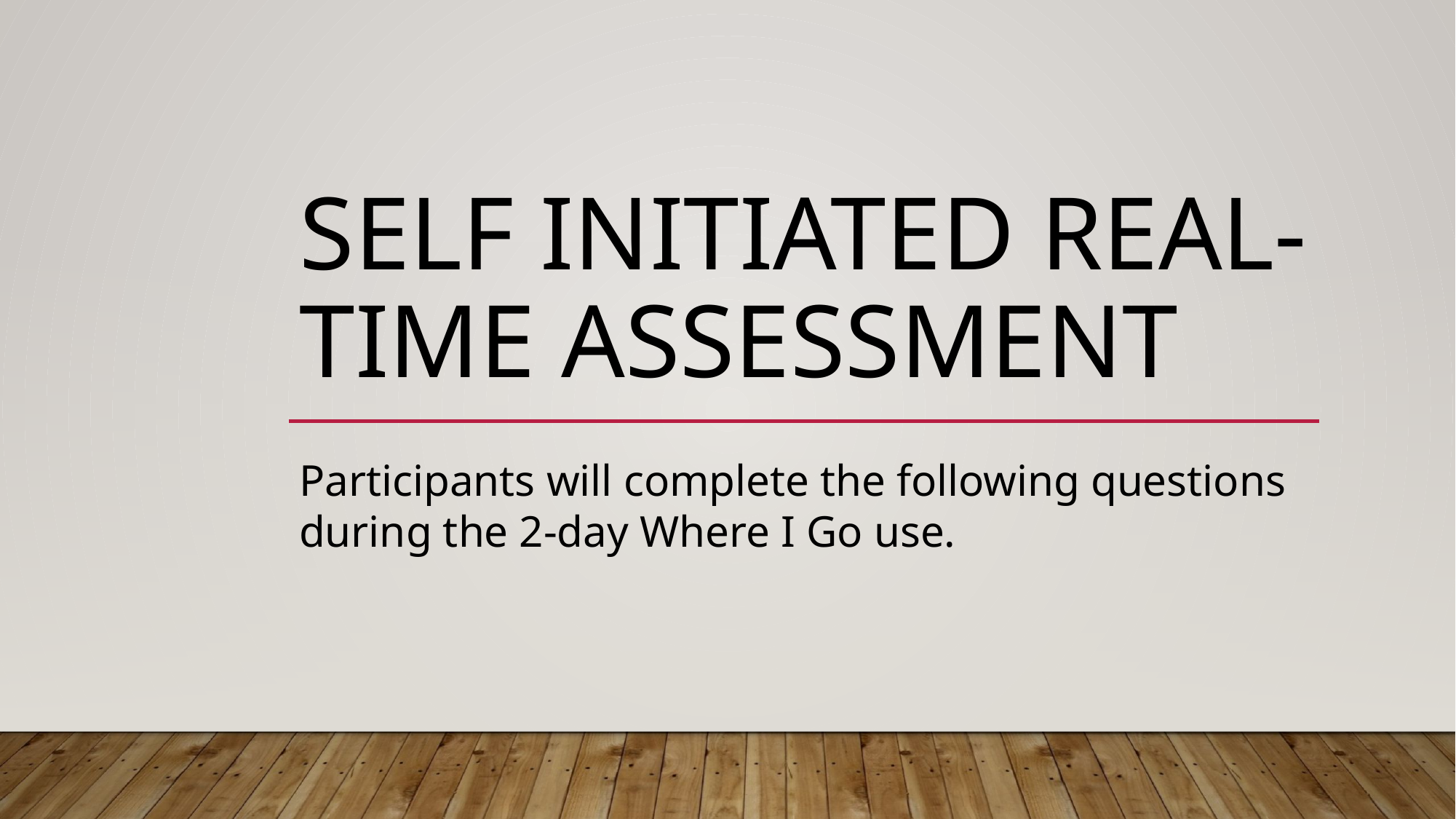

# Self Initiated Real-time Assessment
Participants will complete the following questions during the 2-day Where I Go use.

## Slide 17
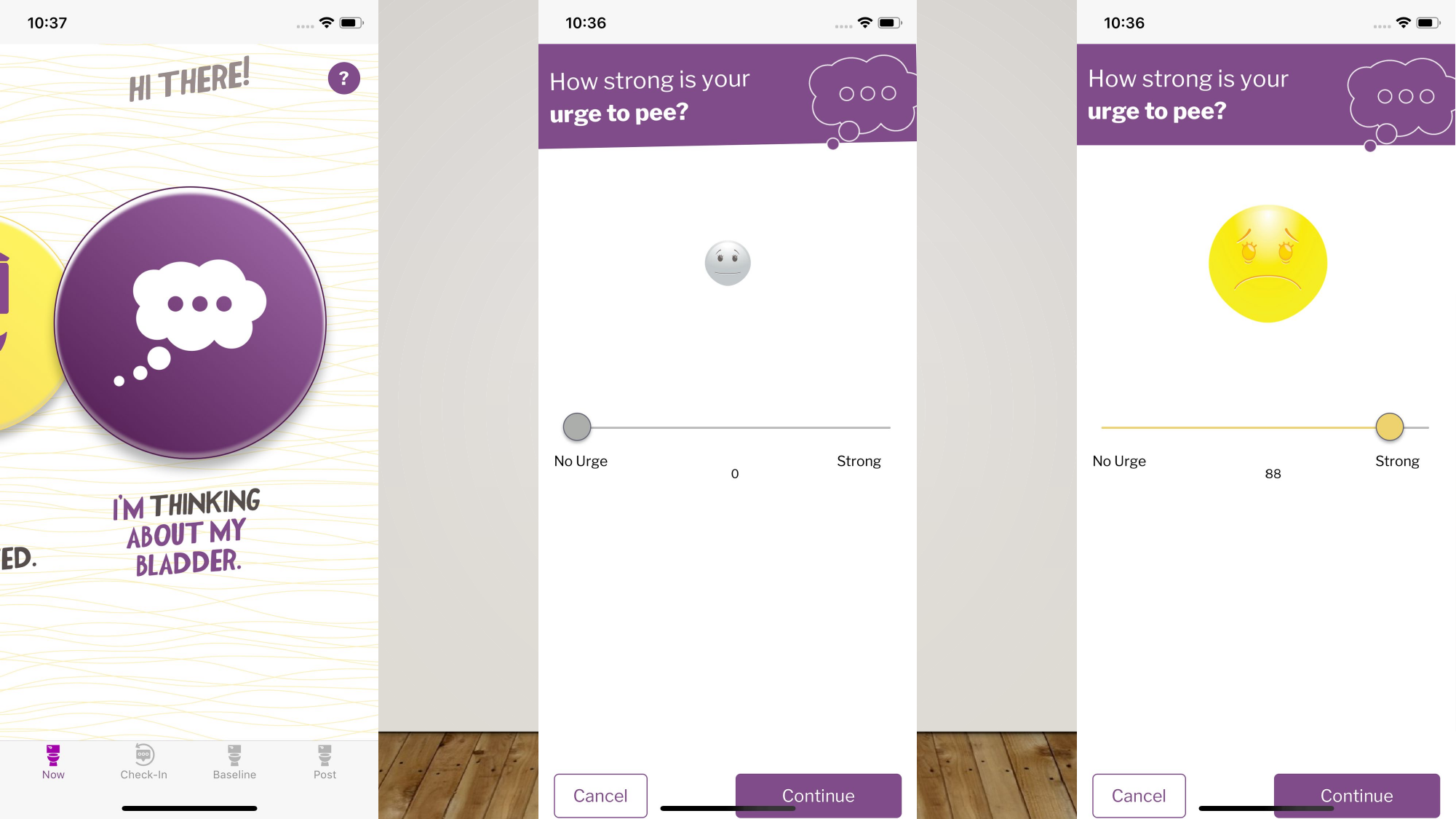

## Slide 18
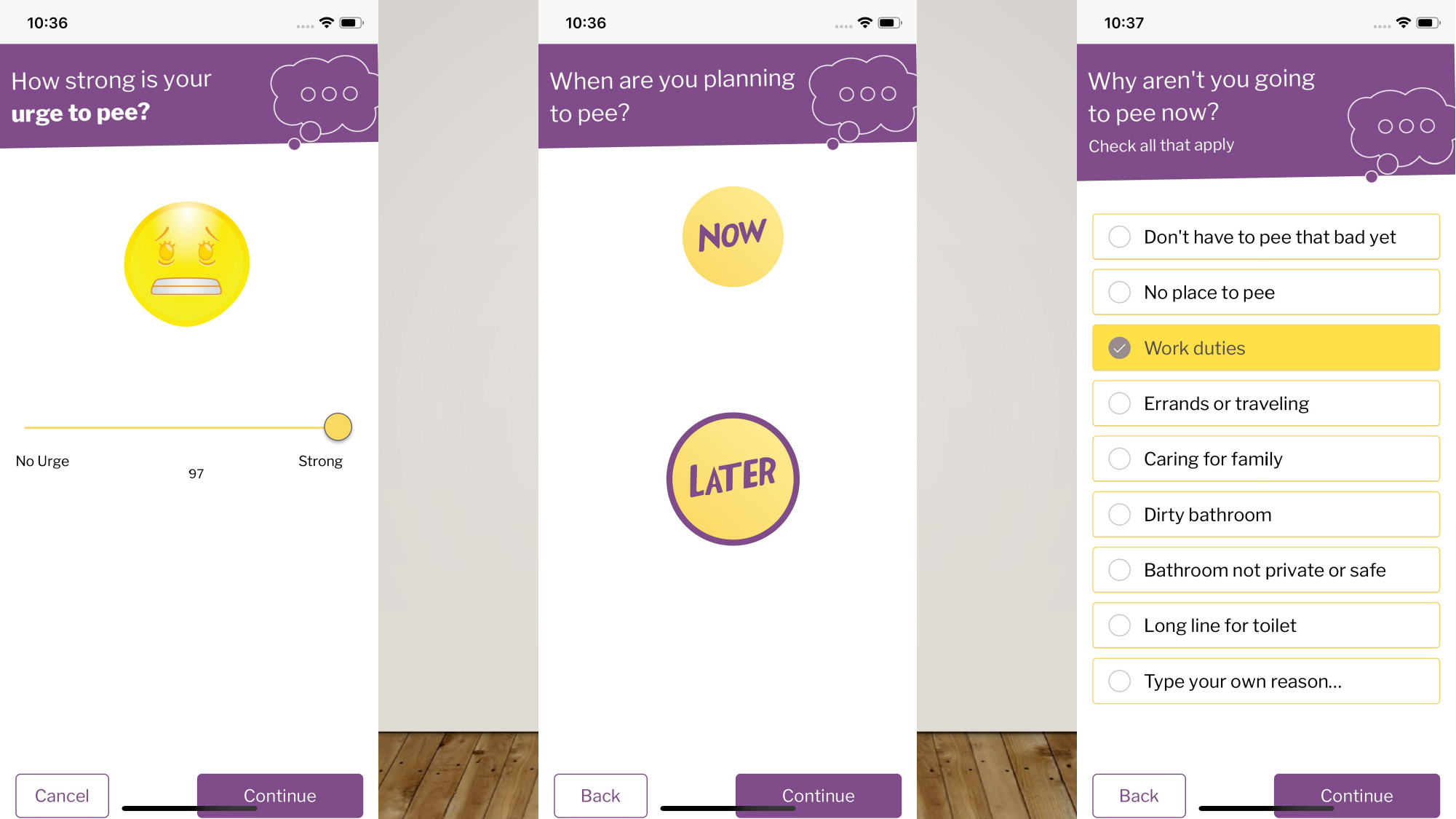

## Slide 19
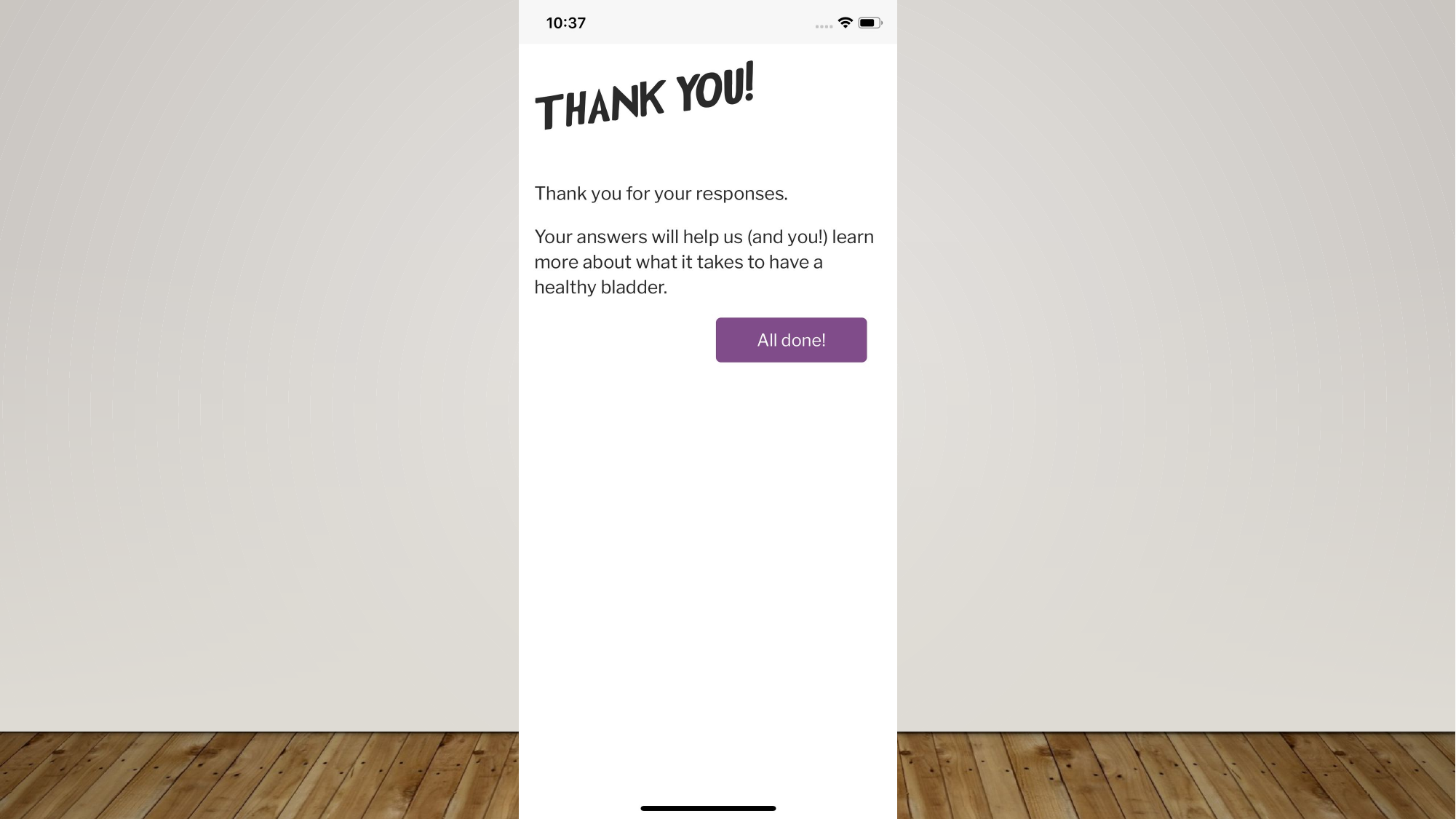

## Slide 20
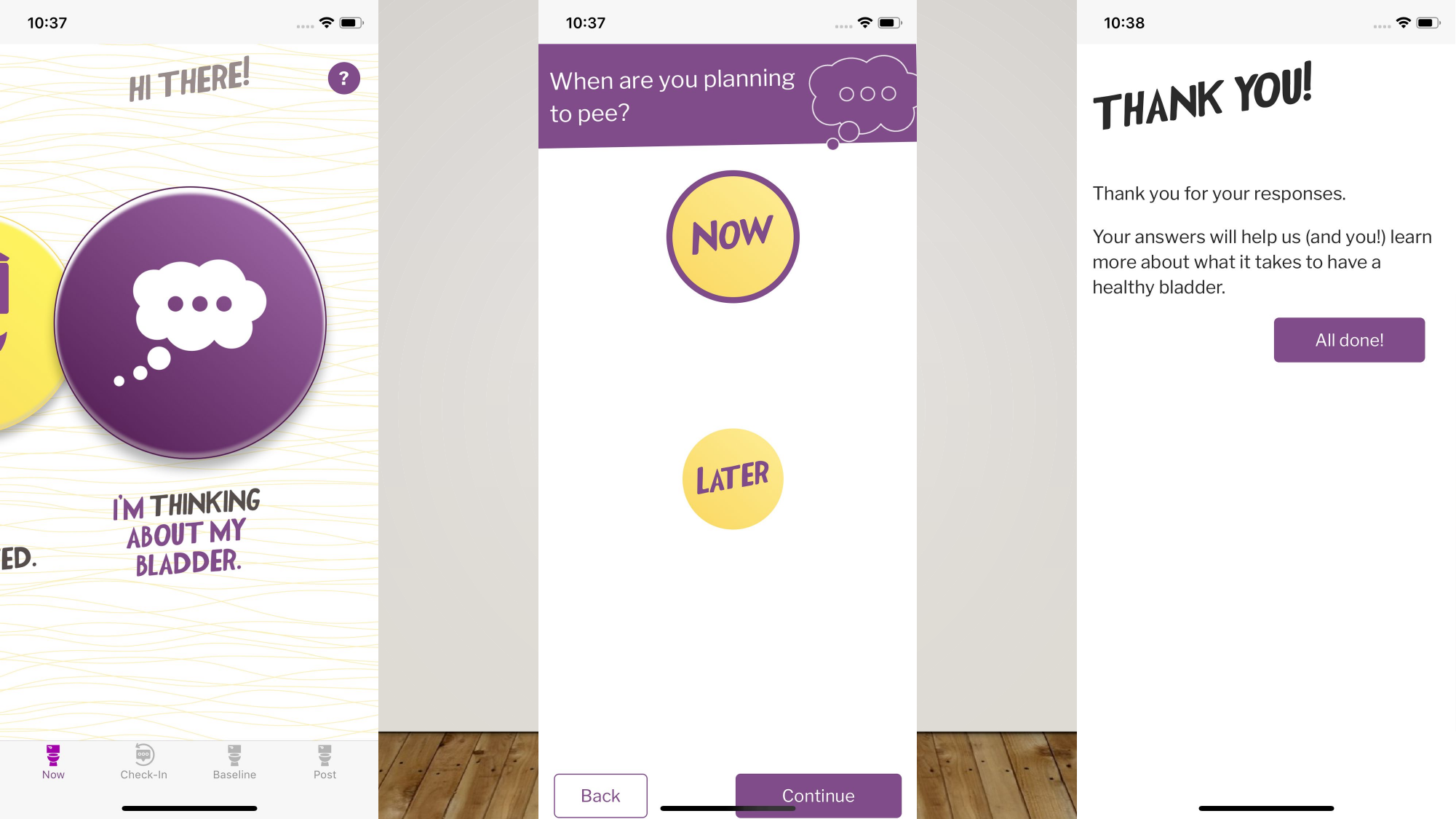

## Slide 21
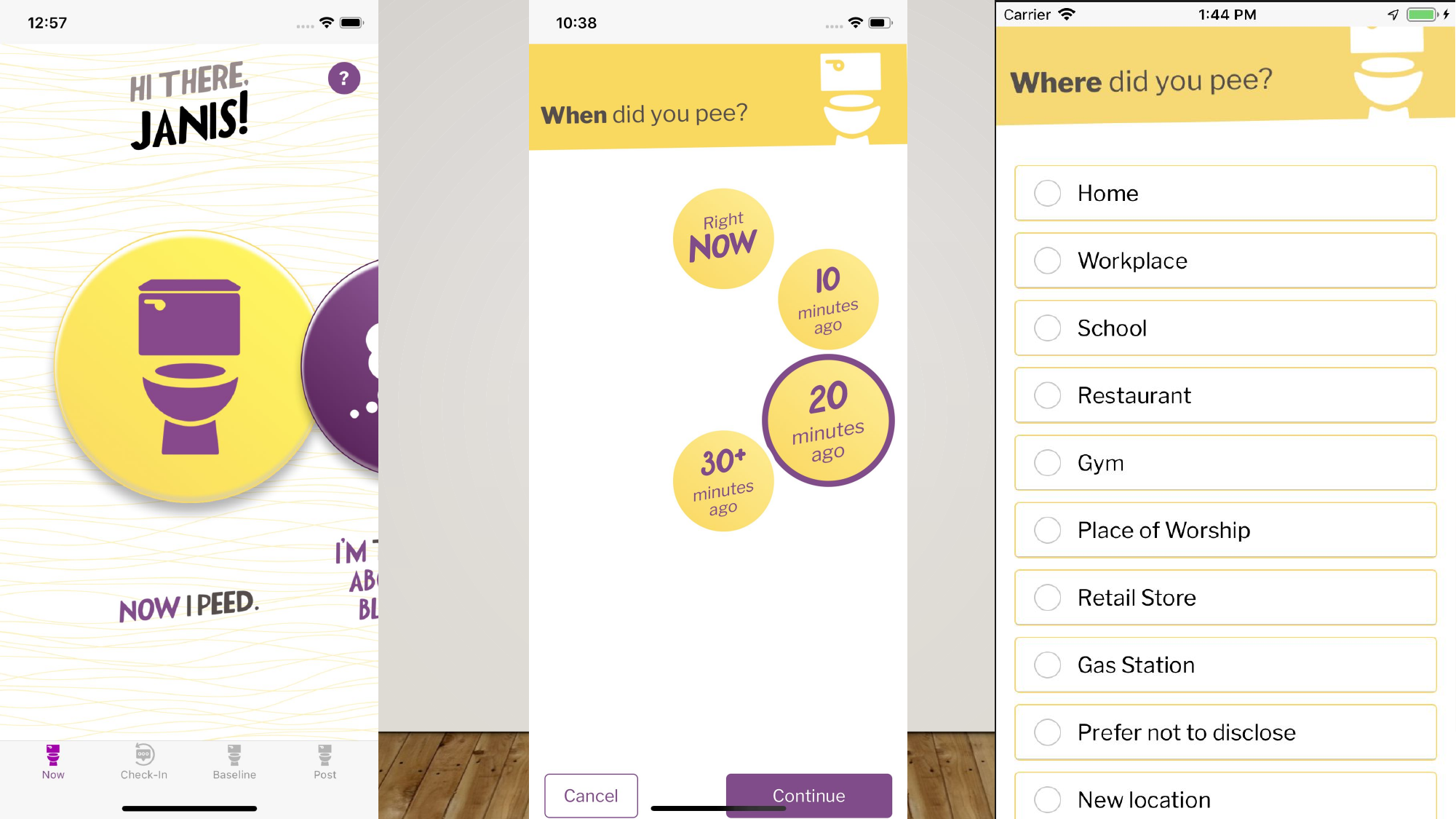

## Slide 22
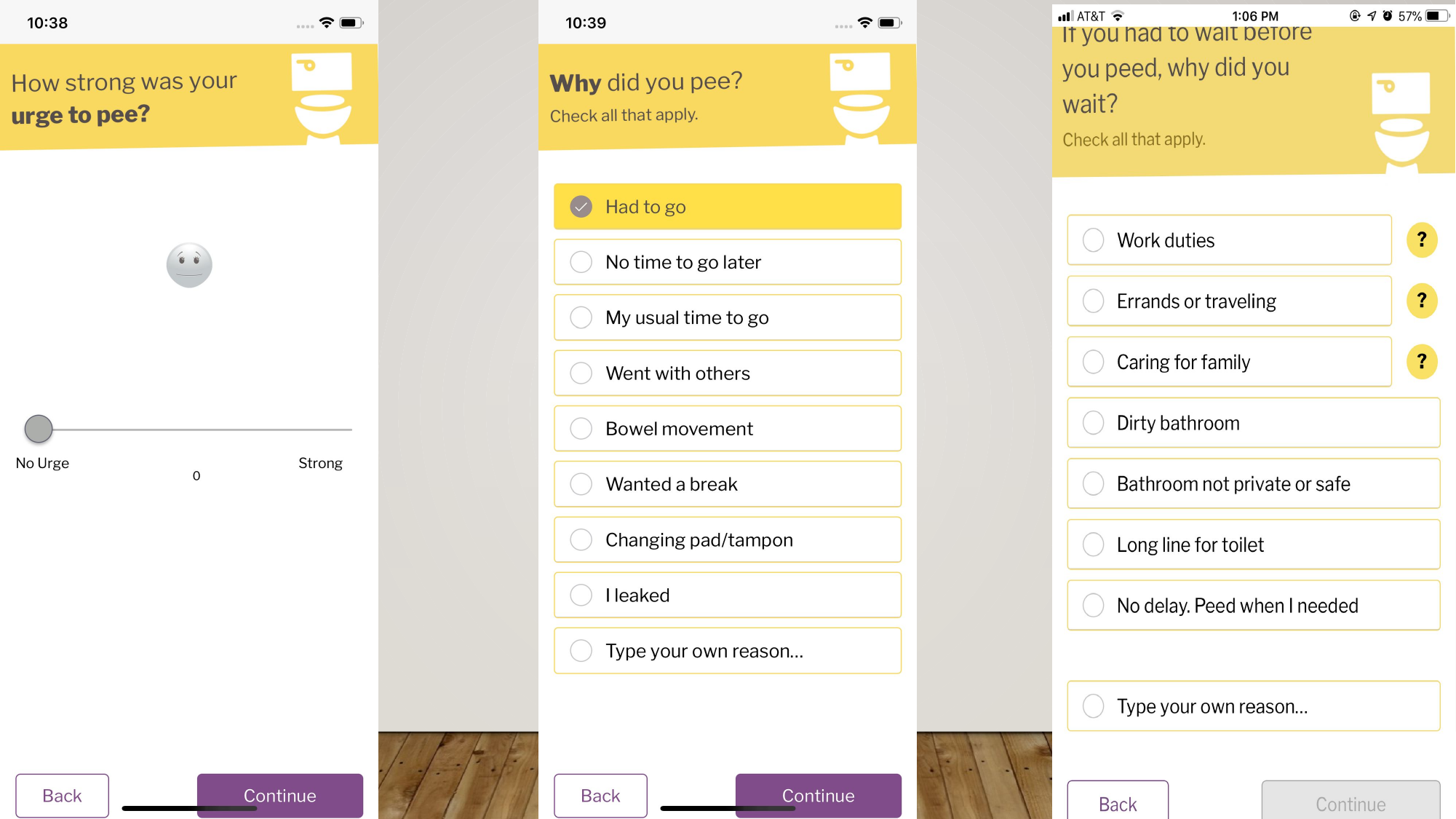

## Slide 23
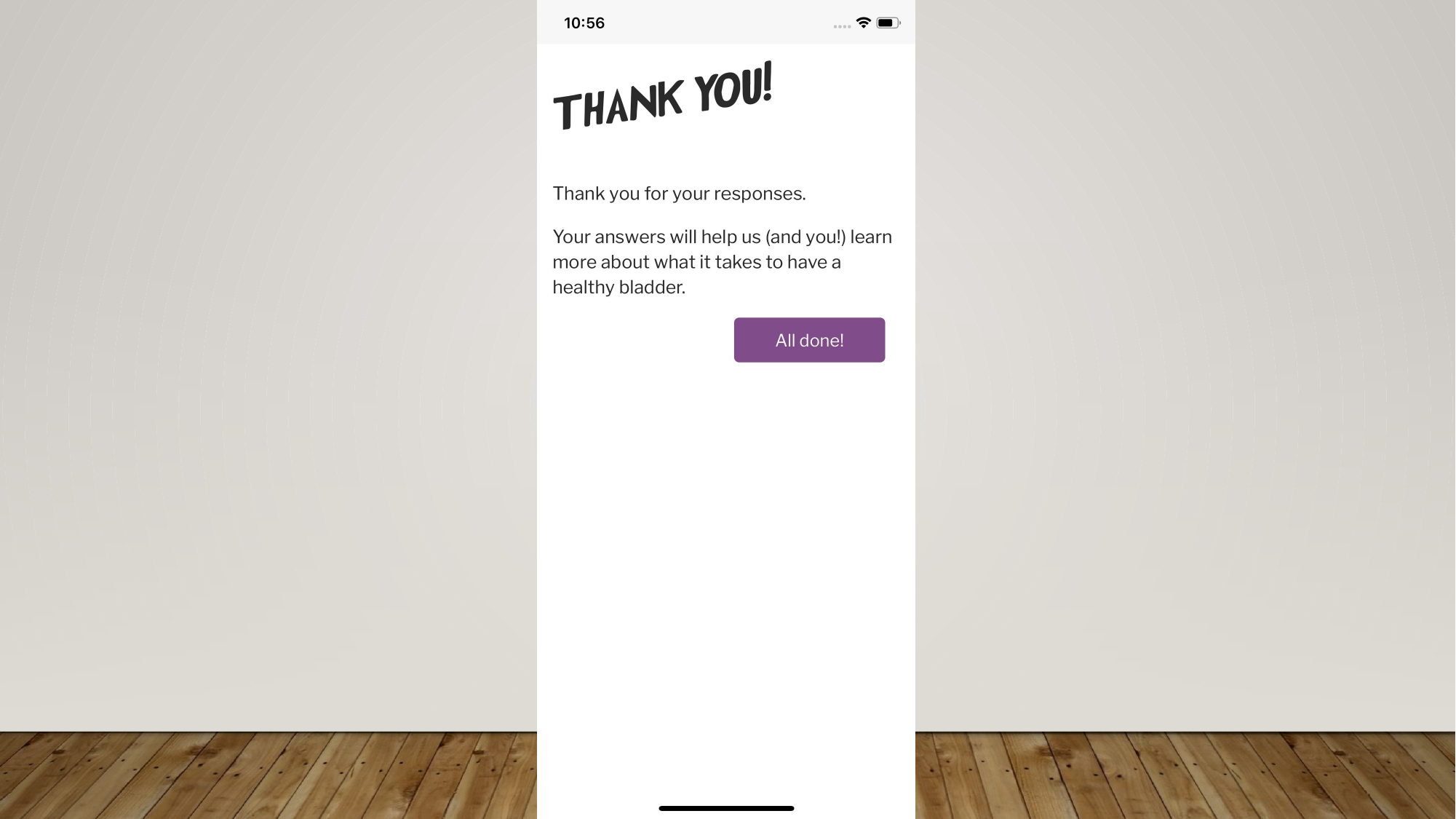

## Slide 24
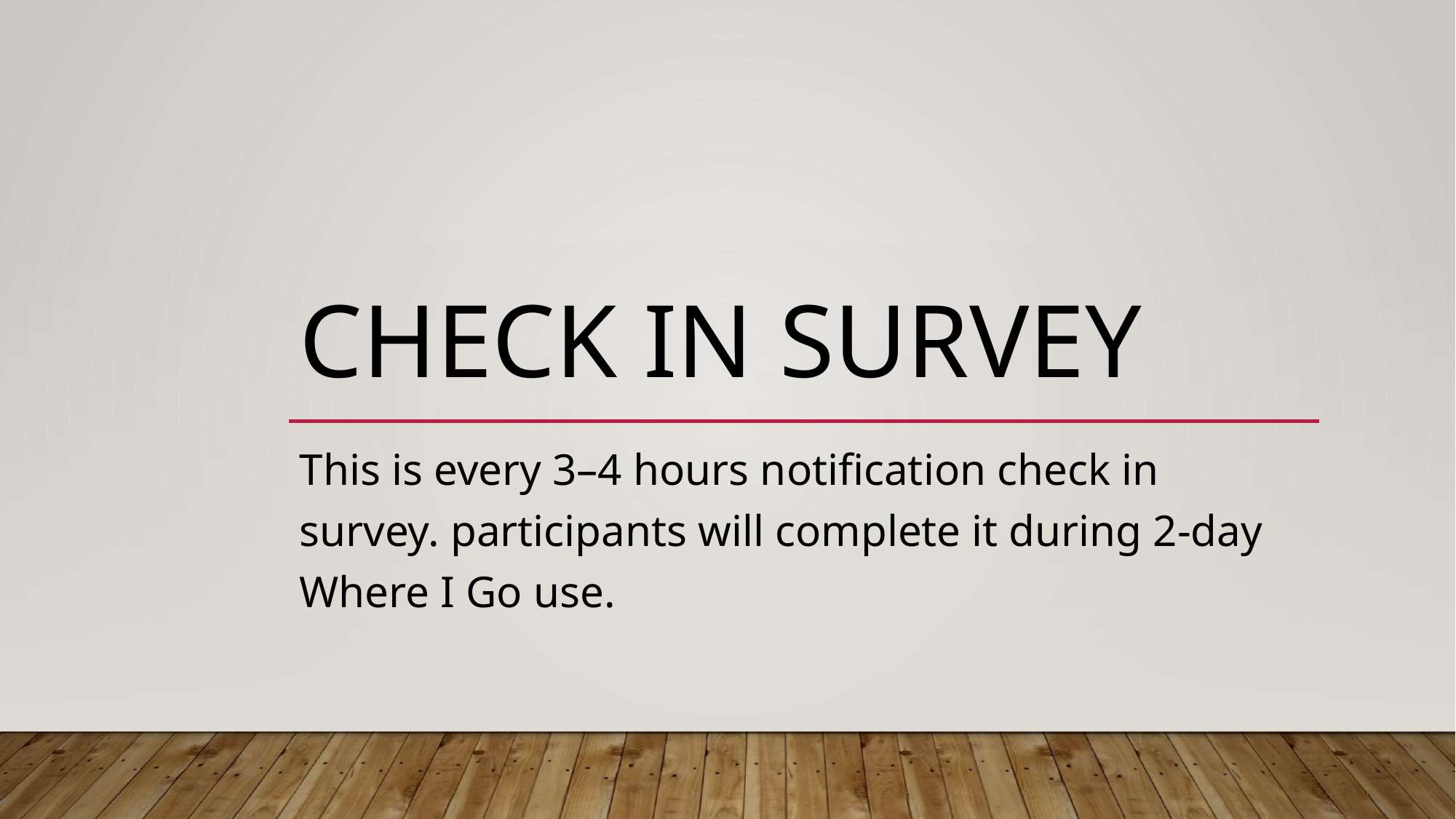

# Check in survey
This is every 3–4 hours notification check in survey. participants will complete it during 2-day Where I Go use.

## Slide 25
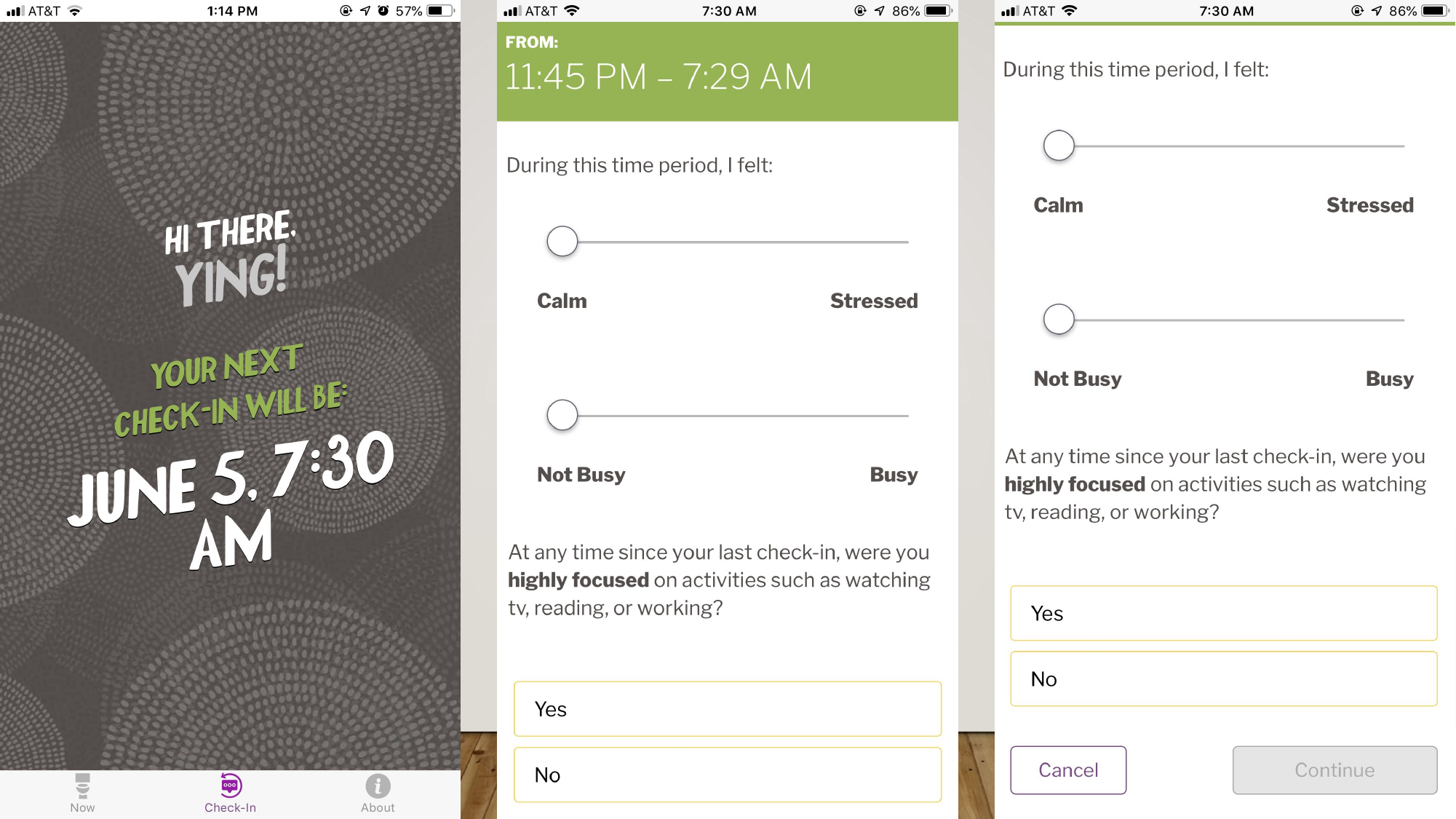

## Slide 26
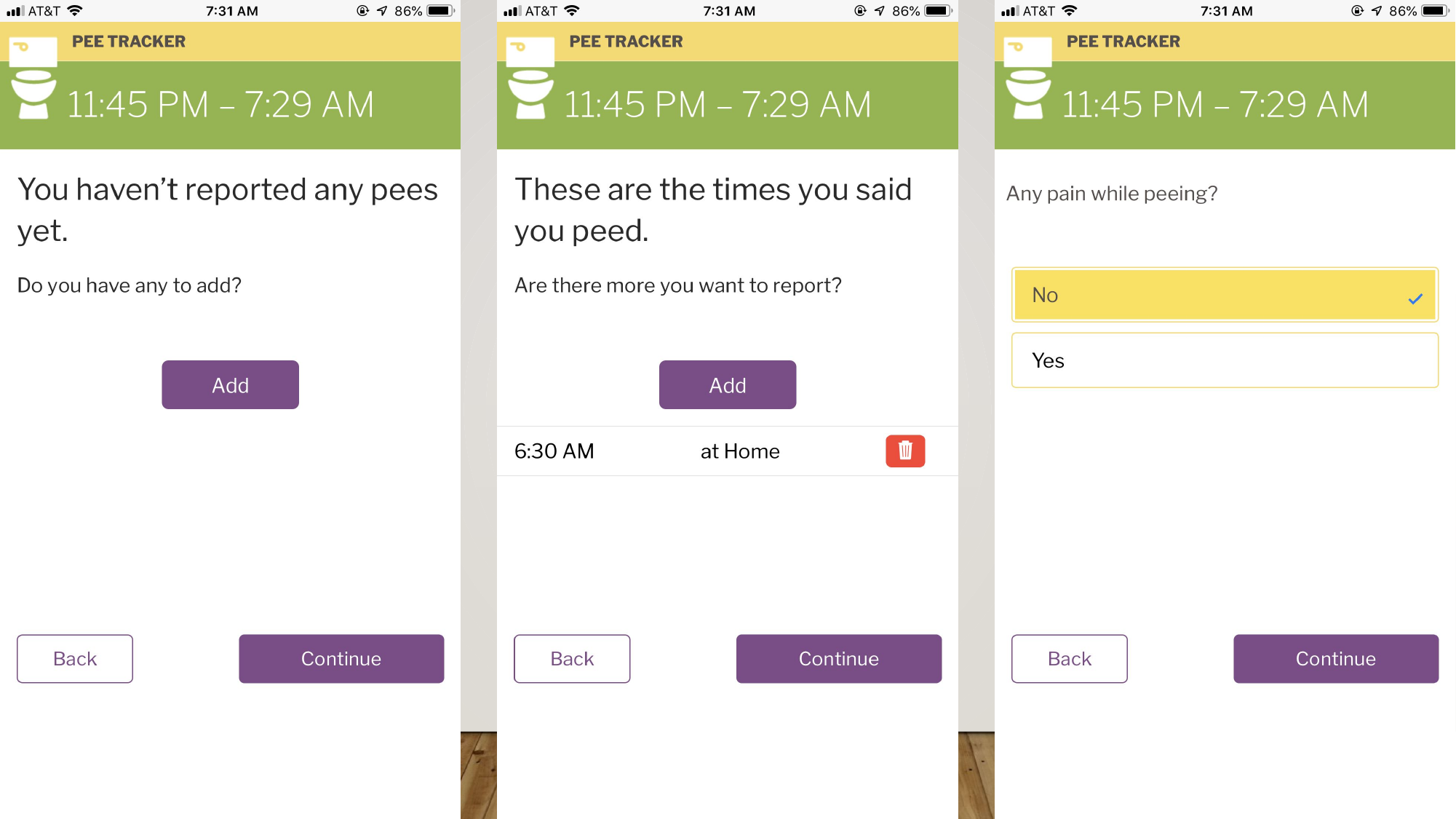

## Slide 27
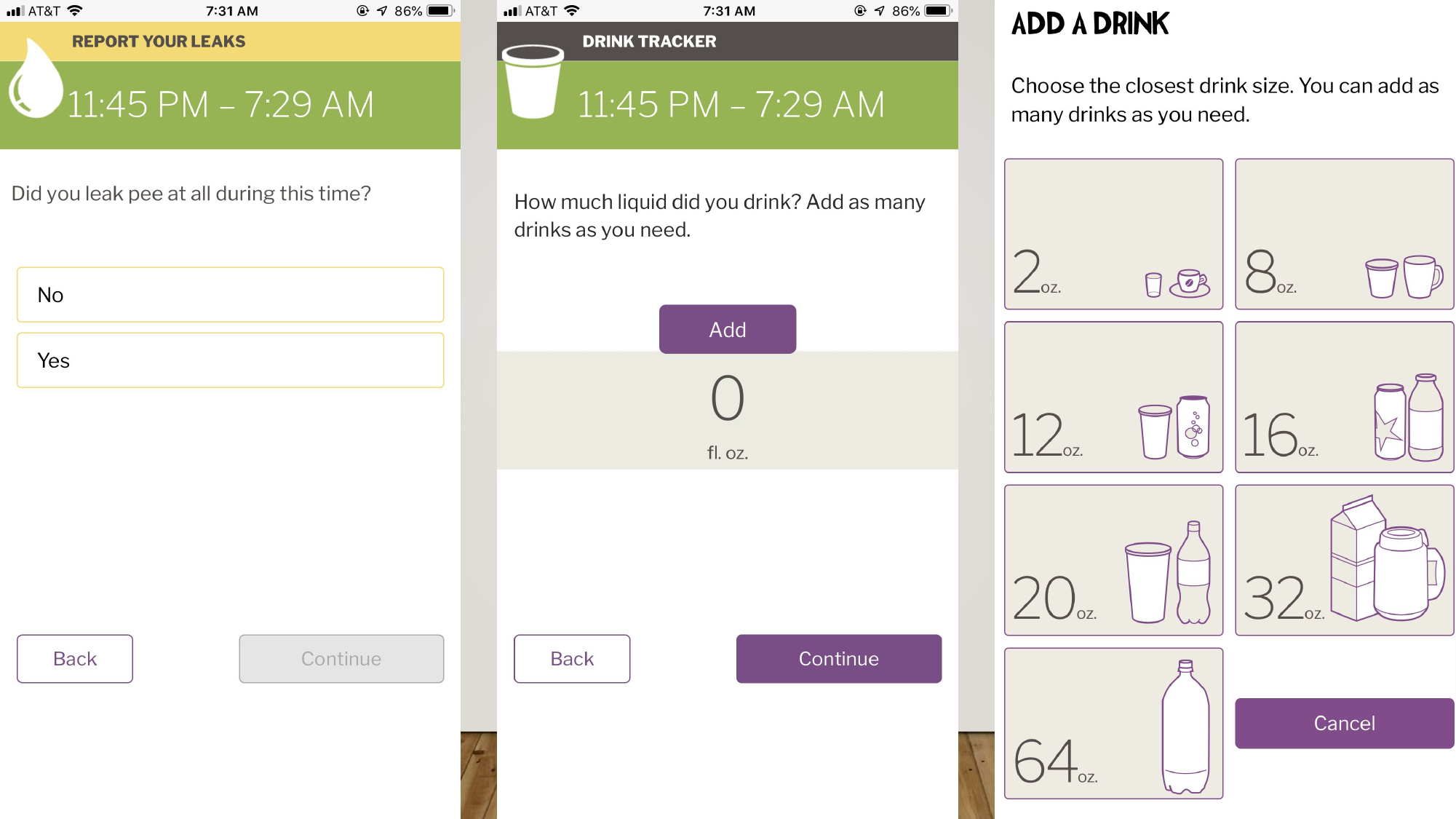

## Slide 28
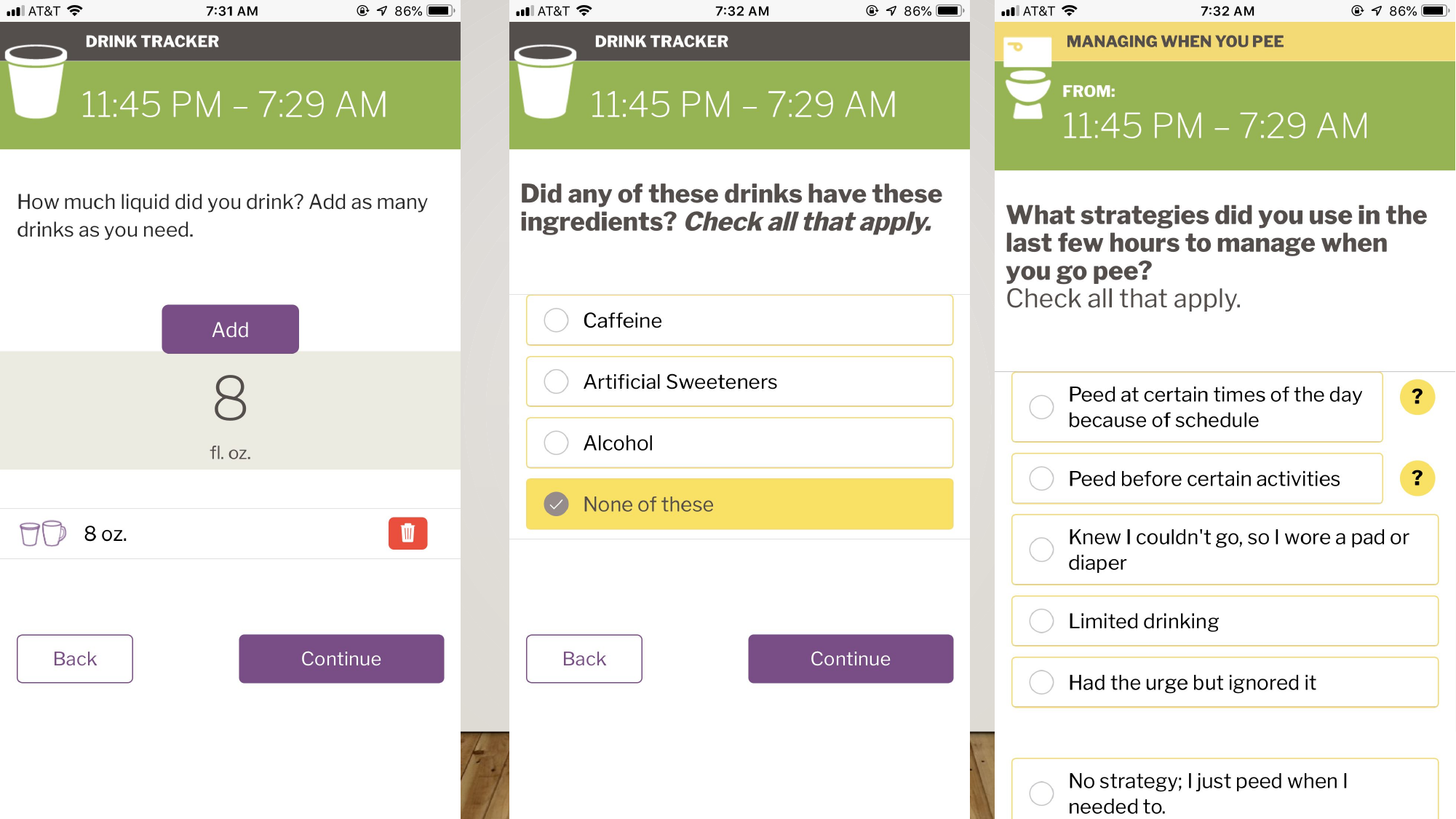

## Slide 29
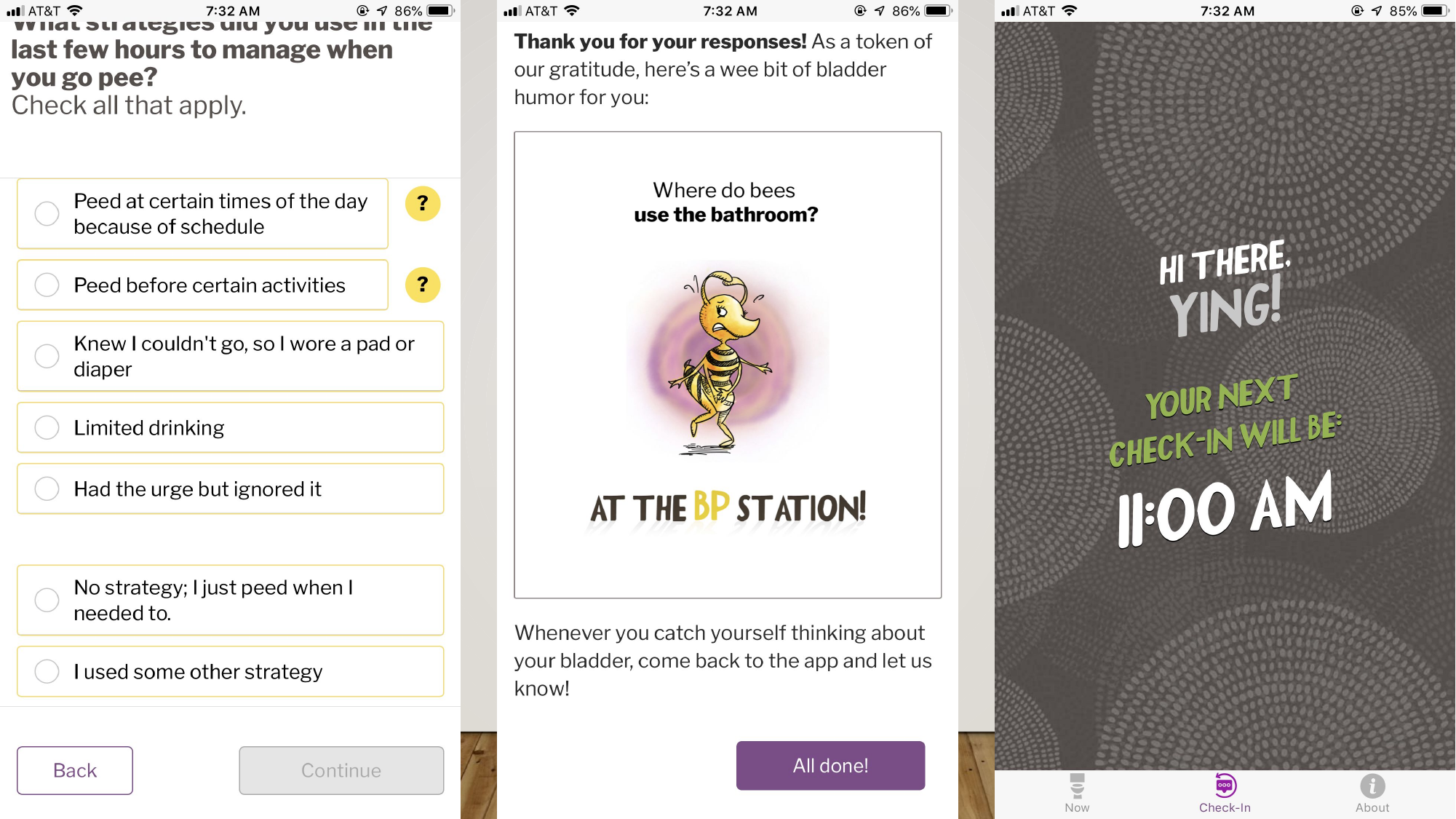

What strategies did you use
to manage when you go pee?
I just peed when I
needed to.

## Slide 30
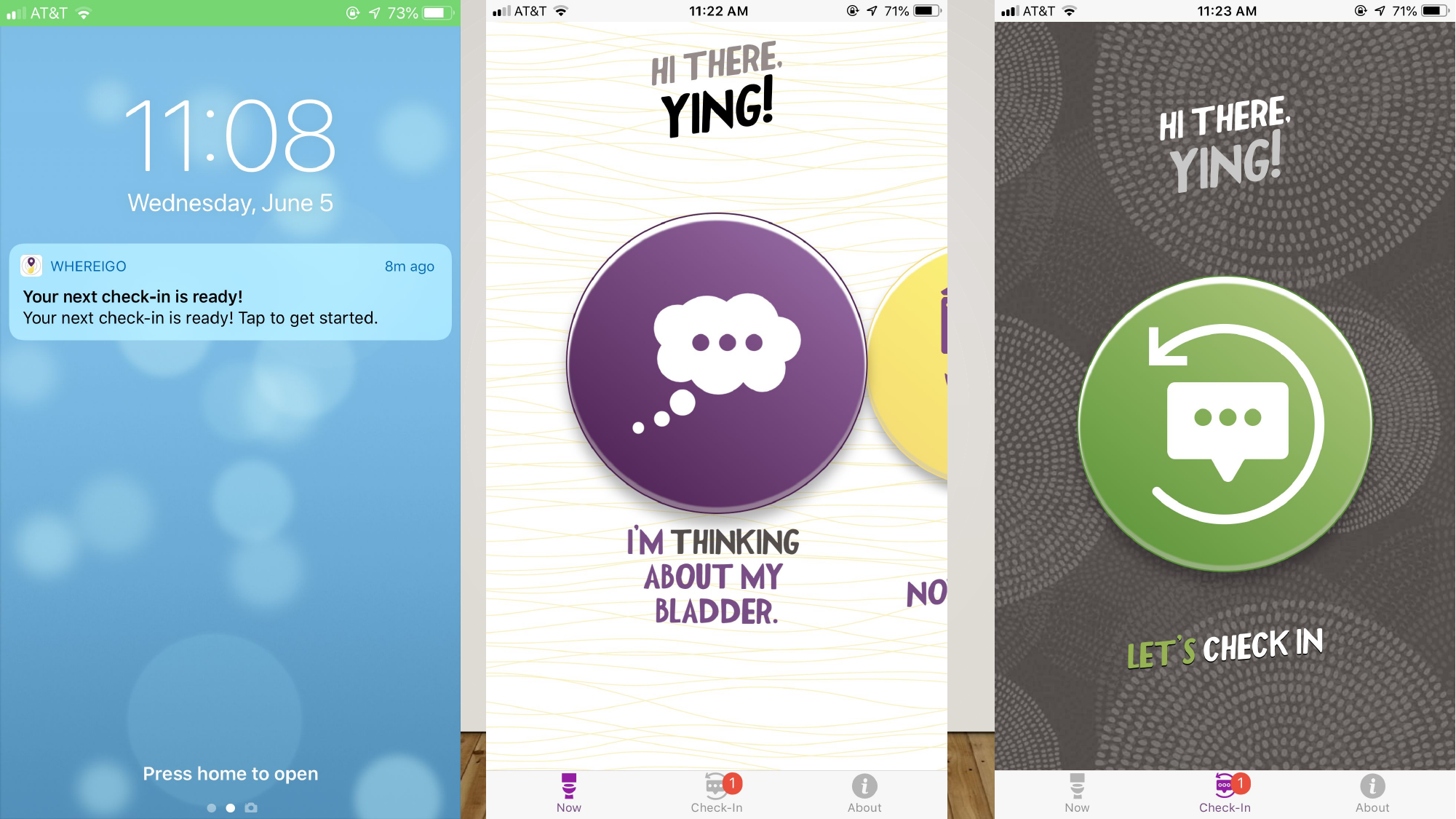

## Slide 31
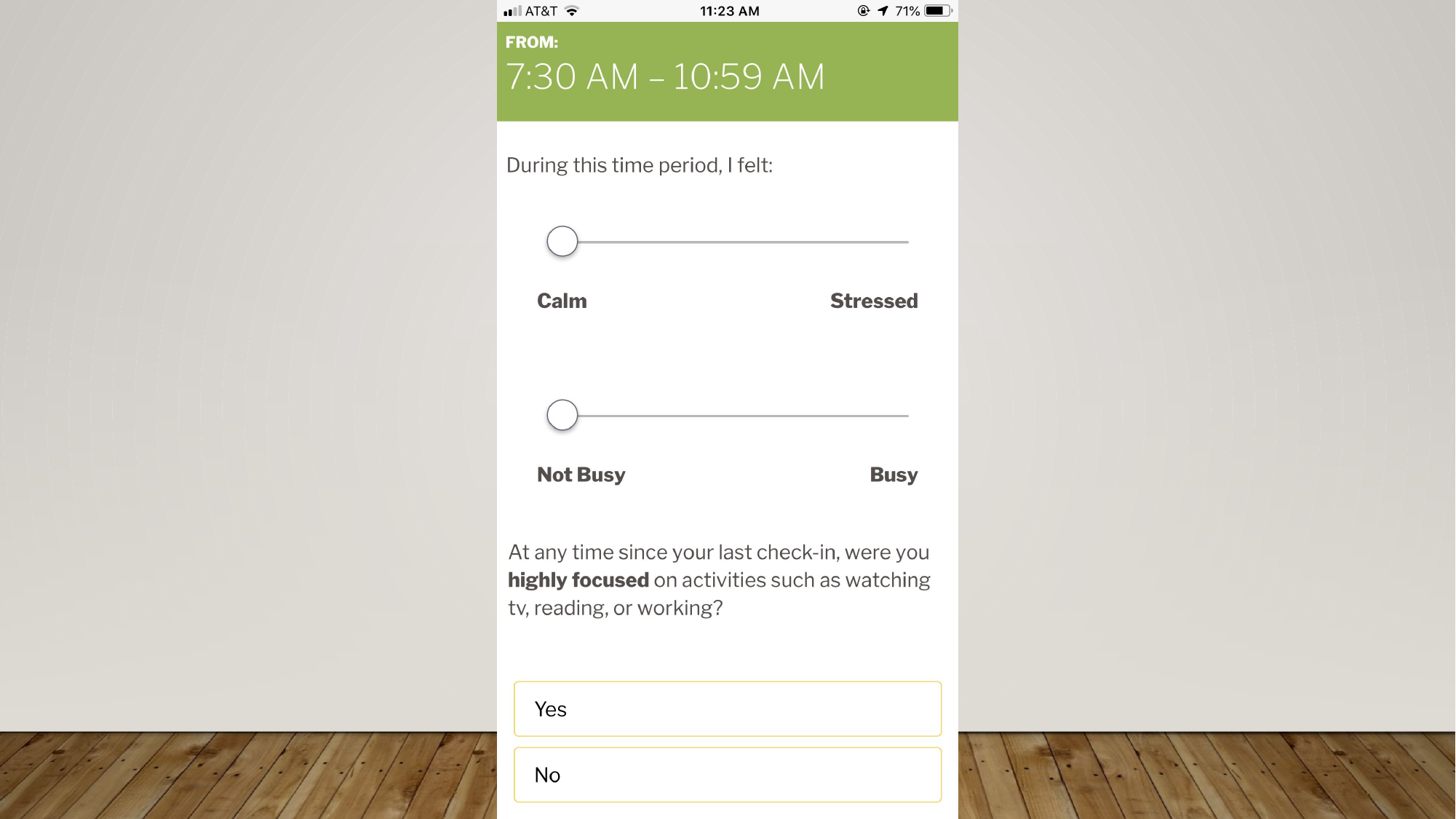

## Slide 32
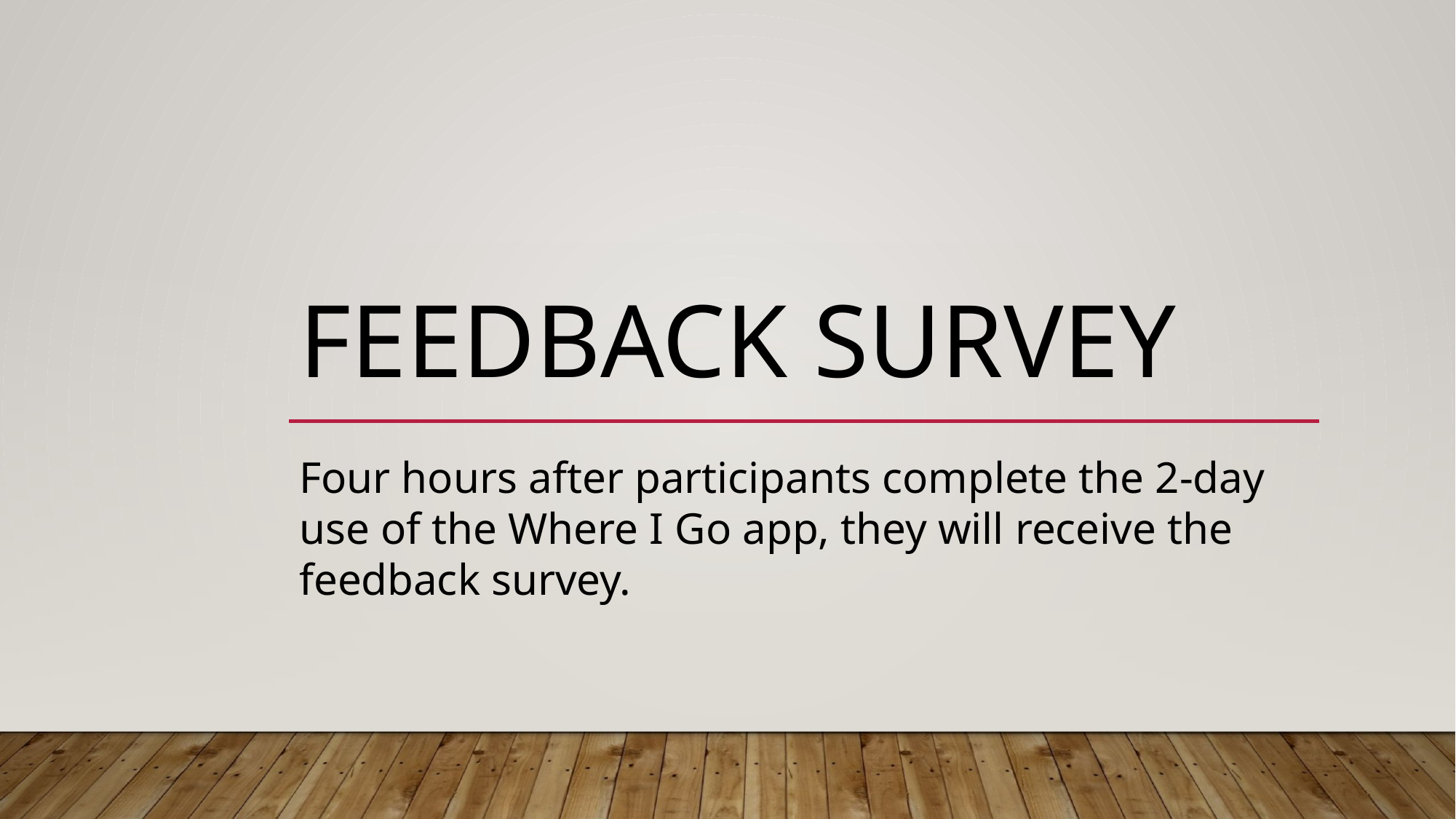

# FEEDBACK Survey
Four hours after participants complete the 2-day use of the Where I Go app, they will receive the feedback survey.

## Slide 33
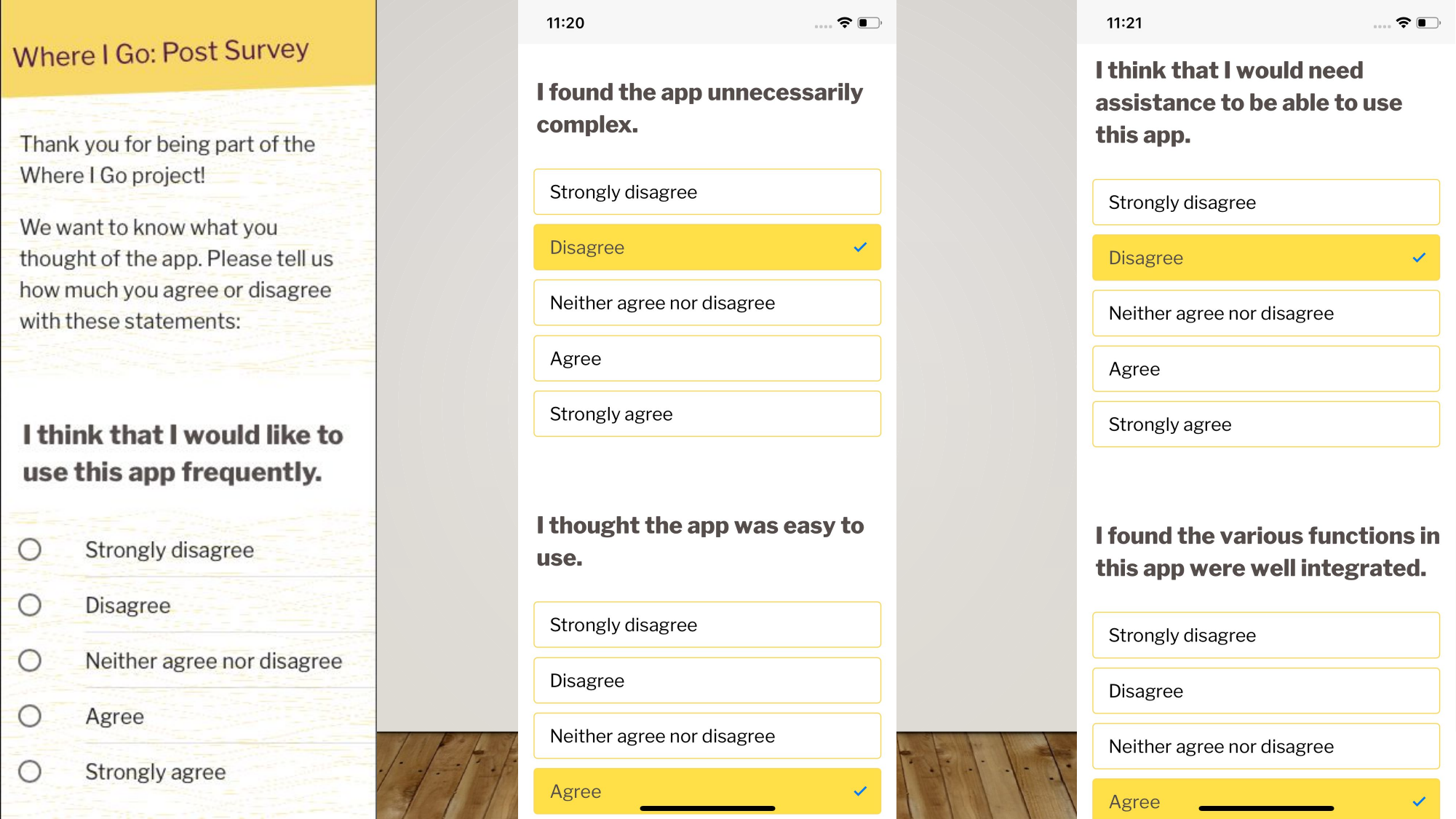

## Slide 34
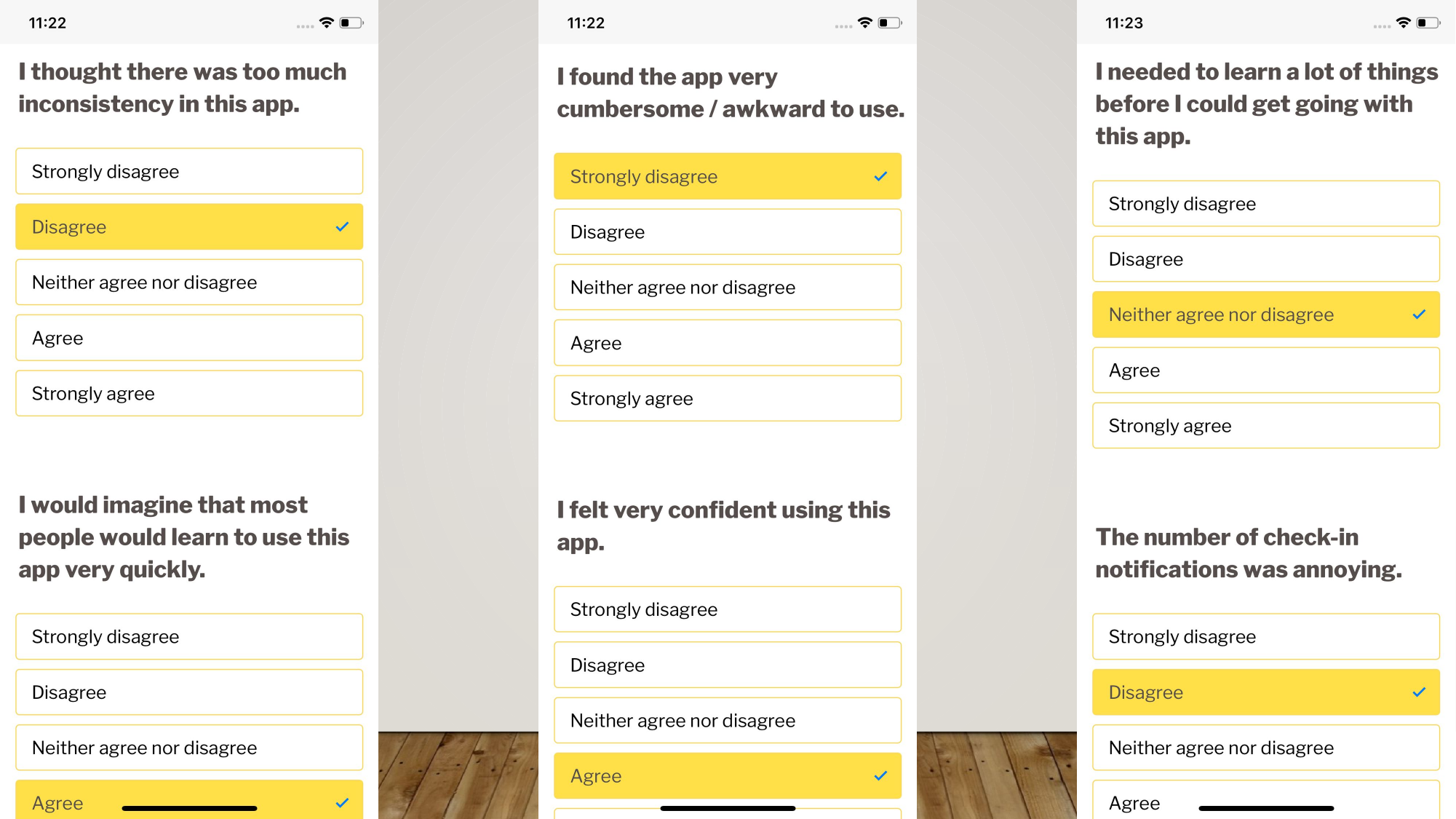

## Slide 35
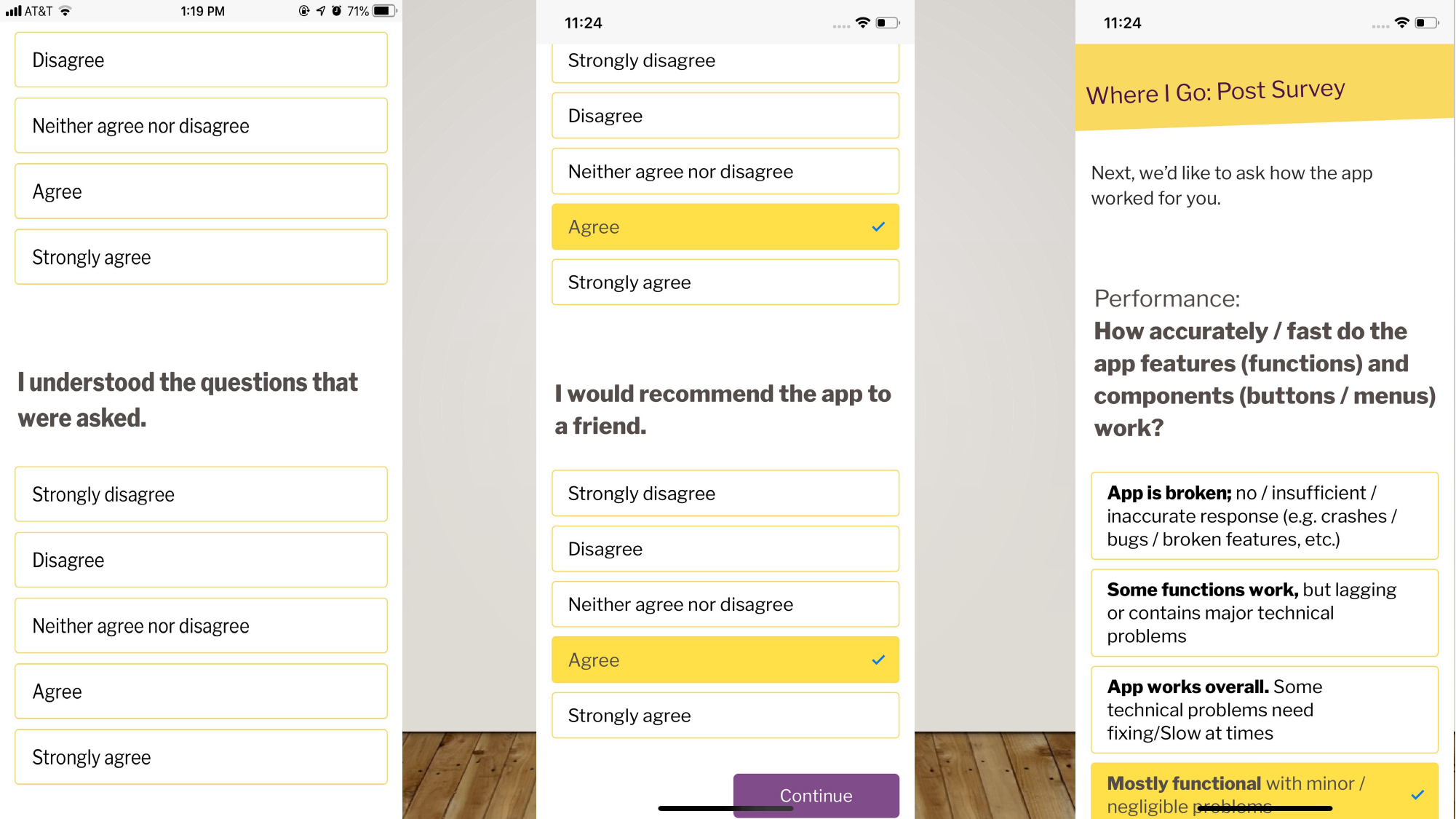

## Slide 36
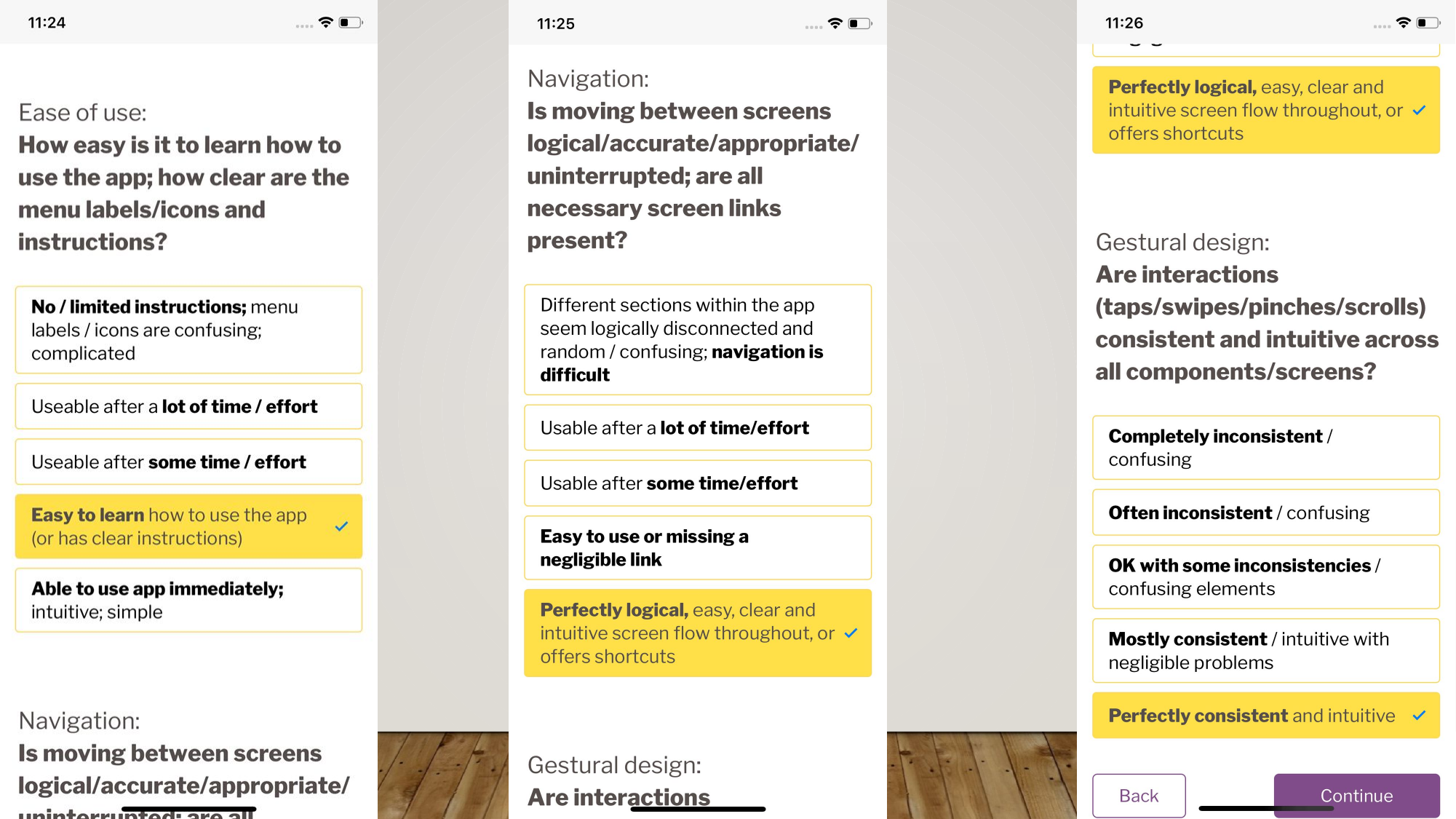

## Slide 37
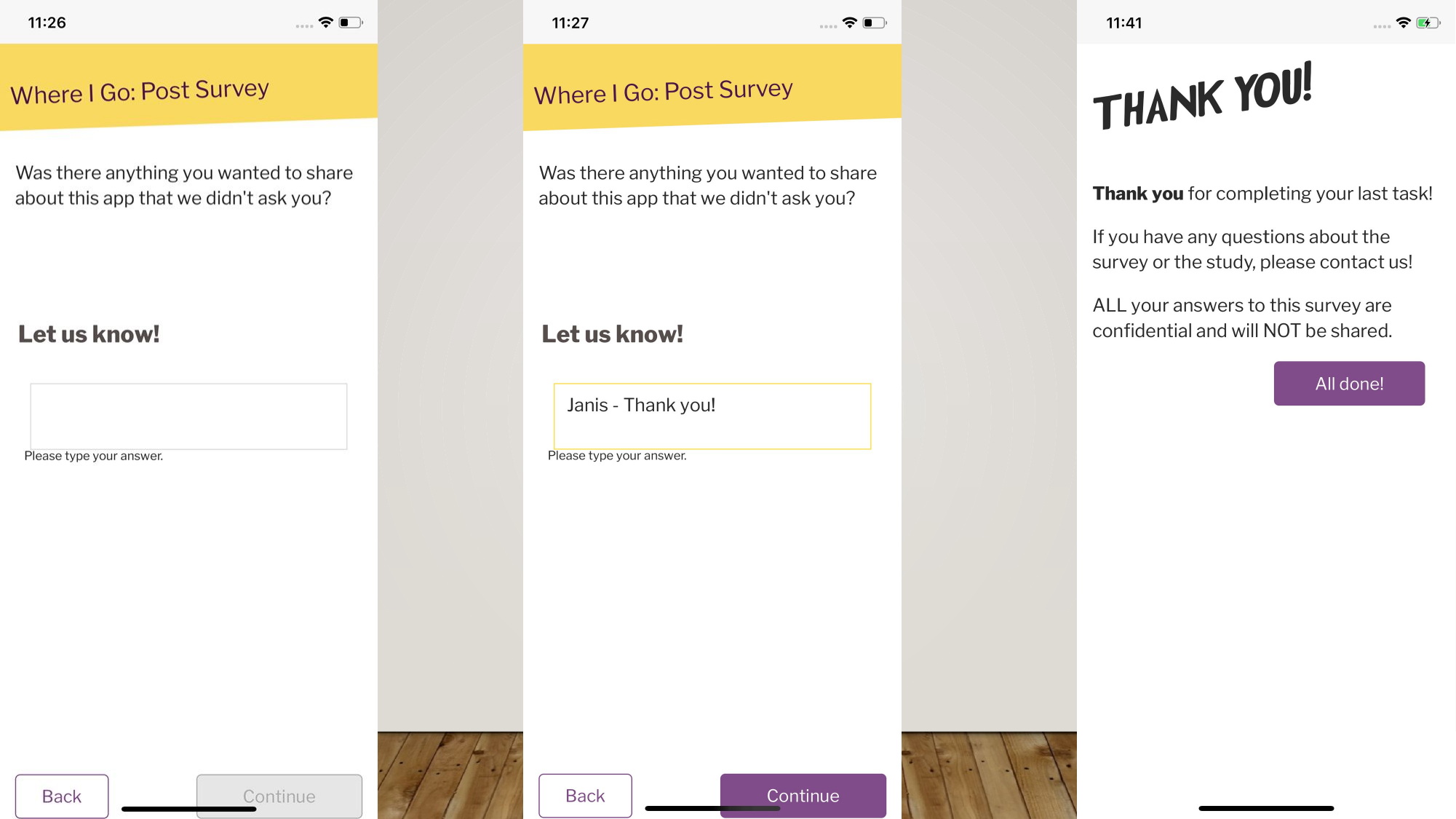

Supplement: Multimedia Appendix 1 [file resprot_v13i1e54046_app1.pptx]
