# Supplementary material for: Design of a Tool Capable of Assessing Environmental Sociocultural Physical Factors Influencing Women’s Decisions on When and Where to Toilet Within Real-World Settings: Protocol for the Build and Usability Testing of a Mobile App for Use by Community-Dwelling Women
Source: JMIR Res Protoc. 2024 Sep 18;13:e54046. doi: 10.2196/54046 (PMC11447419; doi:10.2196/54046)
Supplement: Multimedia Appendix 7 [file resprot_v13i1e54046_app7.docx]

**
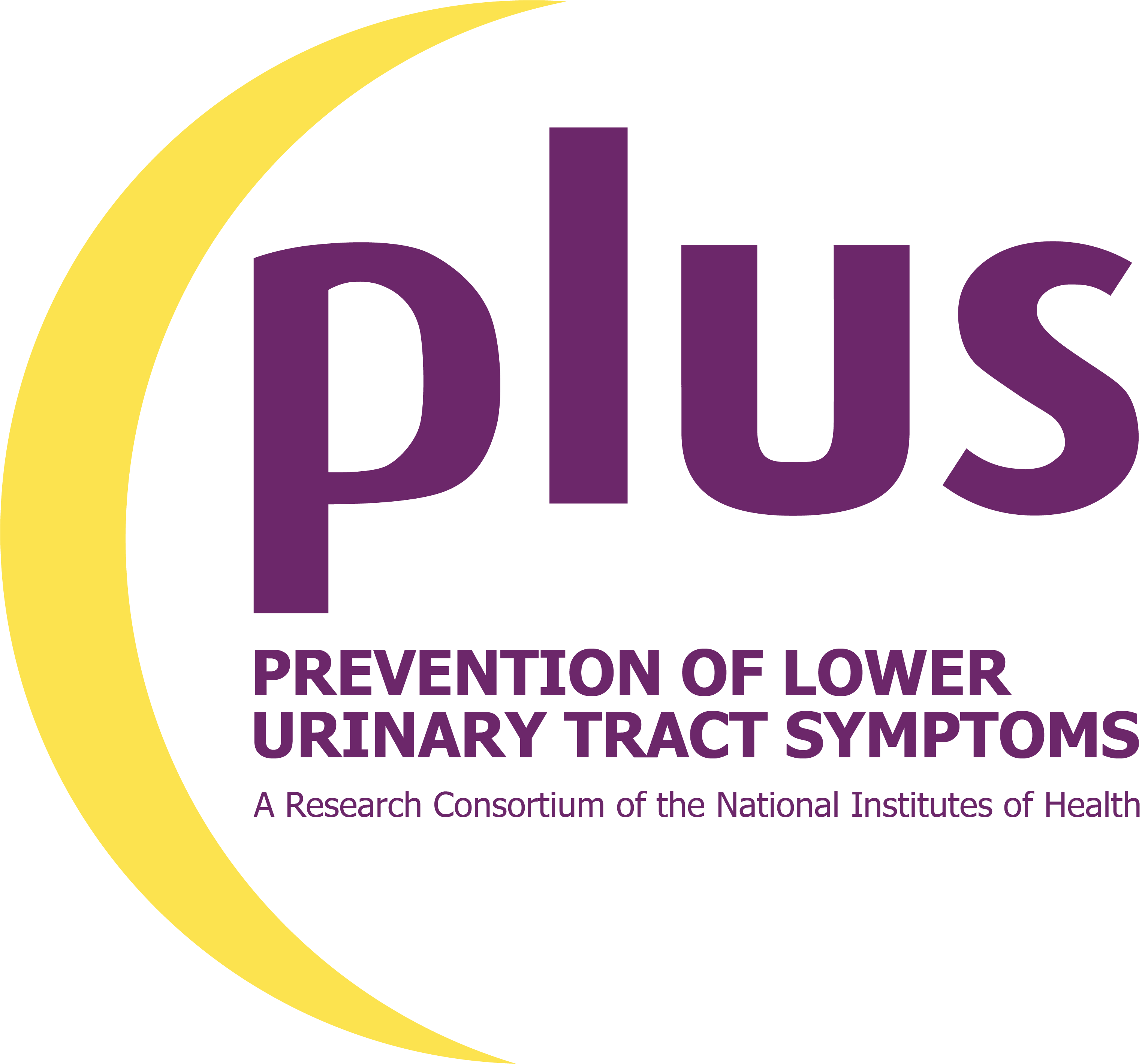
**

**Title: PLUS Where I Go Mobile Application**

A Protocol for the Prevention of Lower Urinary Tract Symptoms (PLUS) Consortium

**July 30, 2019**

**University of Michigan Version 1.5**

| **Study Title** | PLUS Where I Go Mobile Application |
| --- | --- |
| **Study Design** | Prospective observational |
| **Primary Objective** | To establish feasibility, acceptability of use, and the extent to which all questions are answered (data completeness) |
| **Secondary Objective(s)** |  |
| **Research Intervention(s)/Investigational Agents** | N/A |
| **IND/IDE # (if applicable)** | N/A |
| **Investigational Drug Services # (if applicable)** | N/A |
| **Study Population** | Adult English-speaking community women |
| **Sample Size** | N = 60 across sites  University of Michigan = 24; other sites = 12 each |
| **Study Duration for Individual Participants** | Two in person visits will last about 40-60 minutes. Actual use of Where I Go is estimated to take approximately one hour total across the consecutive 48 hours of its use. |

[I. Rationale and Background 4](#_Toc13582646)

[II. Objectives 5](#_Toc13582647)

[III. Specific Aims 5](#_Toc13582648)

[IV. METHODOLOGY 6](#_Toc13582649)

[A. Overview 6](#_Toc13582650)

[B. Instruments embedded in the “Where I Go” Application 6](#_Toc13582651)

[C. Recruitment 8](#_Toc13582652)

[D. Visit one 9](#_Toc13582653)

[E. Between visits (48 hours) 10](#_Toc13582654)

[F. Visit two 10](#_Toc13582655)

[Data Retention and/or Data Destruction Plan 10](#_Toc13582656)

[G. Statistical Considerations 13](#_Toc13582657)

[V. Expected Outcomes of the Study 14](#_Toc13582658)

[VI. Duration of the Project 14](#_Toc13582659)

[VII. Compensation 14](#_Toc13582660)

[VIII. Project Management 14](#_Toc13582661)

[IX. Risks and Benefits 15](#_Toc13582662)

[A. Risks 15](#_Toc13582663)

[B. Benefits 15](#_Toc13582664)

# **Rationale and Background**

Mobile/smart phones are used across diverse segments of the population and are a compelling vehicle for research data collection. According to the PEW Research Center, 77% of adults in the United States own a smartphone (2016) with the highest adoption rates seen primarily in adults with college degrees who are young and upper income earners. Smartphones offer unique opportunities to engage individuals and understand how the physical and social environment influences their health. Users typically carry their phones with them, interact with their phones multiple times each day (e.g. nearly 100 phone “touches” per day on average), and spend several hours per day engaged in activities that were once only possible on desktop or laptop computers (e.g. viewing, searching, messaging, gaming). Many phones also contain unobtrusive motion sensors, GPS-enabled time and location tagging, and up-to-the-minute phone and app usage statistics that can be used to characterize some aspects of an individual’s interactions with the physical and social environment.

As a result, the NIDDK-funded PLUS Consortium has chosen to investigate the utility of a Mobile Bladder Application to study bladder health in community women and girls. The **long-term goal** of a Mobile Bladder Application is to enhance our understanding of the wide variance in bladder habits in varied environments over time and to identify the underlying individual risk and protective factors, so we inform broad scale public health education on best practices for lifelong bladder health. Collecting data via the app will contribute information that supports the overall PLUS Consortium goals. These are to study the determinants of bladder health, identify risk factors for lower urinary tract symptoms and conduct prevention and interventional studies in women. The **short-term goals** are to 1) build a tool to measure bladder habits and special/temporal associations in women and 2) evaluate the ease of usability for participants.

The PLUS mobile bladder application called “Where I Go” is designed to be a technologically intensive data collection vehicle. The long-term goal is to use Where I Go in studies that aim to obtain a clearer picture of how bladder habits are related to factors in a woman’s environment (home, work, and public spaces). Baseline demographics will be collected prior to using the app. Once the app is downloaded, the woman will report bladder habits, environment and social factors over a 48 consecutive hour time period. This will allow us to study the associations between individual behaviors, environmental factors and ways in which women manage their bladders in day-to-day life. Additional to the woman’s environment (home, work and public spaces), Where I Go will offer the woman opportunities throughout her day to record her fluid intake, and toileting factors such as whether she is able to go to the bathroom on her own accord or is restricted in some way, what adaptive behaviors she has to make, and whether or not she has ready access to toileting.

# **Objectives**

The overarching goal of Where I Go Stage 3 development is to establish feasibility, acceptability of use, and the extent to which all questions are answered (data completeness). Our goal is to achieve user testing with approximately 60 English-speaking community women across sites, with 24 user testers at the University of Michigan. We anticipate that additional testing will be needed for a more broad representation.

# **Specific Aims**

**AIM 1: To measure the user’s experience (acceptability) of the app, and refine as indicated by results.**

Hypothesis 1.1: The System Usability Scale (SUS) for participants will show a mean raw score of 74 or greater.

Hypothesis 1.2: The Functionality subscale of the Mobile Application Rating Scale (MARS) for participants will show a mean score of be 3.5 (based on 1-5 scale).

**AIM 2: Analyze user response rates of a.) The real-time self-initiated Where I Go prototype 1 components and b.) Where I Go mobile application prompts.**

Hypothesis 2.1: For the “real-time” reporting portion of Where I Go, over 70% of voiding events that occur during the day will be entered as confirmed by total void events reported in the prompted portion of Where I Go.

**AIM 3: Explore key factors that influence decisions for revisions of the mobile bladder app (Qualitative Aim).**

Hypothesis 3.1: Using one-to-one interviews with participants after using the app, we will identify key factors related to the Where I Go that will inform refinements needed for the Where I Go revision prototype that follows.

**AIM 4: Data collected from the Where I Go is transferred, downloaded and can be used by Scientific Data Coordinating Center (SDCC) for analysis. The main goal of this aim is to test feasibility of data transfer, management and analysis.**

Hypothesis 4.1: SDCC will work with University of Michigan-Ann Arbor Center for Health Communications Research (CHCR) to safely download and accurately analyze the data collected from the Where I Go prototype 1 to answer questions in Aim 1-3 and other exploratory analyses.

# **METHODOLOGY**

## **Overview**

Stage 3 of Where I Go development will involve in-person contacts with a research coordinator at selected research sites from the Preventing Lower Urinary Symptoms (PLUS) consortium, including the University of Michigan site. Where I Go study participants will be observed for ease in following download instructions for the app on their iOS or Android device and will be assisted as needed, with documentation of problems. Similarly, participants will be given instructions on how to interact with the Where I Go app (prototype 1) for 48 consecutive hours, and feedback on ease of use will be obtained. A follow-up survey will include a validated usability survey and open-ended questions about using the app. Modifications from the study will be incorporated into the app development process resulting in a fully refined Where I Go prototype 2 available for future additional testing.

## **Instruments embedded in the “Where I Go” Application**

There are five modules in this study: demographics, real-time assessment, check-ins, feedback surveys, and a qualitative script. All are bundled as Where I Go application data (app data), with the exception of a qualitative script. The screen by screen shot shows onboarding knowledge how the app data will be used and stored on secure servers at the University of Michigan.

**App data: Demographics**

The app will ask participants to personalize the app by adding their nickname or whatever they want. The app will gather information on age, education, ethnicity, race, income, number of toilets in home, and daily wake up/sleep schedule.

**App data: Real-time assessment**

Participants will answer questions within the app. They will be asked to input the time and location/where they urinated, how strong their urge was to urinate, whether they had to delay urination, reasons for the delay, and reasons they urinated.

**App data: Check-ins (Ecological Momentary Assessments)**

Participants will be asked to input the time and location/where they urinated or leaked, environment around them, pain if any while urinating, amount of liquid they drank, type of liquid they drank as in any caffeine, artificial sweeteners or alcohol, and strategies they used to reduce their need to urinate.

**App data: Feedback Post surveys (SUS, MARS)**

The System Usability Scale (SUS) is a validated 10-item questionnaire with 5 response categories (strongly disagree to strongly agree) and self-administered. The SUS contains questions specific to the woman’s experience in using the app questions about when they use the app, if it is difficult or easy to use, needing help or not, liking the features in the app, understandability, learning the app quickly, and having confidence. It is the most widely used questionnaire for measuring the perception of usability. It was developed in 1986 and has been used to measure software, websites, cell-phones, hardware, interactive voice response systems, paper ballots and even the yellow pages. It has been cited in over 600 research applications.

The Mobile App Rating Scale-Functionality (MARS) consists of 4 question stems with 5 responses (1- inadequate, 2-poor, 3-acceptable, 4-good, 5-excellent). The functionality questions assess performance, ease of use, navigation and gestural design. It was developed in 2015 and is a simple, objective and reliable tool for participants to provide feedback on the quality of a mobile app such as Where I Go. The MARS had excellent internal consistency and excellent interrater reliability when used to rate 50 mental health and well-being apps. (Stoyan SR, Hides L, Kavanagh et al. Mobile App Rating Scale: A new Tool for Assessing the Quality of Health Mobile Apps. JMIR Mhealth Uhealth. 2015 Jan-Mar3 (1):e27).

An additional 4 questions ask if the check-in notification was bothersome, if the questions in the app were understandable, if the user would recommend the app to others and anything else they want to share with us.

**Qualitative script**

Qualitative script to obtain feedback from participants on their experience of using Where I Go, as organized by the following general categories. These categories will provide the beginning framework for later processing/collating comments. It is expected that collating will also be organized according to purposely chosen demographics, such as age groups, whether employed outside the home, rural or urban, education level, race, etc.

Participants will be asked their experiences with the Where I Go app and perceptions using the app, such as, their experience with the way the app worked, how they app looked, the information covered on the app, the ways that might improve the app, their perceptions using the app influenced their thought of bladder health and toileting behaviors, the overall quality of the app, aspects should be changed, anything else was not discussed and they wanted to share with us.

This study also includes paper-based questionnaires:

**Feedback Post surveys (SUS, MARS)**

Paper versions of the Feedback surveys will be available for use as needed. These are imbedded in the app under Post Survey.

**Socio-demographics**

Participants will be asked their marital status, home status, employment, health insurance, any bladder problems, income, education, language, race, gender, and romantic or sexual attraction.

**Health Survey**

Participants will be asked if they were diagnosed for some medical conditions such as diabetes, and asthma, if even pregnant or give birth, menstruating status, any treatment for bladder problems, using female hormones, taking diuretic medications, and height and weight.

**Lower Urinary Tract Symptoms**

Participants will be asked about their bladder behaviors such as number of pee during day or night, any urgency, frequency, amount of the leaks, bothersome of the leaks, and triggers for leaking pee.

**Where I Go Step by Step**

Coordinators will have a step-by-step guide to assist in study processes

1. Recruitment advertisements placed
2. Interested women contact research staff
3. Screening executed (screening ID assigned) Exit form completed for non-eligible women who are thanked for their interest
4. Eligible women are scheduled for in-person visit one
5. Each participant receives a reminder letter/call/email with date/time, informed consent, directions, parking permit

**TIME POINT 1 - Visit 1**

1. Research Coordinator reviews informed consent form, participant signs and is given a copy
2. Participant is assigned an access code and instructed through the next steps:
   1. Connect to wireless network
   2. Download and install Where I Go app on smartphone
   3. Personalize app with nickname
   4. Complete demographic questions in the app
   5. Complete study packet on paper
3. Schedule 2^nd^ visit which should occur after 48 consecutive hours and within two weeks
4. Payment processed for Visit 1 ($25)

**TIME POINT 2 – between visit 1 and 2**

1. Participant: Use Where I Go app for 48 consecutive hours (inputting data)
2. Coordinator: Check participant’s dashboard for completion of Post Surveys (SUS, MARS, Additional ques.)
3. Send reminder for completion of Post survey and 2nd visit by text/phone/email

**TIME POINT 3 - Visit 2**

1. Participant completes Post survey in app if not previously done (SUS, MARS, Additional ques.)
2. Qualitative interview (written notes while audio recording)
3. Remove the app from participant’s phone
4. Payment processed for app usage and Visit 2 ($75 + $50 = $125)
5. Participant involvement complete

| **RESEARCH STAFF ONLY** |
| --- |

1. REDCap entry for appropriate instruments
2. Qualitative interview notes and audio recording data stored (locked office, locked file cabinet PI research office)
3. Identified data (name, phone, email & address) electronically stored on secure password protected local area network, School of Nursing server/password protected folder/individual ID password file
4. Analysis
5. Send participants, who have not withdrawn, Pelvic Floor newsletter with information about presentations, manuscripts and new studies until participants requests to stop

## **Recruitment**

Assuming 20% dropout, we will recruit women across 4 sites: University of Michigan, Loyola University, University of Pennsylvania and University of Alabama at Birmingham. UM will recruit 24 with other sites recruiting 12 each. Community women will be recruited through an institution-based research volunteer website (umresearch.org) and with a newsletter (Pelvic Floor Newsletter). The newsletter will be sent to previously consented study participants who signed and marked yes on the informed consent section: *Consent to be Contacted for Participation in Future Research*. Interested women will contact the research staff and the initial screening will occur by phone using the Screening Script for Recruitment.

*Study inclusion and exclusion criteria*

Inclusion criteria:

- Female sex assigned at birth
- Age ≥ 18
- Fluent in written and spoken English
- Owns a smartphone (Android or iOS), able to make phone calls, and download at least one app in the past 6 months through the app store
- Willing to respond to Where I Go prompts/texts and input data about toileting behaviors for a consecutive 48 hours
- Have to leave home at least once during the 48 hour app usage period
- Agree to two in-person visits and to bring their smartphone to the School of Nursing
- Stand/walk independently without human assistance (cane permitted)
- Will not change time zone
- Willing to add locations, must include her home

Exclusion criteria:

- Physical or mental condition that would prohibit completion of written questionnaires and interaction with the bladder application
- Institutional living arrangement (i.e. skilled nursing, long term care or rehabilitation center)
- Currently pregnant (self-reported)
- Known neurogenic or congenital bladder condition
- Unable to use toilet independently
- Male sex assigned at birth

In addition to the above inclusion/exclusion criteria, effort will be made to recruit broadly across selected demographics, such as age groups, whether employed outside the home, rural or urban, education level, race, etc. During the screening call, a unique screening site ID will be assigned. The screening ID will allow us to tally screening failures. Those that are ineligible, the coordinator will complete the Where I Go Exit Form. To those eligible for the study, the first visit will be scheduled, and the informed consent, scheduling letter with parking permit will be mailed for review. Just prior to the first visit, eligible participants will receive a visit reminder by phone/email/text.

## **Visit one** [40-60 minutes]

- 1. Research Coordinator meets participant at School of Nursing
     1. Informed consent document reviewed, questions answered, signature affixed, and copy given.
     2. Assign unique access code ID.
     3. Assist and observe participant connecting to wireless network, provide instruction for installation of the app on their personal smart phone.
     4. Participants will
        1. Personalize the app by entering a nickname.
        2. Participants will answer demographic questions in the app.
        3. Review expectations for 48 consecutive hours use of the app.
        4. Coordinator will document any questions/concerns from the participants to inform Where I Go build-team of needed revisions of the app.
        5. Complete socio-demographics form, general health and lower urinary tract symptoms questionnaires
     5. Schedule second in-person visit
     6. Coordinator processes participant compensation [$25]

## **Between visits (48 hours)**

The participant uses Where I Go app for 48 consecutive hours, starting the morning immediately following the day of 1^st^ visit. They will use the app just after they wake up and complete their use on the morning of the third day.

Participants will answer questions within the app during the 48 consecutive hours (Real-time assessments and Check-ins). The last query from Where I Go app will request that the participant fill out surveys that include the SUS, MARS and an additional 4 questions, for a total of 18 questions. This Post survey exercise is self-administered. It will take approximately 5-10 minutes to complete. Participants will be asked to complete the survey at home right after they complete using the app for 48 hours. The surveys need to be completed before the 2^nd^ in-person visit. In the case where a participant for any reason is unable to complete the post survey prior to their 2^nd^ in-person visit, the participant will be asked to do so at the beginning of the 2^nd^ visit, either within the app or alternatively by paper survey if appropriate to the situation.

Participants will be contacted by phone/email/text as a reminder for the 2^nd^ in-person visit and to complete the surveys.

## **Visit two** [40-60 minutes]

1. Participants return to School of Nursing to discuss their experience.
2. Participants will be asked several open-ended app-specific questions (Qualitative script).
3. Coordinator will take written notes. These notes will be shared only with the study and development teams to help improve the Where I Go app. Voice/audio recording will also occur to assist in note taking. The participant will be told that the audio recording will be deleted after study and data analysis has been completed.
4. Coordinator will assist in removing the app from participants’ phone
5. Coordinator processes participant compensation (48 hour app usage plus visit two [$75+$50=$125])

# **Data Retention and/or Data Destruction Plan**

***University of Michigan – site***

***University of Minnesota – SDCC***

Data will be stored on a server hosted by University of Michigan Information and Technology Services (UM-ITS). This is a standard sever configuration that provides HIPPA-compliant data security features. The database will be transferred to the University of Minnesota for analysis.

The Where I Go app does not “track” a participant’s location. Rather, when the user taps “I’m thinking about my bladder” or “I peed” in the app, a request is sent to the phone to capture the phone's current location. The current time of day is also attached to this entry. User will be asked to mark on a map what her home location is. She will have the option to mark other locations on a map where she is likely to pee during the time she is using the app. The marking of these other locations on a map is optional. The SDCC statisticians will login to the UM-ITS’ data server and export the data from the app, then download to a secure server at the University of Minnesota. The Geolocation data (geocode) is stored as latitude and longitudinal data. The geocode data will be associated with nickname and stored with all of the study data on secure drivers at the University of Michigan and the University of Minnesota (PLUS SDCC). All of the servers are password protected and can only be accessed by members of the research team.

To ensure that the application data is protected, CHCR uses virtualized servers provided by the University of Michigan’s-ITS group. The virtualized servers are housed at two redundant data centers. These data centers provide protection from lengthy outages, 24/7 staffing, restricted physical access and disaster recovery. Virtual servers are backed up automatically onto encrypted tape for recovery and security. The data centers also reduce the use of physical resources such as electricity and air conditioning.

All servers and the back end databases are password protected. The server runs the RedHat Linux 7 Enterprise operating system. Security patches and updates are downloaded and installed automatically. Each server is also protected by firewalls to restrict network access to the server. The study web application software communicates directly with the database on the same server so unencrypted participant data is not transmitted on the Internet. More details are available at <http://services.it.umich.edu/miserver>. Servers used by CHCR and the Where I Go application includes the safeguards required by HIPAA and may be used to maintain Protected Health Information. More details are available at https://www.safecomputing.umich.edu/dataguide/ under “MiServer.”

Data management in general will be facilitated by REDCap, a secure web interface for building and managing online surveys and databases with data checks used during data entry to ensure data quality. REDCap includes a complete suite of features to support HIPAA compliance, including a full audit trail, user-based privileges, and integration with the institutional LDAP server. The MySQL database and the web server will both be housed on secure servers operated by the University of Minnesota Academic Health Center’s Information Systems group (AHC-IS). The servers are in a physically secure location on campus and are backed up nightly, with the backups stored in accordance with the AHC-IS retention schedule of daily, weekly, and monthly tapes retained for 1 month, 3 months, and 6 months, respectively. Weekly backup tapes are stored offsite. The AHC-IS servers provide a stable, secure, well-maintained, and high-capacity data storage environment, and both REDCap and MySQL are widely used, powerful, reliable, well-supported systems. Access to the study's data in REDCap will be restricted to the members of the study team by username and password.

Data files generated by the app will be accessed from the University of Michigan through a secure data portal.  All workstations are password protected and reside in a secure building with further restricted access to the suite at all hours of the day.  Files will be located in Box.  Box is a cloud provided storage and collaboration service integrated with University of Minnesota (UMN) Identity Management (IDM) and administered by the Academic Health Center Information Systems office. Many system, authentication and account management controls are inherent to the UMN enterprise IDM service. In addition, two-factor authentication and complex passwords are required for all Box accounts, implemented via the UMN Duo service for two-factor authentication

When a participant accesses the study website, content is transmitted securely using the Transport Layer Security (TLS) protocol, the same protocol used to protect financial and other personal information when transmitted from a web site to a user's browser. This prevents anyone else on the network from intercepting and viewing the content that is being provided by or to the participant.

Data are provided to researchers in de-identified form, with all personally identifying information removed. Data that are provided to researchers are encrypted if it is transmitted across the Internet. At the end of the research study, all data are permanently de-identified for archive and distribution to other researchers.

***Research Coordinators Notes and Audio Recordings***

All forms and written notes taken by the research coordinator after telephone screening, during and after visits will contain site access code. The access code will be the unique identifier. The Contact Information form that contains identifiable data will be stored in locked offices at the School of Nursing with access only by study personnel. The link between a participant’s name, phone number, email and address will be kept separate from their access code.

Audio recording will occur to decrease disruptiveness of extensive note taking during the interviewer discussion process and to offer the research coordinator opportunity to review the recordings for fleshing out key participant points made that may have been lesser detailed in the moment of the conversation. The audio recordings are only for the research coordinator to have an audio-assistive mechanism for supplementing their notes. The recording will never be uploaded anywhere, it will not be transcribed, it will not be shared with anyone other than the research coordinator taking the notes. The recording will be destroyed as soon as the study has been completed and the data analyzed.

## **Statistical Considerations**

As stated in Aim 4, SDCC of PLUS will work with the CHCR build-team for Where I Go to safely download, manage and accurately analyze the data collected from Prototype 1. After the data is downloaded from University of Michigan CHCR server, the MySQL database will reside on a server hosted by the U of Minnesota’s Academic Health Center Information Systems office. This office ensures that these servers are compliant with the University’s interpretation of HIPPA regulations and other human subjects’ protections. Access to these servers requires two-factor authentication. The MySQL database will be imported to SAS data system for analysis. SDCC and/or CHCR will perform data quality checks, if any errors are detected, queries are generated which are sent back to the sites in reports with items in need of correction. These reports will be generated daily, after a time, weekly to facilitate error correction. This system allows for automated creation of datasets (de-identified or limited as necessary) with flexible frequency.

SCDD will work with University of Michigan-Ann Arbor Center for Health Communications Research (CHCR) to safely download and accurately analyze the data collected from the Where I Go prototype 1 to answer questions and other exploratory analyses.

# **Expected Outcomes of the Study**

Once Where I Go user participants have shared their experiences with the study team, the research coordinator’s notes will be reviewed by the Where I Go build-team to improve the application. These refinements are important for priming the Where I Go app’s usage in the future.

# **Duration of the Project**

Both in-person visits will last about 40-60 minutes. Actual use of Where I Go app in the 48 consecutive hours requested is estimated to take approximately one hour total across the 48 hours of its use. Participation could take up to two weeks dependent upon participants’ being able to schedule the 2^nd^ visit.

# **Compensation**

Women will be compensated for participating in this study: $25 for visit one for downloading the app and answering questions that are within the app; $75 for using or attempting to use the app for 48 hours; and $50 for completing surveys and attending visit two. Total compensation is up to $150. Participation in this project is voluntary. Subsequent to consent, participants may refuse to participate in or withdraw from the study at any time without penalty or loss of benefits to which they may be otherwise entitled. To reduce burden, free, reserved parking will be provided at the School of Nursing where the two in-person visits will occur.

# **Project Management**

The PI for this project is Janis Miller, PhD, APRN. Dr. Miller is a professor at the School of Nursing and the department of Obstetrics and Gynecology Michigan Medicine with over 25 years of research and clinical experience. She has been the PI on several NIH grants, including the current Preventing Lower Urinary Tract Symptoms (PLUS) NIH U-grant consortium study. She is a longstanding key member of the Pelvic Floor Research Group at the University of Michigan.

The Co-Investigator for this study is Lawrence An, MD. Dr. An is a seasoned researcher and, general internist and associate professor of Internal Medicine where he is the director of the Center for Health Communications Research (CHCR). The CHCR integrates behavioral science, technology, and art to create and research health interventions that inspire informed health decisions, broaden access to health information, and advance the field of health communications. Under Dr. An’s stewardship, CHCR maintains a multidisciplinary team of experts who design and develop impactful communication and engagement tools.

# **Risks and Benefits**

## **Risks**

There is a risk of loss of confidentiality as well as the possibility that personal information inadvertently may be revealed. Study app data that will contain the participant’s nickname and approximate location of her home and other places she may identify as locations where she uses the toilet, this data will be stored on password-protected servers.

Geolocation data (sites marked as locations of work, home or school) is recorded as latitude and longitudinal and is associated with participants’ unique identifier which will be kept private. The geocode data will be kept on a secure driver that can only be accessed by members of the research team. Only a single document linking the names and access code IDs will be maintained and will be password protected and locked in an office accessible only by study members. At no time will other parties such as Apple or Google have access to any data on the secure Where I Go application.

The contact information form with name, phone number email and address, will be stored in locked offices at the School of Nursing with access only by study personnel. There are little or no known risks associated with the Where I Go app or study design. Once screening failure has been entered into a separate REDCap dataset, the hard copy form will be destroyed.

The voice recordings are only for the research coordinator to have an audio-assistive mechanism for supplementing their notes. The recording will never be uploaded anywhere, it will not be transcribed, it will not be shared with anyone other than the research coordinator taking the notes. The recording will be destroyed when the study has been completed and the data analyzed.

The Where I Go app will use very little cellular data during the time in which participants will be capturing their daily voiding habits. The primary types of data usage will come from Geolocation (positional) data, which will be used during user-initiated “real time” check-ins. Survey response data will be sent from the app to the data warehouse (estimated at about 10kb per day of usage). Map data will be required (estimated at 500kb per-day). Finally, the download and installation of the Where I Go app (roughly 20mb in size). All total, we estimate the data usage won’t likely exceed 25MB for a usability tester. To minimize data usage, participants will be provided access to the MGuest wireless network while downloading and setting up the app. We anticipate this will reduce participant’s burden on their data plan by up to 22mb.

There are sensitive questions in our study that could be seen as out of place or inappropriate. These questions may trigger concerns in the participants or they might feel in answering the questions could cause repercussions. Participants do not have to answer any questions they do not want to answer.

## **Benefits**

Potential benefits to the participants include becoming more aware of how their environment influences managing their bladder needs. In addition, knowledge and information received from this study will advance the design of the Where I Go app, advance research efforts on bladder health for women and may over time lead to better prevention and treatment of lower urinary tract symptoms for women.

All participants are advised to attend both in-person visits, complete questionnaires and accomplish all app instruments. However, participants can discontinue participation at any time. The research coordinator will complete the Where I Go Exit form for each participant who discontinues their participation in the study.
