# Supplementary material for: Design of a Tool Capable of Assessing Environmental Sociocultural Physical Factors Influencing Women’s Decisions on When and Where to Toilet Within Real-World Settings: Protocol for the Build and Usability Testing of a Mobile App for Use by Community-Dwelling Women
Source: JMIR Res Protoc. 2024 Sep 18;13:e54046. doi: 10.2196/54046 (PMC11447419; doi:10.2196/54046)
Supplement: Multimedia Appendix 8 [file resprot_v13i1e54046_app8.pdf]

2U01DK106893-06 MILLER, JANIS

RESUME AND SUMMARY OF DISCUSSION: This application was submitted in response to RFA-DK-19-015 “Prevention of Lower Urinary Tract Symptoms (PLUS) Research Consortium Clinical Research Centers (U01 Clinical Trial Optional)” by Dr. Janice Miller on behalf of the University of Michigan. This application remains focused on examining factors that promote bladder health in women. The specific aims of the application are 1) determine the distribution of bladder health across the spectrum from healthy bladder to chronic lower urinary tract symptoms in U.S. adolescent and adult women, monitoring changes in bladder health over time and the life course, 2) establish and validate the optimal beverage and bladder balance across the life course by identifying beverage intake patterns and determining which are associated with good and poor ends of the bladder health spectrum and 3) explore adolescent and adult women’s lived experiences of beverage and bladder balance, including the biological, interpersonal, socio-cultural, and environmental influences. The panel indicated the longitudinal cohort study held the potential to fundamentally advance the field of women’s health. Discussion was enthusiastic and focused on the perceived strengths of the application. The overall contributions during the previous period of support, the outstanding multidisciplinary team and PI, scientific environment and innovative development of the Where I Go mobile application were all considered strong features of the application. Weaknesses were also discussed, and these included a lack of validation of the mobile application, vague analysis plans and an insufficient discussion of confounders, and a lack of attention to beverages that may be bladder irritants. Nevertheless, the strengths were thought to clearly outweigh any weaknesses and there was an excellent level of enthusiasm for the application.

DESCRIPTION (provided by applicant): The Prevention of Lower Urinary tract Symptoms (PLUS) Consortium is investing in understanding bladder health and lower-urinary track symptom prevention. The Consortium’s proposed healthy bladder definition is: “A complete state of physical, mental, and social well-being related to bladder function and not merely the absence of lower urinary tract symptoms (LUTS). Healthy bladder function permits daily activities, adapts to short-term physical or environmental stressors, and allows optimal well-being.” We are only beginning to understand the risk and protective factors that can potentiate or destroy well-being across the spectrum—from having a healthy bladder to experiencing chronic LUTS. One overarching, powerful component is the act of drinking and the state of the bladder, or what we call the Beverages to Bladder (B2B) balance. Across the life course, B2B is a fact—what goes in must come out, usually through the bladder. Despite the obvious, little data exists to guide adolescent and adult women in the what, when, and why of choosing beverage type, volume, and timing of intake as it pertains to promoting their bladder health. Our broad goal is to know what beverage intake pattern(s) relate best to a truly healthy bladder. To reach this goal, we aim to: 1) Determine the distribution of bladder health across the spectrum from healthy bladder to chronic LUTS in U.S. adolescent and adult women, monitoring changes in bladder health over time across the life course; 2) Establish and validate the optimal B2B balance across the life course by identifying beverage intake patterns and determining which are associated with the range of the bladder health spectrum; and 3) Explore adolescent and adult women’s lived experiences of B2B balance, including the biological, interpersonal, sociocultural, and environmental influences—any of which might be early facilitators or barriers to optimizing B2B balance over the life course. We will work closely within the PLUS consortium to launch a large population based observational survey study to measure bladder health, including knowledge, attitudes, and beliefs; as well as risk and protective factors. We will also have available a new data collection tool for use in this longitudinal study called Where I Go, a phone application developed at The University of Michigan. It captures beverage intake in real time, toileting experiences, and other influences affecting how adolescent and adult women manage B2B in their day-to-day lives. This data will be analyzed along with survey information and data in other pre-existing datasets. Each component offers uniqueness that can help us identify women’s variations in beverage intake patterns, and how patterns relate to bladder health across the life course. We hypothesize there is a best pattern for optimizing bladder health, but there may be many reasons

why a woman’s beverage intake differs from a best pattern. We will use adolescents and adult women focus groups combined with Where I Go to learn what influences beverage intake. Ultimately, we hope to gain new insights to inform interventions to help each woman understand her B2B balance and optimize it for bladder health.

PUBLIC HEALTH RELEVANCE: Currently, data are not available that can inform adolescent and adult women on recommendations for beverage intake pattern(s) that aid in achieving optimal bladder health. We aim to identify best beverage intake pattern(s) for preventing lower urinary tract symptoms across the life course by using an innovative mobile device application for collecting information in real time, along with a more traditional population-based survey study. Ultimately, we want to know how adolescent and adult women can optimize intake patterns to be most conducive to a truly healthy bladder, with lowest risk for developing lower urinary tract symptoms.

CRITIQUES (Note: The critiques below were prepared by the reviewers assigned to this application. These commentaries and criterion scores do not necessarily reflect the position of the authors at the close of the group discussion, nor the final majority opinion of the group, although reviewers are asked to amend their critiques if their position changed during the discussion. The resume and other initial sections of the summary statement are the authoritative representation of the final outcome of group discussion. If there is any discrepancy between the peer reviewers' commentaries and the priority/impact score on the face page of this summary statement, the priority/impact score should be considered the most accurate representation of the final outcome of the group discussion.)

CRITIQUE 1

Significance: 2
Investigator(s): 1
Innovation: 2
Approach: 3
Environment: 1

Overall Impact: This study addresses what is referred to as beverage to bladder (B2B) balance, to determine the optimal beverage intake to prevent LUTS. The authors propose studying biologic, socio-cultural and environmental factors that may promote/inhibit this balance, which they argue, serves as the tipping point between a healthy bladder and LUTS. This PLUS renewal application from the University of Michigan proposes Aims to build a longitudinal nationally representative cohort to determine the distribution of bladder health across the LUTS spectrum, to use this (and other existing) cohorts to establish and validate the optimal B2B balance, and to use focus groups to explore adolescent and adult women’s lived experience of B2B balance inclusive of facilitators and barriers to its optimization.

The study site and multidisciplinary team are both excellent and well-suited to conduct this study, and were productive during the initial PLUS study period. Additional strengths of the study include development of a longitudinal cohort of adolescent girls and women and the use of recently developed PLUS and LURN questionnaires in addition to the *Where I Go* mobile device application. However, while the authors argue that the significance of investigating beverage bladder balance is foundational, this framework may be overly simplistic and attention to other important factors should also be considered and adjusted for in analyses. Other potential weaknesses include lack of detail on projected recruitment, study power, and on several of the analysis plans. Additionally, the *Where I Go* mobile app is central to this study, and while innovative, it has not been validated. Overall, the weaknesses are relatively minor and this proposal has potential to impact our understanding of optimal beverage management across the lifespan.

1.Significance:

Strengths:

- Targets beverage and bladder health, which are potentially modifiable risk factors for LUTS prevention
- Prior focus group work (via the PLUS consortium) shows that there are conflicting ideas about how much fluid to drink. Other data demonstrate that there are barriers in schools to using the restroom and that social constructs (culture, race, socioeconomincs) may also influence beverage to bladder balance.

Weaknesses

- The concept that beverage balance is the tipping point in bladder health may be too simplistic

2.Investigator(s):

Strengths:

- Dr. Miller is a Nurse Practitioner and is a well-known researcher in this field. She is currently the PI on a PLUS grant and specializes on development of new instruments that include behavioral skills to improve bladder function in women.
- Study team also includes Dr. Baylin (Nutritional Epidemiologist), Dr. Kuzma (Nurse Practitioner specializing in adolescent health), Dr. Low (Associate Dean, midwife, and behavioral scientist), Dr Smith (Biostatistics)
- PLUS progress from first funding period (7 publications in lower/medium impact journals): building a conceptual framework, contributing to the PLUS protocol, focus groups on adolescent and adult habits, attitudes, realities, and experiences of bladder health, reviews with community stakeholders, and development and validation of a bladder health instrument.

Weaknesses

- None

3.Innovation:

Strengths:

- Use of the *Where I Go* mobile app developed by the PLUS mechanism
- Focus on novel risk factors for LUTS (toileting restrictions, social factors, etc)
- Use of clustering methodologies to identify beverage patterns and variance

Weaknesses

- None

4. Approach:

Strengths:

- Transdisciplinary approach in study planning and execution
- Will recruit a nationally representative sample of women ages 13 and older, consisting of rural and metropolitan areas.
- Uses the Bladder Health Score and *Where I Go* App, both developed by PLUS (to assess bladder health) and the LURN Symptom Index (designed to assess LUTS symptoms)

- Aim 2 uses both the proposed PLUS cohort and 2 other existing datasets (SWAN and FABULUS), allowing for incorporation of different study cohorts, however, comparing outcomes between differing groups may also be problematic in some regards
- Use of consensus K-means and hierarchical clustering to group participants in Aim 2 based on beverage intake patterns
- Separate sections on community engagement and on conflict management (for the study team) included

Weaknesses

- Details pertaining to sampling technique/process are lacking for development of the PLUS longitudinal cohort
- Analysis plans are fairly vague
- In Aim 1, it is unclear how data from the different measures will be integrated/interpreted
- Sample size is not projected, and power of the study not addressed for Aims 1 and 2
- Validity of the *Where I Go* App is lacking and a large amount of this application hinges on women (across the lifespan) filling this out consistently and completely.
- Many key confounders are not included in the longitudinal study including measures of cognition, frailty, education, medications, bladder treatments/therapies, particular attention to medical conditions that may be sensitive to fluid status, etc.
- How will potential confounding be addressed in Aim 2?

5. Environment:

Strengths:

- University of Michigan is an excellent environment for this work.
- Site (and PI) has experience with running longitudinal studies: EPI, FABULUS 1 & 2, SWAN, among others

Weaknesses

- None

Protections for Human Subjects: Acceptable Risks and/or Adequate Protections

Inclusion Plans:

- Sex/Gender: Distribution justified scientifically
- Race/Ethnicity: Distribution not justified scientifically
- For NIH-Defined Phase III trials, Plans for valid design and analysis: Not applicable
- Inclusion/Exclusion Based on Age: Distribution justified scientifically
- race/ethnicity inclusion table no projected

Vertebrate Animals: Not Applicable (No Vertebrate Animals)

Renewal: PLUS progress from first funding period (7 publications in lower/medium impact journals): building a conceptual framework, contributing to the PLUS protocol, focus groups on adolescent and adult habits, attitudes, realities, and experiences of bladder health, reviews with community stakeholders, and development and validation of a bladder health instrument.

Resource Sharing Plans: Acceptable

Budget and Period of Support: Recommend as Requested

CRITIQUE 2

Significance: 1
Investigator(s): 1
Innovation: 2
Approach: 2
Environment: 1

Overall Impact: Miller and her team of investigators present a convincing case that they can meet the requirements of the FOA and make a sustained and powerful influence on the field of women's health, especially urinary and bladder health. In addition to their involvement in the consortium's proposed longitudinal project, they also propose to conduct secondary data analyses and focus groups to inform their study of Beverage to Bladder balance, which they hope to add to the longitudinal study. In their Impact section on page 91 of the proposal, the investigators make the case that if their Beverage to Bladder balance concept is optimized and it addresses only one symptom of OAB and affects 10% of the women affected by it, there could be cost savings of 6.6 billion.

1.Significance:

Strengths:

- Proposed as part of the PLUS Consortium, the project appears to be an integral part of the Consortium demonstrating expertise in all the required areas.
- The proposed investigation to collaborate with other consortium members to do a longitudinal study examining women's bladder health across the lifespan is important to understanding the development of LUTS and avenues for prevention of the symptoms.
- The Beverage to Bladder (B2B) project proposed by the investigators adds a potentially important component to the PLUS and may lead to an intervention with respect to beverage consumption which will be assessed in real time with a mobile application developed by these researchers, called *Where I Go.*

Weaknesses

- None noted

2.Investigator(s):

Strengths:

- Janis Miller, principal investigator of the proposed project is incredibly well-suited to lead this project. She is an expert in women's health across the life cycle, especially as it relates to the pelvic floor. She developed and tested "The Knack," a now famous method of preventing urinary leakage. She has been involved in numerous other studies and the principal investigator of SWAN a longitudinal study of more than 20 years' duration.
- Ana Baylin is an MD with a Dr.PH and a nutritional epidemiologist who has done extensive work with community groups and with beverage intake. Elizabeth Kuzma is a clinician expert in the care of adolescents. Lisa Kane Low, an expert in midwifery practice and in qualitative methods who cp-led the PLUS qualitative investigation of perceptions of bladder health. Abigail Smith is a research scientist at the Ann Arbor Research Collaborative, specializing in biostatistics with experience working on the PLUS project and other projects on LUTS.

Weaknesses

- It does not appear that PI Miller was as active in producing the manuscripts for the consortium as some other PI's.

3.Innovation:

Strengths:

- A major innovation which applies to the entire consortium is to shift the research framework from bladder disease and deficits to bladder health and selfcare. The PLUS investigators also propose to shift the research orientation from clinical research to community/population-based research by empowering women to support their own bladder health before problems arise.
- In proposing the novel ideas with respect to Beverage to Bladder (B2B) study, this project has the potential to increase our knowledge about the causes of LUTS and about beverage advice in the treatment of LUTS. Investigators point out the beverage explosion and its likely effect on bladder symptoms.
- The use of *Where I Go,* a phone application that is planned to be introduced into the longitudinal study at all sites….

Weaknesses

- In limiting the fluids monitored to those that might be bladder irritants (caffeine, sugar substitutes, alcohol, and carbonated beverages), the study might miss fluids or supplements that participants might use to treat or prevent LUTS, such as cranberry juice.

4. Approach:

Strengths:

- The Michigan team has successfully recruited a racially and socio-economically diverse sample and have included the identification and enumeration of Asian subjects in their sample.
- The application presents a realistic plan for including community members to work with community organizations that serve adolescents, pregnant women and families especially to study females' lived experience of Beverage to Bladder balance.
- The researchers outline a plan to study the relationship between beverage intake and bladder problems. The researchers have supported the execution of a longitudinal study required by the FOA and proposed adding to the longitudinal data collection the real time collection of data over

48 hours using the *Where I Go* phone app which assesses activities related to urination, but also beverage intake.

- The investigators propose a Conflict Management Plan, including the election of an ombuds who will mediate conflicts and deal with minor issues before they become major ones. This role is said to be especially helpful to the small or new voice in the group. In a group the size of the Consortium with many senior investigators, it is a good idea to have this role identified and codified.
- The investigators' leadership plan will draw the policies and principles in the PLUS manuals, which were built by PI Miller and PI Brubaker from Loyola and which received the full support of the Consortium.
- Plans for biosample collection and storage were outlined as were plans for sharing with the Consortium and NIDDK both data and resources, including the sharing of the Where I Go application for data collection from cell phones, which was invented and continues development at the University of Michigan.

Weaknesses

- Although the approach used in the longitudinal study has yet to be decided, these investigators plan to introduce a phone application to collect real time data for 48 hours. Use of these apps take special training of the subjects, which is not described in this application.

5. Environment:

Strengths:

- The scientific environment at the University of Michigan is exceptionally strong. This study emanates from the School of Nursing which demonstrates a strong commitment both to research in general and to this study in particular.

Weaknesses

- None noted.

Protections for Human Subjects: Acceptable Risks and/or Adequate Protections

Data and Safety Monitoring Plan (Applicable for Clinical Trials Only):

Not Applicable (No Clinical Trials)

Inclusion Plans:

- Sex/Gender: Distribution justified scientifically
- Race/Ethnicity: Distribution justified scientifically
- For NIH-Defined Phase III trials, Plans for valid design and analysis: Not applicable
- Inclusion/Exclusion Based on Age: Distribution justified scientifically

Vertebrate Animals: Not Applicable (No Vertebrate Animals)

Renewal: This is a renewal application of high quality.

Resource Sharing Plans: Acceptable

Budget and Period of Support: Recommend as Requested

CRITIQUE 3

Significance: 2
Investigator(s): 2
Innovation: 2
Approach: 3
Environment: 3

Overall Impact: The PLUS consortium was established to address a need to promote bladder health and to identify preventive strategies for those at risks of developing LUTS across life span. This is an admirable goal and will advance LUTS research by focusing on preventive strategies and represent a paradigm shift in LUTS research. The Michigan team has played critical roles in PLUS 1 transdisciplinary research and has published the key findings from the SHARE qualitative study. In terms of innovation, collecting real time and context sensitive information using the PLUS “Where I Go” mobile apps (under development) and using it for B2B (bladder to bathroom) balance research is innovative. Also, combining the mobile app B2B data with the B2B focus group data will likely lead to future interventional trials since B2B behavior is very modifiable. There are a few identified weaknesses: 1) although there are over 11 papers from PLUS 1, many papers represent foundation work; 2) there is a lack of specific details of the ATTRIBUTES study, 3) the BHI and KAB instruments have not been published, and the VIEW and CLEAR studies have not been published yet, 4) the B2B aims ignored the contribution of bladder irritant foods besides just fluid and beverages, and it is also unclear if there will be detailed analysis in terms of underlying bladder irritants (e.g., amount of caffeine in fluid). Overall the proposal is excellent.

1.Significance:

Strengths:

- The PLUS consortium was established to address a need to promote bladder health and to identify preventive strategies for those at risks of developing LUTS across life span. This is an admirable goal and will advance LUTS research by focusing on preventive strategies and represent a paradigm shift in LUTS research.
- The foundational tools to embark on future interventional trials to promote bladder health and hopefully prevent LUTS are being developed in PLUS 1 and will continue in PLUS 2.
- The proposed studies (ATTRIBUTES, the PLUS mobile bladder apps) should be able to broadly address relevant factors in the general population and will likely support future interventional trials and prevention trials of LUTS as a long term goal.
- The B2B (Bladder to Bathroom) studies proposed may have impact leading to future interventional trials to modulate bladder and bathroom behaviors via fluid manipulation.

Weaknesses

-

2.Investigator(s):

Strengths:

- The Michigan team has played critical roles in PLUS 1 transdisciplinary research and has demonstrated abilities to interact successfully with other PLUS sites.
- The PI (Dr. Miller) has expertise in LUTS and multidisciplinary research and has contributed to the development of the PLUS "Where I Go" bladder app as co-lead. The PLUS bladder app will be a key component of the ATTRIBUTE and B2B studies.
- Co-I: Dr. Low is a co-lead of the SHARE qualitative focus group study. Dr. Low is the last author of the SHARE paper that contained actual data and contributed to the VIEW and CLEAR studies and community research. Dr. Baylin is a nutritional epidemiologist with expertise on beverage intake data. Dr. Kuzma specializes in adolescence health. Overall there is a good mix of expertise appropriate for the PLUS 2 and B2B aims.
- Retaining the team of investigators from PLUS 1 will hasten progress in PLUS 2.

Weaknesses

-

3.Innovation:

Strengths:

- The shift from studying patients already affected by LUTS (the current research paradigm) to community efforts to identifying protective factors and risk factors for LUTS development and progression, and engagement to the community and institutions (e.g., "gatekeepers" in toilet access) represent a paradigm shift.
- The concept that "bladder health" represents more than a lack of bothersome LUTS symptoms is a conceptual innovation.
- Collecting real time bladder habits information using the PLUS "Where I Go" mobile apps (under development) is innovative.
- The use of the PLUS "Where I go" bladder app to capture real time, context-sensitive, detailed information on bathroom use and fluid intake is a major innovation of the grant application.

Weaknesses

-

4. Approach:

Strengths:

- One of the existing PLUS 1 sites and transdisciplinary team that have done important foundational work in the development of the conceptual model, bladder health definition, formative qualitative work (SHARE focus groups), and the PLUS bladder mobile app.
- Demonstrated ability to recruit for the qualitative study (focus groups) from PLUS 1 (the CLEAR study). Dr. Low oversaw the SHARE qualitative study (focus groups) from PLUS 1. She is the last author of the SHARE paper that contained actual data.
- Letter of support from every one of the current PLUS consortium sites, outlining how they have successfully collaborated and how they plan to work together in the future.
- Progress report: more than 11 accepted papers from PLUS 1, many are on foundational work. There was active participation from this study team on many of the papers.
- The implementation of the PLUS bladder app "Where I Go" very impactful and timely. Not only would it contribute to the ATTRIBUTE study, it would provide data to the B2B studies. The results may lead to future interventional trials since B2B behavior is very modifiable.

- Incorporation of the LURN-SI-29 instrument to measure transition to LUTS is a plus.
- Using focus groups in Aim 3 to further explore adolescence and women's lived experiences of B2B balance, including biological, interpersonal, soci-cultural, and environmental influences, as well as facilitators and barriers to optimize B2B is a plus.
- There is a detailed description of community engagement at the local community.

Weaknesses

- Progress report: although there are over 11 papers fro PLUS 1, many papers represent foundation work, like the rationale behind PLUS, conceptual model, terminology, bladder health definition, re-analysis of existing database, and the protocol of the SHARE study. There was only one paper that actually published results from participants from the SHARE focus groups.
- There is a lack of specific details of the ATTRIBUTES study (the main work of the proposal). It is not clear what the BHI and BHS (bladder health index/scale) look like, it is not clear what the KAB (knowledge, attitudes, and belief) instrument would look like, none of these have been published. The target population, recruitment strategies, length of follow up, and study procedures are not defined. How many subjects will be needed? Power calculation for that many subjects? Is the follow up long enough to see a transition from healthy bladder to the development of LUTS? To this reviewer's knowledge, there are no publications or results from the VIEW and CLEAR studies from which the ATTRIBUTE study derives from. When can the ATTRIBUTE study launch given that the BHI, BHS, KAB instruments may not be ready, and VIEW and CLEAR is ongoing?
- Specific to the B2B aims: There is lack of consideration of the contribution of bladder irritants foods (e.g., spicy food, citrus fruits, tomato or tomato-based food, food containing artificial sugars, chocolate) to B2B. The focus of the grant application has been on fluid and beverages instead. Also, it is unclear if there will be detailed analysis of the fluid and beverages in terms of underlying bladder irritants (e.g., amount of caffeine in fluid).

5. Environment:

Strengths:

- Excellent clinical support and recruitment infrastructure given the proven abilities to recruit the specific population in PLUS 1.

Weaknesses

- Ann Arbor may not be the perfect place to recruit a racially, ethically, and socio-economically diverse group of participants given the sub-urban and college town setting

Protections for Human Subjects: Acceptable Risks and/or Adequate Protections

Data and Safety Monitoring Plan (Applicable for Clinical Trials Only):

Not Applicable (No Clinical Trials)

Inclusion Plans:

- Sex/Gender: Distribution justified scientifically
- Race/Ethnicity: Distribution justified scientifically
- For NIH-Defined Phase III trials, Plans for valid design and analysis:
- Inclusion/Exclusion Based on Age: Distribution justified scientifically

Vertebrate Animals: Not Applicable (No Vertebrate Animals)

Renewal: See comments on progress report section above

Resource Sharing Plans: Acceptable

Budget and Period of Support: Recommend as Requested

THE FOLLOWING SECTIONS WERE PREPARED BY THE SCIENTIFIC REVIEW OFFICER TO SUMMARIZE THE OUTCOME OF DISCUSSIONS OF THE REVIEW COMMITTEE, OR REVIEWERS' WRITTEN CRITIQUES, ON THE FOLLOWING ISSUES:

PROTECTION OF HUMAN SUBJECTS: ACCEPTABLE

INCLUSION OF WOMEN PLAN: ACCEPTABLE

INCLUSION OF MINORITIES PLAN: ACCEPTABLE

INCLUSION OF CHILDREN PLAN: ACCEPTABLE

COMMITTEE BUDGET RECOMMENDATIONS: The budget was recommended as requested.

Footnotes for 2 U01 DK106893-06; PI Name: MILLER, JANIS M

NIH has modified its policy regarding the receipt of resubmissions (amended applications).See Guide Notice NOT-OD-18-197 at https://grants.nih.gov/grants/guide/notice-files/NOT-OD-18-197.html. The impact/priority score is calculated after discussion of an application by averaging the overall scores (1-9) given by all voting reviewers on the committee and multiplying by 10. The criterion scores are submitted prior to the meeting by the individual reviewers assigned to an application, and are not discussed specifically at the review meeting or calculated into the overall impact score. Some applications also receive a percentile ranking. For details on the review process, see http://grants.nih.gov/grants/peer_review_process.htm#scoring.

MEETING ROSTER

National Institute of Diabetes and Digestive and Kidney Diseases Special Emphasis Panel
NATIONAL INSTITUTE OF DIABETES AND DIGESTIVE AND KIDNEY DISEASES
Women's Bladder Health applications PLUS

ZDK1 GRB-M (M2)
04/16/2020

Notice of NIH Policy to All Applicants: Meeting rosters are provided for information purposes only. Applicant investigators and institutional officials must not communicate directly with study section members about an application before or after the review. Failure to observe this policy will create a serious breach of integrity in the peer review process, and may lead to actions outlined in NOT-OD-14-073 at https://grants.nih.gov/grants/guide/notice-files/NOT-OD-14-073.html and NOT-OD-15-106 at https://grants.nih.gov/grants/guide/notice-files/NOT-OD-15-106.html, including removal of the application from immediate review.

CHAIRPERSON(S)

LAI, HING HUNG HENRY, MD
ASSOCIATE PROFESSOR
DEPARTMENT OF SURGERY
WASHINGTON UNIVERSITY
SCHOOL OF MEDICINE
ST. LOUIS, MO 63110

MEMBERS

ALPERIN, MARIANNA, MD, MS
ASSOCIATE PROFESSOR
DEPARTMENT OF OBSTETRICS AND GYNECOLOGY
DIVISION OF FEMALE PELVIC MEDICINE AND
RECONTRUCTIVE SURGERY
UNIVERSITY OF CALIFORNIA, SAN DIEGO
SAN DIEGO, CA 92121

ASHIKAGA, TAKAMARU, PHD
PROFESSOR AND DIRECTOR EMERITUS
MEDICAL BIOSTATISTICS
UNIVERSITY OF VERMONT
LARNER COLLEGE OF MEDICINE
BURLINGTON, VT 05405

BEBU, IONUT, PHD
ASSOCIATE RESEARCH PROFESSOR
THE BIOSTATISTICS CENTER
MILIKEN INSTITUTE SCHOOL OF PUBLIC HEALTH
THE GEORGE WASHINGTON UNIVERSITY
WASHINGTON D.C., DC 20852

CHEN, YIYI, PHD
ASSOCIATE PROFESSOR
DEPARTMENT OF BIOSTATISTICS
OREGON HEALTH AND SCIENCE UNIVERSITY
SCHOOL OF PUBLIC HEALTH
PORTLAND, OR 92739

HADJIFRANGISKOU, MARIA, PHD
ASSISTANT PROFESSOR
DEPARTMENT OF PATHOLOGY, MICROBIOLOGY AND
IMMUNOLOGY
VANDERBILT UNIVERSITY MEDICAL CENTER
NASHVILLE, TN 37232

HANDA, VICTORIA LYNN, MD, MS
PROFESSOR
DEPARTMENT OF OBSTETRICS AND
GYNECOLOGY
JOHNS HOPKINS UNIVERSITY
SCHOOL OF MEDICINE
BALTIMORE, MD 21287

HOMMEL, KEVIN, PHD
PROFESSOR
DEPARTMENT OF PEDIATRICS
UNIVERSITY OF CINCINNATI COLLEGE OF MEDICINE
CINCINNATI, OH 45229

MARSHALL, LYNN M., SCD
ASSOCIATE PROFESSOR
DEPARTMENT OF ORTHOPAEDICS AND REHABILITATION
OREGON HEALTH AND SCIENCE UNIVERSITY
PORTLAND, OR 97239

MOALLI, PAMELA A., MD, PHD
PROFESSOR
DEPARTMENT OF OBSTETRICS, GYNECOLOGY
AND REPRODUCTIVE SCIENCES
MAGEE-WOMENS RESEARCH INSTITUTE
PITTSBURGH, PA 15213

NELSON, DAVID EMMET, PHD
ASSOCIATE PROFESSOR
DEPARTMENTS OF IMMUNOLOGY AND
MICROBIOLOGY
SCHOOL OF MEDICINE
INDIANA UNIVERSITY
INDIANAPOLIS, IN 46202

OCONNELL, KATHLEEN ANN, PHD
PROFESSOR
DEPARTMENT OF HEALTH AND BEHAVIOR STUDIES
TEACHERS COLLEGE
COLUMBIA UNIVERSITY
NEW YORK, NY 10027

SUSKIND, ANNE M., MD, MS
ASSOCIATE PROFESSOR
DEPARTMENT OF UROLOGY
UNIVERSITY OF CALIFORNIA- SAN FRANCISCO
SAN FRANCISCO, CA 94143

VAN DEN EEDEN, STEPHEN K., PHD
SENIOR INVESTIGATOR
DIVISION OF RESEARCH
KAISER PERMANENTE
OAKLAND, CA 94612

SCIENTIFIC REVIEW OFFICER

MORRIS, RYAN G., PHD
SCIENTIFIC REVIEW OFFICER
REVIEW BRANCH, DIVISION OF EXTRAMURAL ACTIVITIES
NIDDK, NATIONAL INSTITUTES OF HEALTH
ROOM 7015, 6707 DEMOCRACY BOULEVARD
BETHESDA, MD 20892-2542

Consultants are required to absent themselves from the room during the review of any application if their presence would constitute or appear to constitute a conflict of interest.
